# Chromones as Photocatalyzed HAT Reagent and Michael Acceptor for Direct C–H Alkylation of 2-Substituted 4-Chromanones

**DOI:** 10.34133/research.1250

**Published:** 2026-05-11

**Authors:** Ya Xiao, Junrong Zhang, Wujun Jian, Ya Zhou, Xiang Zhou, W. M. W. W. Kandegama, Song Yang

**Affiliations:** ^1^ State Key Laboratory of Green Pesticides, Key Laboratory of Green Pesticide and Agricultural Bioengineering, Ministry of Education, Center for R&D of Fine Chemicals of Guizhou University, Guiyang 550025, China.; ^2^ Department of Horticulture and Landscape Gardening, Faculty of Agriculture and Plantation Management, Wayamba University of Sri Lanka, Makandura, Gonawila, Sri Lanka.

## Abstract

4-Chromanone is a biologically important heterocyclic scaffold found in natural products and pharmaceuticals. Traditional syntheses of 2-substituted 4-chromanones often rely on high-temperature peroxide initiation, transition-metal catalysis, or complex photocatalytic systems, limiting functional-group tolerance and sustainability. Herein, we report a Giese-type strategy for direct C–H functionalization of chromone derivatives, where chromones act as both substrates and hydrogen atom transfer (HAT) reagents to active inert C(sp^3^)–H bonds and P–H bonds under mild, metal-free conditions. Upon light irradiation, chromones form triplet states that mediate HAT for diverse substrates, including ethers, esters, alcohols, alkanes, sulfides, and arylphosphines. The resulting radicals are captured by ground-state chromones, efficiently affording 2-substituted 4-chromanones. Mechanistic studies and density functional theory calculations support the radical-mediated pathway, and the method avoids the use of metal catalysts, external redox mediators, or photocatalysts, highlighting its scalability and sustainable potential.

## Introduction

**Fig. 1.**
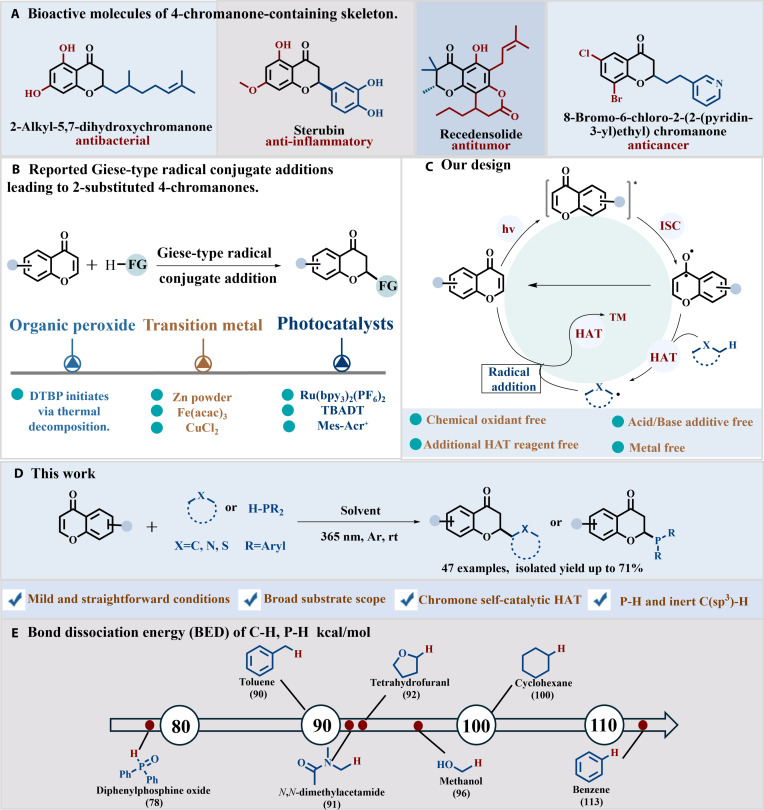
(A) Bioactive molecules of 4-chromanone-containing skeleton. (B) Reported Giese-type radical conjugate additions leading to 2-substituted 4-chromanones. (C) Catalytic cycle of chromone serving as both HAT reagent and substrate. (D) This work. (E) BED of C–H, P–H kcal/mol.

4-Chromanone constitutes an important oxygen-containing heterocyclic scaffold, whose derivatives exhibit diverse biological activities and are widely found in numerous biologically active molecules [1–4]. In particular, 2-substituted 4-chromanone derivatives display a broad range of bioactivities, including antibacterial [5,6], anti-inflammatory [7], antitumor [8], and antiproliferative effects against breast and lung carcinoma cell lines [9] (Fig. 1A). The broad biological activities of these compounds have drawn attention. Researchers are focusing on developing simple and efficient methods to synthesize 2-substituted 4-chromanones. Chromones belong to the class of *α,β*-unsaturated carbonyl compounds. In Giese-type reactions, they act as effective Michael acceptors to construct 2-substituted 4-chromanones. Traditional Giese additions using chromone substrates generally require either high-temperature initiation with di-*tert*-butyl peroxide [10–13], reactions involving transition metals such as zinc [14,15] and iron [16] (Fig. 1B).

In comparison, photocatalytic strategies offer a more sustainable alternative by employing light energy to drive chemical transformations under mild conditions. Through the rational design of photocatalysts and their active sites [17,18], diverse photocatalytic pathways have been developed [19,20]. Previous studies have reported the synthesis of 4-chromanone derivatives using various photocatalytic strategies [21–23], among which the combination of photocatalysis with hydrogen atom transfer (HAT) has emerged as an efficient approach. Photocatalytic HAT has been employed to directly construct a variety of carbon–carbon and carbon–heteroatom bonds [24–29], particularly enabling functionalization of inert C(sp^3^)–H bonds under mild conditions [30–35]. Representative examples include CuCl_2_-catalyzed synthesis of 2-substituted 4-chromenones via a photoinduced ligand-to-metal charge transfer (LMCT) pathway [36]. Ru(bpy_3_)_2_(PF_6_)_2_-catalyzed doubly decarboxylative Giese addition reported by Moczulski et al. [37], as well as light-driven C2-alkylation of chromones using decatungstate (TBADT) and zwitterionic acridinium-based HAT reagents developed by Entgelmeier et al. [38] and Qiao et al. [39], respectively (Fig. 1B). Additional photocatalytic HAT mediators include eosin Y [40,41] and metal complexes like CuCl_2_ [42] and FeCl_3_ [43,44]. Moreover, heteroatom-centered radicals derived from sulfur or nitrogen compounds [45–49] have been employed to promote radical additions to unsaturated systems. Despite these advances, current photocatalytic HAT systems are often limited by short excited-state lifetimes and inadequate site selectivity [50–53]. Reduced reaction efficiency caused by competing single-electron or energy-transfer processes remains another challenge [54,55]. Sulfur- and nitrogen-centered HAT reagents often rely on precious photocatalysts, increasing cost and reducing sustainability. These factors limit the broader application of photocatalytic HAT. Developing efficient, low-cost, metal-free organic HAT reagents is a promising direction.

Aromatic carbonyl compounds have attracted attention as photosensitizers and as HAT mediators because of their favorable photophysical properties [56,57]. Since the seminal works reporte by Norrish and Bamford in 1937 [58], the photoexcitation behavior of carbonyl compounds has been widely applied in synthetic transformations [59–61]. Upon light absorption, these compounds can undergo intersystem crossing to triplet excited states with diradical character [62], which allows hydrogen atom abstraction from a variety of substrates [63–69] and enables carbonyl-driven self-coupling without the need for external photocatalysts [70,71]. Using aromatic carbonyl compounds as both substrates and HAT mediators in Giese-type reactions remains relatively unexplored.

Herein, we designed photoexcited chromones as intrinsic HAT reagent, activating both P–H bonds and C–H bonds, thereby enabling Giese-type addition reactions between ground-state chromones and substrates such as alkanes, ethers, esters, and arylphosphines (Fig. 1C and D). Compared with previous studies that rely on unstable peroxides, harsh reaction conditions, limited functional group compatibility, excessive involvement of metal reagents, and complex, costly photocatalytic systems, all of which are unfavorable for the development of green chemistry, this work utilizes chromones as Michael acceptors to construct 2-substituted 4-chromanones via Giese-type addition. This reaction system avoids the use of metal catalysts, unstable peroxides, external photocatalysts, redox reagents, or additional HAT mediators; can be performed under mild conditions; and demonstrates a chromone-mediated self-sustained HAT reaction mode, broad substrate applicability, and good alignment with green chemistry principles.

## Results and Discussion

### Optimization of the reaction conditions

To assess the feasibility of chromones as dual-functional substrates and intrinsic HAT mediators in Giese-type additions, chromone (**1a**) and benzodioxole (**2a**) were selected as model substrates for reaction condition optimization (Table 1). Initial screening of irradiation wavelengths (Table S1) demonstrated that irradiation with a 9-W 365-nm light-emitting diode (LED) light source for 12 h afforded the target product **3a** in 59% yield (Table 1, entry 1). Chromone compounds require light of sufficient energy to excite them to the triplet state necessary for the reaction, and irradiation at 365 nm efficiently generates this triplet state, thereby facilitating the HAT process. Subsequent solvent screening (Table S2) identified the moderate polarity solvent dichloroethane (DCE) as optimal, which also gave **3a** in 59% yield (Table 1, entry 1). Through comparison of the fluorescence intensities of excited-state chromones in different solvents, it was found that solvents with either excessively high or low polarity are unfavorable for stabilizing the excited state, whereas solvents with moderate polarity, such as DCE, effectively stabilize the excited state (Fig. S1). This observation is consistent with the experimental results. Thus, solvent polarity can affect the excited-state lifetime of chromones, which in turn influences HAT efficiency [72]. Further optimization of the loading of **2a** revealed that increasing its amount to 15 equivalents enhanced the yield of **3a** to 67% (Table S3, entry 8), indicating that the high concentration of substrate contributes to increasing the HAT event’s probability of the triplet state chromone with substrate, reducing self-coupling of exciting state chromone. To further inhibit this self-coupling, we tried to increase the volume of the reaction solvent to decrease the concentration of the exciting state chromone. Preliminary screening of the reaction solvent volume (Table S4, entry 7) revealed that employing 6 ml of DCE delivered the target product **3a** in 73% yield (Table 1, entry 12). Variation of the irradiation duration confirmed 12 h as the optimal reaction time (Table S5), ensuring sufficient photon absorption while avoiding decomposition due to overexposure. Subsequently, temperature was evaluated over the range of 0 to 40 °C (Table S6). The reaction exhibited the highest efficiency at room temperature, while lower temperatures likely reduced the efficiency of HAT, and higher temperatures may decrease the stability of the triplet chromone excited state. The effect of LED power (3 to 10 W) on the yield of **3a** was evaluated (Table S7), reaching a maximum at 9 W; the higher power slightly decreased the yield, likely due to side reactions. On the basis of these results, the standard reaction conditions for subsequent investigations were established as follows: chromone (**1a**, 0.2 mmol) and benzodioxole (**2a**, 3 mmol) in DCE (6 ml) under an argon atmosphere irradiated with a 9-W 365-nm LED light source for 12 h.

**Table 1.** Optimization of the reaction conditions. A mixture of 1a (0.2 mmol) and 2a in the solvent was irradiated with a 9-W light-emitting diode at room temperature under an argon atmosphere. Yields were determined by ^1^H nuclear magnetic resonance spectroscopy using 1,4-dimethoxybenzene as an internal standard.

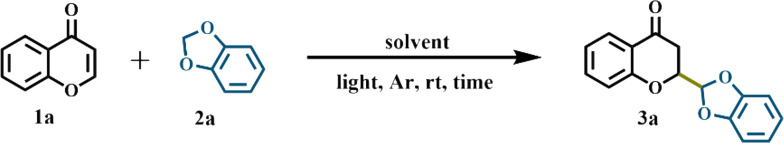					
Entry	**2a**/mmol	Light	Solvent	Time/h	Yield%
1	2.0	365 nm	2 ml DCE	12	59
2	2.0	390 nm	2 ml DCE	12	29
3	2.0	405 nm	2 ml DCE	12	27
4	2.0	365 nm	2 ml MeCN	12	9
4	2.0	365 nm	2 ml EA	12	21
5	2.0	365 nm	2 ml DCM	12	54
6	2.4	365 nm	2 ml DCE	12	62
7	2.8	365 nm	2 ml DCE	12	65
8	3.0	365 nm	2 ml DCE	12	67
9	3.2	365 nm	2 ml DCE	12	66
10	3.6	365 nm	2 ml DCE	12	62
11	3.0	365 nm	4 ml DCE	12	68
12	3.0	365 nm	6 ml DCE	12	73 (66) ^a^
13	3.0	365 nm	8 ml DCE	12	56
14	3.0	365 nm	6 ml DCE	10	64

^a^ Isolated yield.

### Scope of the reaction

**Fig. 2.**
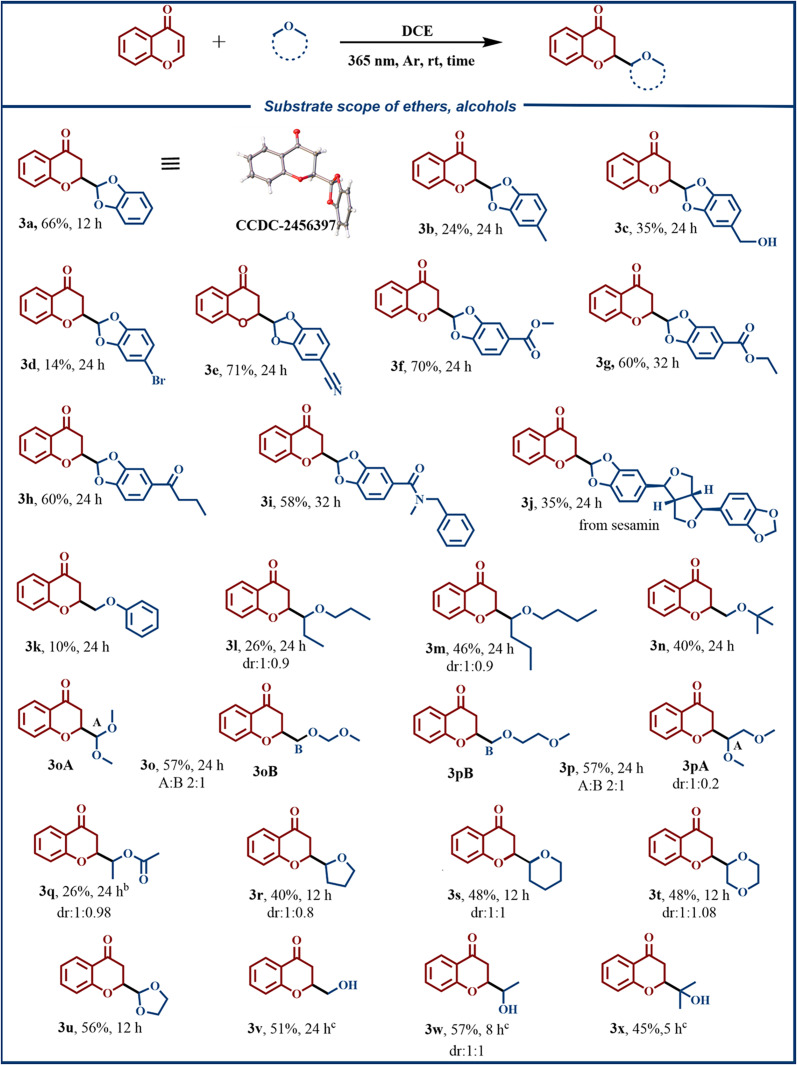
Substrate scope. ^a^ Reaction conditions: A mixture of 1a (0.2 mmol) and 2 (3 mmol) in DCE (6 ml) was irradiated with a 9-W light-emitting diode (LED) (λ = 365 nm) at room temperature under an argon atmosphere. Isolated yield and reaction times are indicated below each product. ^b^ 1a (0.2 mmol) in ethyl acetate (2 ml). ^c^ 1a (0.2 mmol) and B(OH)_3_ (0.3 mmol) in the corresponding alcohol (2 ml).

Under the optimized conditions, we explored the substrate scope of ether derivatives (Fig. 2). Unsubstituted benzodioxole reacted smoothly to afford product **3a** in 66% yield, and its *S*-configuration was unambiguously confirmed by x-ray crystallographic analysis. The introduction of electron-donating groups or halogen substituents (**3b** to **3d**) resulted in diminished yields, whereas electron-withdrawing groups enhanced the reactivity, delivering **3e** to **3i** in fair to good yields. Sesamin was also compatible with the reaction system, affording **3j**. Notably, the substrate scope was successfully expanded to various aliphatic and cyclic ethers, including anisole, propyl ether, butyl ether, *tert*-butyl methyl ether, methylal, dimethoxyethane, ethyl acetate, tetrahydrofuran, and 1,4-dioxane, all of which exhibited excellent compatibility. When ethyl acetate as a solvent was added in bulk, the yield of **3q** improved markedly compared to equimolar conditions. It afforded only a trace product. Diastereomers **3r** to **3t** were readily separable by column chromatography. Substrates with multiple reactive sites reacted selectively at a single C–H bond. This process produced **3u** as the only product. Additionally, the reaction was applicable to simple alcohols upon the addition of boric acid to facilitate *α*-C–H bond activation [73,74] (Table S8), providing **3v** to **3x** in moderate yields.

**Fig. 3.**
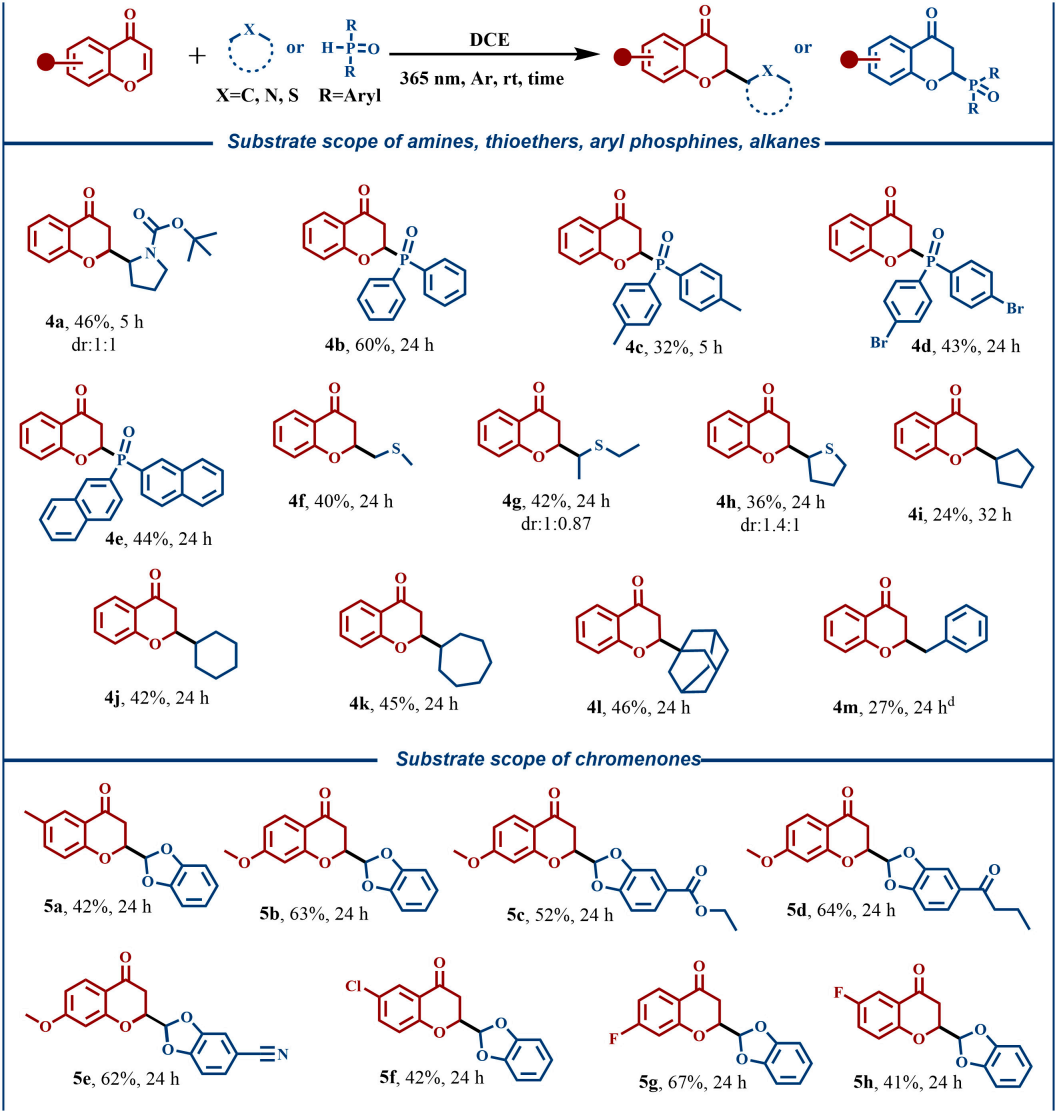
Substrate scope. ^a^ Reaction conditions: A mixture of 1 (0.2 mmol) and 2 (3 mmol) in DCE (6 ml) was irradiated with a 9-W LED (λ = 365 nm) at room temperature under an argon atmosphere. Isolated yield and reaction times are indicated below each product. ^d^ 1a (0.2 mmol) in toluene (2 ml).

To further broaden substrate diversity, we explored N-, P-, and S-containing compounds (Fig. 3), obtaining products **4a** to **4h** in moderate yields. Considering the higher C–H bond dissociation energies (BDEs) of cyclic alkanes, we evaluated the HAT capability of photoexcited triplet-state chromone with cyclopentane, cyclohexane, cycloheptane, and adamantane, leading to **4j** to **4l** in moderate yields. Toluene was also tolerated under standard conditions, affording **4m**. We then examined substituted chromones bearing modifications on the benzene ring, which furnished derivatives **5a** to **5h** in 41% to 67% yields. When chromones bear phenol, carboxylic acid, or nitro substituents on the benzene ring, corresponding target products were not observed. It is proposed that phenol and carboxylic acid groups may quench the HAT process via labile hydrogen atoms, while nitro substituents alter the electron density of the substrate, thereby affecting its excited state and reducing its ability to participate in the reaction.

**Fig. 4.**
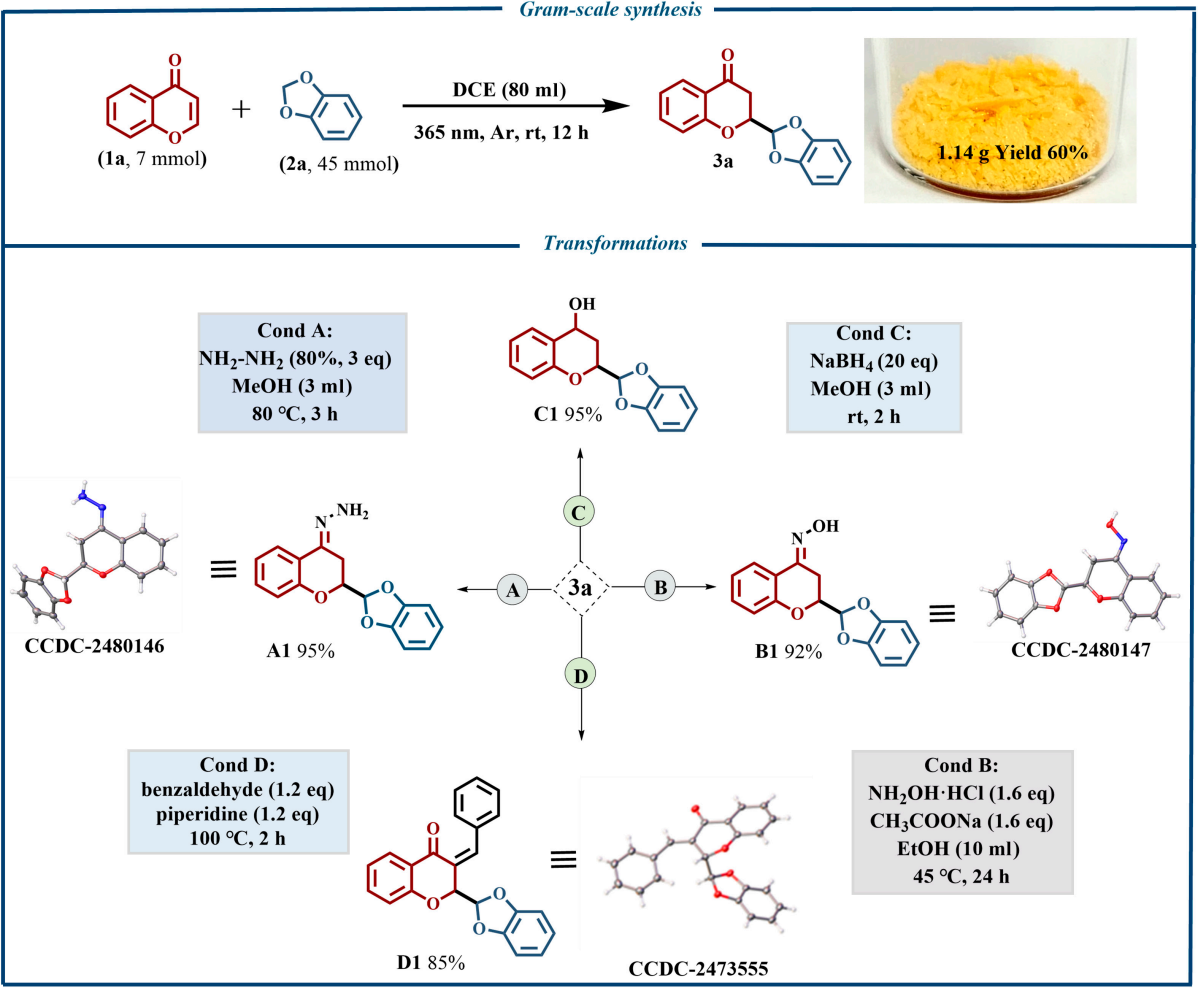
Gram-scale synthesis and transformations.

To assess the practical synthetic utility of this protocol, a gram-scale reaction of **1a** (7 mmol) with **2a** (45 mmol) was performed in anhydrous DCE (80 ml) under argon. Irradiation at 365 nm for 12 h gave **3a** in 60% isolated yield after purification (Fig. 4). Further transformations of **3a** were carried out (Fig. 4). The carbonyl group of **3a** can be effectively converted into idenehydrazine (**A1**), aldehyde oxime (**B1**), and hydroxyl (**C1**) structures with excellent yields, and **3a** can be condensed with an aldehyde to yield the target compound **D1**. Single-crystal x-ray diffraction further verified that compounds **A1**, **B1**, and **D1** exhibit the E configuration.

### Investigation and proposal of the reaction mechanism

**Fig. 5.**
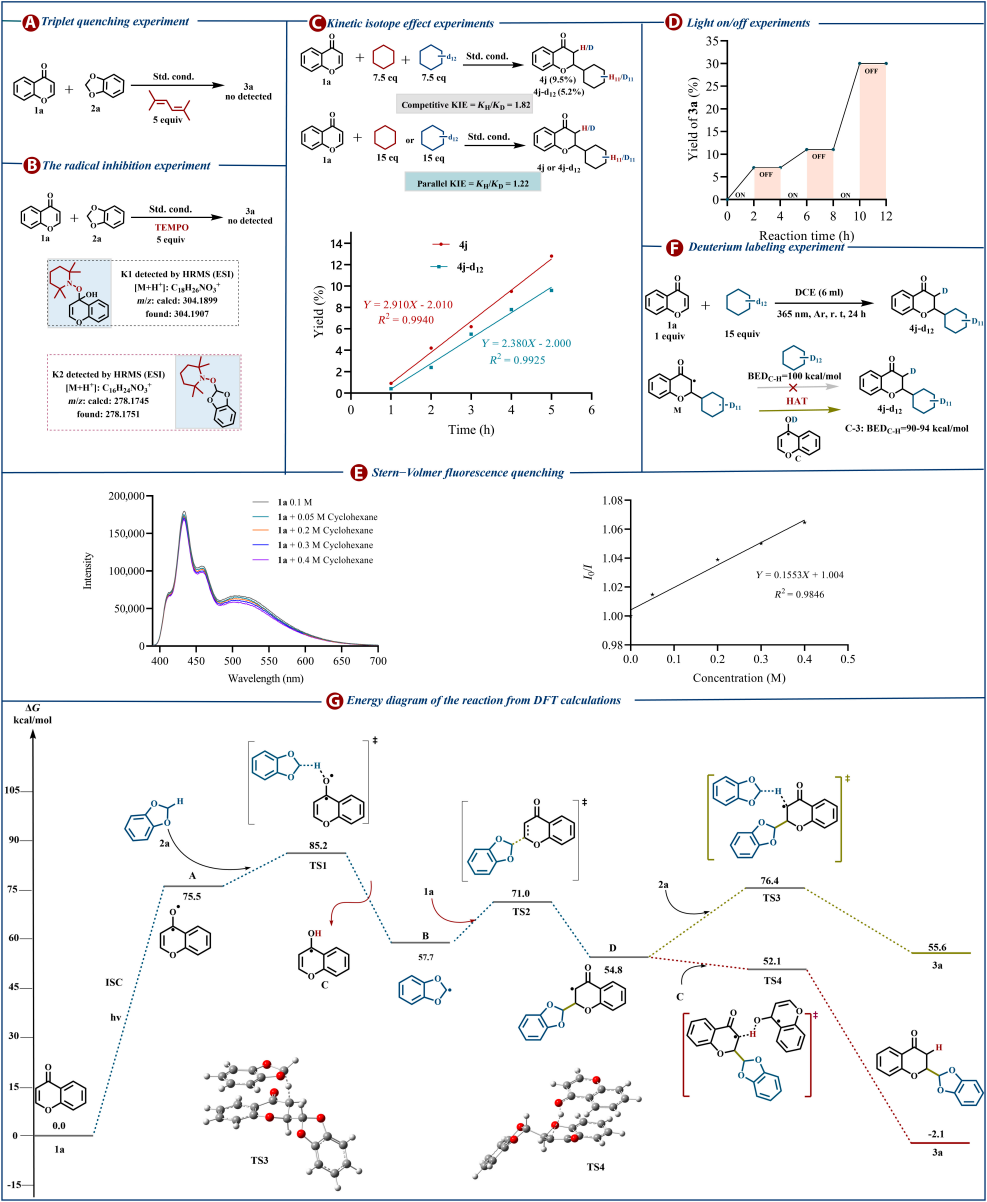
(A) Triplet quenching experiment. (B) The radical inhibition experiment. (C) Kinetic isotope effect experiment. (D) Light on/off experiment. (E) Stern−Volmer fluorescence quenching. (F) Deuterium labeling experiment. (G) Energy diagram of the reaction from DFT calculations.

To elucidate the intrinsic reaction mechanism, a systematic set of mechanistic investigations was performed. Time-correlated single photon counting (TCSPC) measurements showed that the fluorescence lifetime of the singlet excited state of chromone was 3.2 ns alone and 2.8 ns in the presence of benzodioxole. This indicates that the singlet state is not markedly quenched and suggests that substrate activation may proceed via the triplet excited state of chromone (Figs. S2 and S3). The addition of 1,1,4,4-tetramethylbutadiene, a selective triplet quencher, markedly suppressed the reaction, indicating the involvement of a triplet excited state [75] (Fig. 5A). The addition of 2,2,6,6-tetramethyl-1-piperidinyloxy (TEMPO) as a radical scavenger fully suppressed the formation of **3a**, and high-resolution mass spectrometer (HRMS) analysis confirmed the formation of TEMPO–radical adducts **K1** and **K2** (Fig. 5B), supporting a radical-based pathway. Kinetic isotope effect (KIE) studies using cyclohexane and cyclohexane-d_12_ in competitive and parallel experiments gave values of 1.82 and 1.22, respectively, indicating that the reaction rate is not governed by C(sp^3^)–H bond cleavage (Fig. 5C). Intermittent irradiation (photo-switching) experiments demonstrated that product formation ceased in the dark and resumed upon re-illumination, confirming the necessity of continuous photoexcitation (Fig. 5D). In addition, the moderate quantum yield (Φ = 0.384) suggests that an extended radical chain pathway is unlikely. Stern–Volmer fluorescence measurements show a slight concentration-dependent change in the excited-state emission intensity of chromone (**1a**) upon addition of cyclohexane (Fig. 5E), indicating that fast substrate-induced excited-state deactivation is not dominant. Furthermore, deuterium-labeling experiments using cyclohexane-d₁₂ afforded product **4j**-d_12_ [25,76]. Given that the C–H bond at the 3-position of **4j**-d_12_ (BDE = 90 to 94 kcal/mol) [77] is weaker than that of cyclohexane (BDE =100 kcal/mol), deuterium incorporation is proposed to occur via HAT from an intermediate species **C**, consistent with BDE considerations [78] (Fig. 5F).

To provide more robust support for the proposed catalytic cycle, we conducted density functional theory (DFT) calculations encompassing all intermediates and transition states associated with the reaction of chromone (**1a**) and benzodioxole (**2a**) (Fig. 5G). Excitation of chromone at 365 nm generates the triplet state **A** (75.5 kcal/mol above the ground state). Hydrogen-atom transfer from **2a** to **A** proceeds through transition state **TS1** (Δ*G*^‡^ = 9.7 kcal/mol) and furnishes radical pair **B**/**C**. **B** adds to a ground-state chromone in a Giese reaction via **TS2** (Δ*G*^‡^ =13.3 kcal/mol) to deliver intermediate **D**. **D** then acquires a hydrogen atom by 1 of 2 routes: direct HAT from **2a** (**TS3**, Δ*G*^‡^ =21.6 kcal/mol) or transfer from radical **C** (**TS4**, Δ*G*^‡^ =-2.7 kcal/mol). The markedly lower barrier associated with **TS4** demonstrates that hydrogen transfer from **C** to **D** is both kinetically and thermodynamically favored (exergonic by 2.7 kcal/mol), giving rise to product **3a** along with the regeneration of chromone. These computational results are consistent with the deuterium-labeling experiments and strongly support the proposed mechanism.

**Fig. 6.**
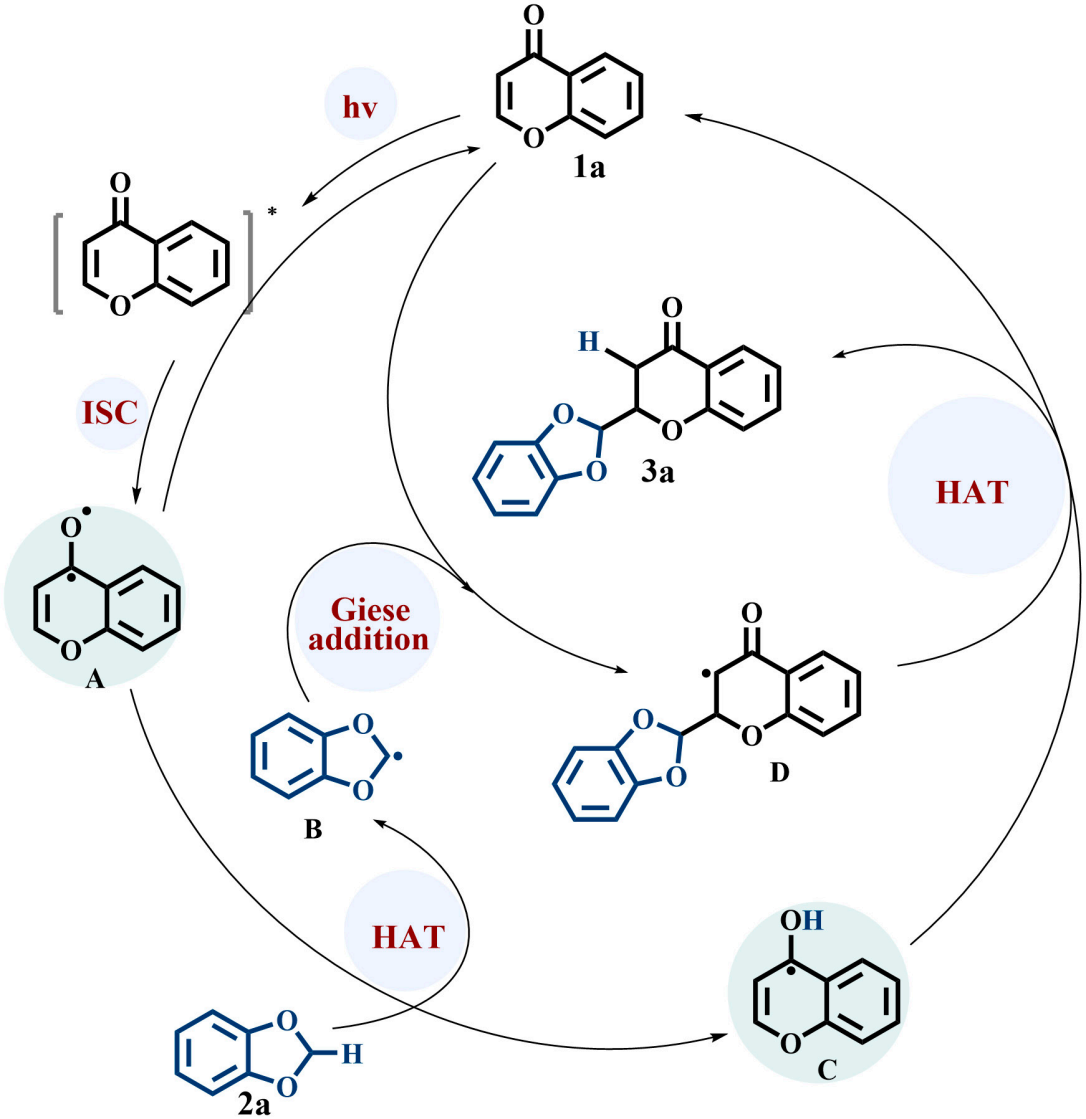
Proposed reaction mechanism.

Based on the results of mechanistic interrogations, a plausible reaction mechanism is proposed (Fig. 6). Under 365 nm irradiation, chromone (**1a**) undergoes photoexcitation and intersystem crossing to form the triplet diradical species **A**. A hydrogen atom is then transferred from **2a** to **A** through transition state **TS1**, with an activation energy of 9.7 kcal/mol, generating radicals **B** and **C**. Radical **B** subsequently reacts with a ground-state chromone molecule via **TS2** (Δ*G*^‡^ = 13.3 kcal/mol) to form intermediate **D**. In the final step, **D** abstracts a hydrogen atom from radical **C**, producing the product **3a** and regenerating the chromone catalyst, thus completing the catalytic cycle.

The mechanism pinpoints a dual duty for photoexcited chromone: the molecule is simultaneously the substrate and the HAT agent. Earlier radical-addition protocols operate through thermal initiation or metal-mediated channels; in those setups, radicals form exclusively to execute HAT and never engage the substrate directly. Our photocatalytic platform breaks with that tradition. It harnesses the excited-state chromone itself. This built-in reactivity opens normally unreactive C–H and P–H bonds under mild conditions, streamlines the operational sequence, and removes the usual requirements for metal catalysts or external radical initiators.

## Conclusion

Herein, we report a photocatalytic Giese addition protocol wherein chromone derivatives serve dually as substrates and HAT mediators. This transformation occurs under mild conditions and does not require external photocatalysts, metal additives, stoichiometric oxidants, or additional HAT reagents, in line with green chemistry principles. This method is compatible with a wide variety of substrates, allowing efficient activation of inert C–H bonds in ethers, esters, alcohols, alkanes, amines, sulfides, and P–H bonds in arylphosphines. These 2-substituted 4-chromanones are valuable intermediates. They allow various post-synthetic modifications and illustrate the practicality of this strategy. Mechanistic investigations (comprising control experiments, spectroscopic analyses, and DFT calculations) collectively support a photoinduced, chromone-mediated HAT catalytic cycle. Overall, this work establishes an efficient, metal-free, and sustainable route to 2-substituted 4-chromanone derivatives through direct photocatalytic HAT activation.

## Materials and Methods

All reagents and solvents of reagent grade were obtained from commercial suppliers and used as received without additional purification. Nuclear magnetic resonance spectra were recorded in CDCl₃ on JEOL JNM-ECZ600R/M1 (^1^H 600 MHz, ^13^C 15 MHz), JEOL-ECX-500 (^1^H 500 MHz, ^13^C 126 MHz, ^19^F 471 MHz, ^31^P 202 MHz), or Bruker AG-400 (^1^H 400 MHz, ^13^C 101 MHz) spectrometers. HRMS analyses were carried out on a Thermo Scientific Orbitrap with an ESI source. Thin-layer chromatography (TLC) was performed on 0.25-mm silica gel plates (Merck), and flash chromatography used 300 to 400 mesh silica gel with petroleum ether/ethyl acetate.

General procedure: Substrate **1** (0.2 mmol) and solid substrate **2** (3.0 mmol) were added in sequence to a 10-ml glass vial with a magnetic stir bar, which was then sealed. The vial was evacuated and refilled with argon 3 times using an oil pump. Anhydrous DCE (6.0 ml) was then added as the reaction solvent. For liquid substrates **2** (3.0 mmol) or alcohols (2.0 ml), the addition was performed after the completion of the argon purging process; stirring of the resulting mixture was performed under a 9-W, 365-nm LED light for 5 to 32 h. TLC was employed to track the reaction progress. Upon completion, the reaction mixture was concentrated under reduced pressure, and the target product was isolated via flash column chromatography (eluent: petroleum ether/ethyl acetate).

## Data Availability

All experimental data are available in the Supplementary Materials or from the corresponding authors upon request. The x-ray crystallographic coordinates for structures reported in this article have been deposited at the Cambridge Crystallographic Data Centre (CCDC), under deposition numbers CCDC 2456397 (3a), 2480146 (A1), 2480147 (B1), and 2473555 (D1). The data can be obtained free of charge from The Cambridge Crystallographic Data Centre via http://www.ccdc.cam.ac.uk/data_request/cif

## Supplementary Materials

Supplementary 1 Graphical Abstract Supplementary Text Tables S1 to S14 Schemes S1 to S10 Figs. S1 to S191
