# Supplementary material for: Chromones as Photocatalyzed HAT Reagent and Michael Acceptor for Direct C–H Alkylation of 2-Substituted 4-Chromanones
Source: Research (Wash D C). 2026 May 11;9:1250. doi: 10.34133/research.1250 (PMC13158460; doi:10.34133/research.1250)
Supplement: Supplementary 1 — Graphical Abstract Supplementary Text Tables S1 to S14 Schemes S1 to S10 Figs. S1 to S191 [file research.1250.f1.zip › XY-SI-R2.docx]

Supporting Information

**Chromones as Photocatalyzed HAT Reagent and Michael Acceptor for Direct C-H Alkylation of 2‑Substituted 4‑Chromanones**

Ya Xiao^†,a^, Junrong Zhang^†,a^, Wujun Jian^a^, Ya Zhou^a^, Xiang Zhou^a,*^, WMWW Kandegama^b^, Song Yang^a,*^

^a^ State Key Laboratory of Green Pesticides, Key Laboratory of Green Pesticide and Agricultural Bioengineering, Ministry of Education, Center for R&D of Fine Chemicals of Guizhou University, Guiyang 550025, China

^b^ Department of Horticulture and Landscape Gardening, Faculty of Agriculture and Plantation Management, Wayamba University of Sri Lanka, Makandura, Gonawila, Sri Lanka

^†^These authors contributed equally to this work.

* Corresponding author.

E-mail: jhzx.msm@gmail.com or syang@gzu.edu.cn (S. Yang)

zhoux1534@163.com or xiangzhou@gzu.edu.cn (X. Zhou)

**Contents**

[1 General experimental detail 3](#_Toc221740882)

[2 Reparation of Starting Materials 3](#_Toc221740883)

[3 Optimization of Reaction Conditions 4](#_Toc221740884)

[4 General procedure 8](#_Toc221740885)

[5 Mechanistic Investigations 9](#_Toc221740886)

[5.1 Time-Resolved Fluorescence Measurement of Chromone and Chromone–Benzodioxole Mixture Using TCSPC 9](#_Toc221740887)

[5.2 Triplet quenching experiment 11](#_Toc221740888)

[5.3 HRMS analysis of reaction mixture 11](#_Toc221740889)

[5.4 Kinetic isotope effect experiment 13](#_Toc221740890)

[5.5 Light on/off experiments 14](#_Toc221740891)

[5.6 Stern-Volmer fluorescence quenching 15](#_Toc221740892)

[5.7 Deuterium labelling experiment 17](#_Toc221740893)

[5.8 DFT calculations 19](#_Toc221740894)

[6 Scaled-up reaction 27](#_Toc221740895)

[7 Further transformations 27](#_Toc221740896)

[8 Measurement of quantum yield 29](#_Toc221740897)

[9 X-Ray crystal structure data 30](#_Toc221740898)

[10 Characterization data of compounds 35](#_Toc221740899)

[11 ^1^H NMR, ^13^C NMR, ^19^F NMR, ^31^P NMR, HRMS spectra 57](#_Toc221740900)

[Reference 145](#_Toc221740901)

# 1 General experimental detail

Unless otherwise stated, all reagents and solvents were of reagent grade, purchased commercially, and used without further purification. NMR spectra were recorded in CDCl_3_ on a JEOL JNM-ECZ600R/M1 spectrometer (^1^H at 600 MHz, ^13^C at 150 MHz), a JEOL-ECX-500 spectrometer (^1^H at 500 MHz, ^13^C at 126 MHz, ^19^F at 471 MHz, ^31^P at 202 MHz), or a Bruker Biospin AG-400 spectrometer (^1^H at 400 MHz, ^13^C at 101 MHz). Chemical shifts (δ) are reported in parts per million (ppm) using tetramethylsilane (TMS) as the internal standard at room temperature. Signal multiplicities are abbreviated as s (singlet), d (doublet), dd (doublet of doublets), t (triplet), and m (multiplet). HRMS data were obtained on a Thermo Scientific Orbitrap mass spectrometer with an ESI source. Single crystals of compounds 3a and D1 suitable for X-ray diffraction were grown by slow evaporation of dichloromethane at room temperature and measured on a HyPix diffractometer at 293.15 K. TLC was performed on glass-backed silica gel plates (0.25 mm, Merck). Flash chromatography was carried out on 300–400 mesh silica gel using petroleum ether/ethyl acetate as eluent.

# 2 Reparation of Starting Materials

**Scheme S1** Preparation of compound **M1**.

This procedure was adapted from the literature precedent.

To a solution of carboxylic acid **1** (1.0 equiv., 1 mmol) and amine **S1** (1.0 equiv.) in anhydrous DCM (25 mL) was added HATu (1.1 equiv.) and DIPEA (2.0 equiv.). The reaction mixture was stirred for 8 hours at 0  °C and monitored by TLC. Upon completion, the reaction was quenched with water and extracted with ethyl acetate. The combined organic layers were dried over anhydrous Na₂SO₄, filtered, and concentrated under reduced pressure. The crude product was purified by flash column chromatography (petroleum ether/ethyl acetate = 7:1) to afford compound **M1** as a yellow solid (87% yield).

**Scheme S2** Preparation compound **M2**.

To a solution of carboxylic acid **1** (1.0 equiv., 1 mmol) and ethanol (1.0 equiv.) in anhydrous DCM (25 mL) was added HATu (1.1 equiv.) and DIPEA (2.0 equiv.). The reaction mixture was stirred for 8 hours at 0 °C and monitored by TLC. Upon completion, the reaction was quenched with water and extracted with ethyl acetate. The combined organic layers were dried over anhydrous Na₂SO₄, filtered, and concentrated under reduced pressure. The crude product was purified by flash column chromatography (petroleum ether/ethyl acetate = 5:1) to afford compound **M2** as a viscous yellow liquid (81% yield).

# 3 Optimization of Reaction Conditions

**Table S1.** Screening of light sources for the reaction*^a^*.

| **Entry** | **Light** | **Yield of 3a (%)** |
| --- | --- | --- |
| 1 | 365 nm | 59 |
| 2 | 390 nm | 29 |
| 3 | 405 nm | 27 |
| 4 | 410 nm | 21 |
| 5 | 430 nm | 14 |

(a) Reaction conditions: **1a** (0.2 mmol, 1.0 equiv.) **2a** (2.0 mmol, 10 equiv.) in 2 mL DCE, the reaction was irradiated with the 9 W LED lamp at room temperature and in the atmosphere of argon for 12 h, yields were determined by ^1^H NMR spectra using 1,4-Dimethoxybenzene as an internal standard.

**Table S2.** Screening of reaction solvents*^a^*.

| **Entry** | **Solvent** | **Yield of 3a (%)** |
| --- | --- | --- |
| 1 | acetone | NR*^b^* |
| 2 | MeCN | 9 |
| 3 | EA | NR*^b^* |
| 4 | DCM | 54 |
| 5 | DCE | 59 |

(a) Reaction conditions: **1a** (0.2 mmol, 1.0 equiv.) **2a** (2.0 mmol, 10 equiv.) in 2 mL solvent, the reaction was irradiated with the 9 W 365 nm LED lamp at room temperature and in the atmosphere of argon for 12 h, yields were determined by ^1^H NMR spectra using 1,4-Dimethoxybenzene as an internal standard. (b) NR = No Reaction.


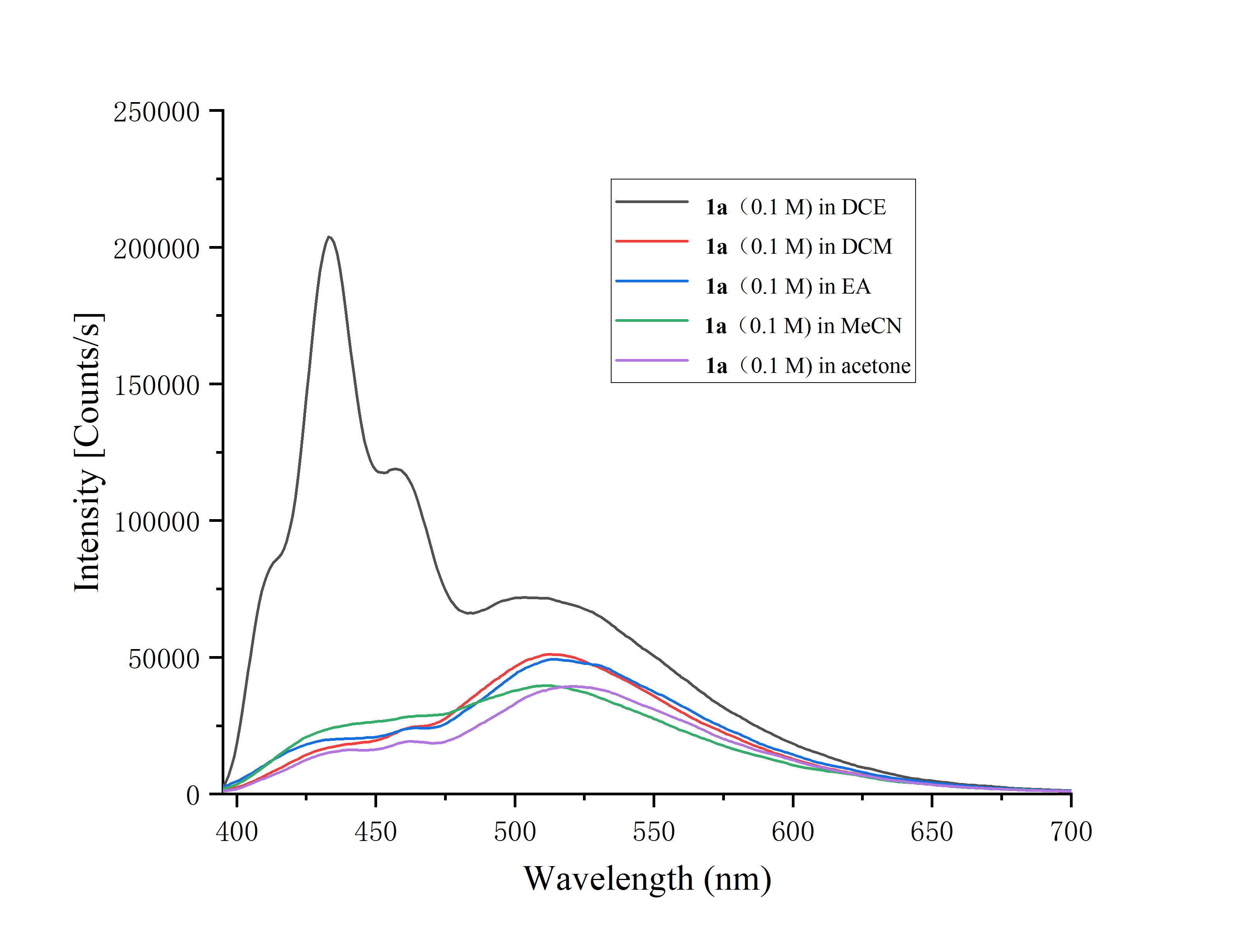
**Figure S1.** Fluorescence intensities of photoexcited chromone in different solvents.

**Table S3.** Screening of the amount of substrate **2a***^a^*.

| **Entry** | **2a (equiv)** | **Yield of 3a (%)** |
| --- | --- | --- |
| 1 | 2 | 46 |
| 2 | 3 | 59 |
| 3 | 4 | 58 |
| 4 | 5 | 61 |
| 5 | 6 | 60 |
| 6 | 7 | 65 |
| 7 | 8 | 65 |
| 8 | 9 | 61 |
| 9 | 10 | 61 |
| 10 | 12 | 62 |
| 11 | 14 | 65 |
| 12 | 15 | 67 |
| 13 | 16 | 66 |
| 14 | 18 | 62 |
| 15 | 20 | 63 |

(a) Reaction conditions: **1a** (0.2 mmol, 1.0 equiv.) **2a** in 2 mL DCE, the reaction was irradiated with the 9 W 365 nm LED lamp at room temperature and in the atmosphere of argon for 12 h, and yields were determined by ^1^H NMR spectra using 1,4-Dimethoxybenzene as an internal standard.

**Table S4.** Screening of solvent volume (DCE)*^a^*.

| **Entry** | **DCE (mL)** | **Yield of 3a (%)** |
| --- | --- | --- |
| 1 | 0.5 | 63 |
| 2 | 1.0 | 56 |
| 3 | 2.0 | 65 |
| 4 | 3.0 | 66 |
| 5 | 4.0 | 68 |
| 6 | 5.0 | 71 |
| 7 | 6.0 | 73 |
| 8 | 7.0 | 63 |
| 9 | 8.0 | 56 |

(a) Reaction conditions: **1a** (0.2 mmol, 1.0 equiv.) **2a** (3.0 mmol, 15 equiv.) in DCE, the reaction was irradiated with the 9 W 365 nm LED lamp at room temperature and in the atmosphere of argon for 12 h, yields were determined by ^1^H NMR spectra using 1,4-dimethoxybenzene as an internal standard.

**Table S5.** Screening of reaction time*^a^*.

| **Entry** | **Time (h)** | **Yield of 3a (%)** |
| --- | --- | --- |
| 1 | 4 | 40 |
| 2 | 6 | 48 |
| 3 | 8 | 54 |
| 4 | 10 | 64 |
| 5 | 12 | 73 |
| 6 | 14 | 70 |
| 7 | 16 | 63 |
| 8 | 18 | 67 |
| 9 | 20 | 54 |
| 10 | 22 | 55 |
| 11 | 24 | 56 |

(a) Reaction conditions: **1a** (0.2 mmol, 1.0 equiv.) **2a** (3.0 mmol, 15 equiv.) in 6 mL DCE, the reaction was irradiated with the 9 W 365 nm LED lamp at room temperature and in the atmosphere of argon yields were determined by ^1^H NMR spectra using 1,4-Dimethoxybenzene as an internal standard.

**Table S6.** Screening of temperature *^a^*.

| **Entry** | **Temperature/℃** | **Yield of 3a (%)** |
| --- | --- | --- |
| 1 | 0.0 | 59 |
| 2 | 10.0 | 62 |
| 3 | 20.0 | 66 |
| 4 | 25.0 | 72 |
| 5 | 30.0 | 69 |
| 6 | 40.0 | 53 |

(a) Reaction conditions: **1a** (0.2 mmol, 1.0 equiv.) **2a** (3.0 mmol, 15 equiv.) in DCE, the reaction was irradiated with the 9 W 365 nm LED lamp in the atmosphere of argon for 12 h, yields were determined by ^1^H NMR spectra using 1,4-dimethoxybenzene as an internal standard, 25 ℃ corresponds to room temperature.

**Table S7.** Screening of LED light source power *^a^*.

| **Entry** | **LED light source power (w)** | **Yield of 3a (%)** |
| --- | --- | --- |
| 1 | 3 | 43 |
| 2 | 4 | 52 |
| 3 | 5 | 56 |
| 4 | 6 | 58 |
| 5 | 7 | 61 |
| 6 | 8 | 65 |
| 7 | 9 | 71 |
| 8 | 10 | 63 |

(a) Reaction conditions: **1a** (0.2 mmol, 1.0 equiv.) **2a** (3.0 mmol, 15 equiv.) in 6 mL DCE, the reaction was irradiated with the 365 nm LED lamp at room temperature and in the atmosphere of argon yields were determined by ^1^H NMR spectra using 1,4-Dimethoxybenzene as an internal standard.

**Table S8.** Screening of Various boron-based additives and equivalents*^a^*.

| **Entry** | **Additives** | **equiv** | **Yield of 3v (%)** |
| --- | --- | --- | --- |
| 1 | / | / | 30 |
| 2 | Phenylboronic acid | 0.5 | 47 |
| 3 | 4-Bromophenylboronic acid | 0.5 | 9 |
| 4 | 4-Methylphenylboronic acid | 0.5 | 48 |
| 5 | Boric acid | 0.5 | 51 |
| 6 | Boric acid | 1.0 | 55 |
| 7 | Boric acid | 1.5 | 56 |
| 8 | Boric acid | 2.0 | 51 |

(a) Reaction conditions: **1a** (0.2 mmol, 1.0 equiv.) in 2 mL CH_3_OH, the reaction was irradiated with the 9 W 365 nm LED lamp at room temperature and in the atmosphere of argon for 24 h, yields were determined by ^1^H NMR spectra using 1,4-Dimethoxybenzene as an internal standard.

# 4 General procedure

Substrate **1** (0.2 mmol, 1.0 equiv.) and solid substrate **2** (3.0 mmol, 15 equiv.) were sequentially added to a sealed 10 mL glass vial equipped with a magnetic stirring bar. The vial was evacuated and backfilled with argon three times using an oil pump. Anhydrous DCE (6.0 mL) was then added as the reaction solvent. For liquid substrates **2** (3.0 mmol, 15 equiv.) or alcohols (2.0 mL), the addition was performed after the completion of the argon purging process. The resulting mixture was stirred under the irradiation of a 9 W, 365 nm LED lamp for 5-32 h. The progress of the reaction was monitored by thin-layer chromatography (TLC). Upon completion, the reaction mixture was concentrated under reduced pressure, and the crude product was purified by flash column chromatography (petroleum ether/ethyl acetate as the eluent) to afford the desired product.


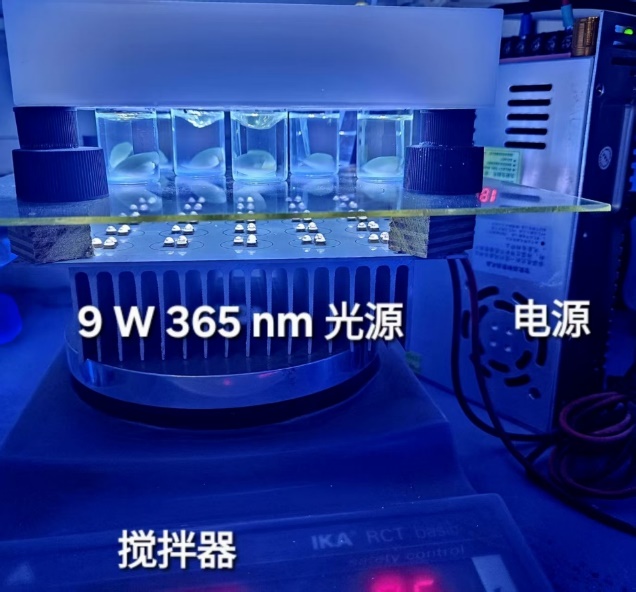


**Figure S2.** Reaction setup.

# 5 Mechanistic Investigations

# 5.1 Time-Resolved Fluorescence Measurement of Chromone and Chromone–Benzodioxole Mixture Using TCSPC


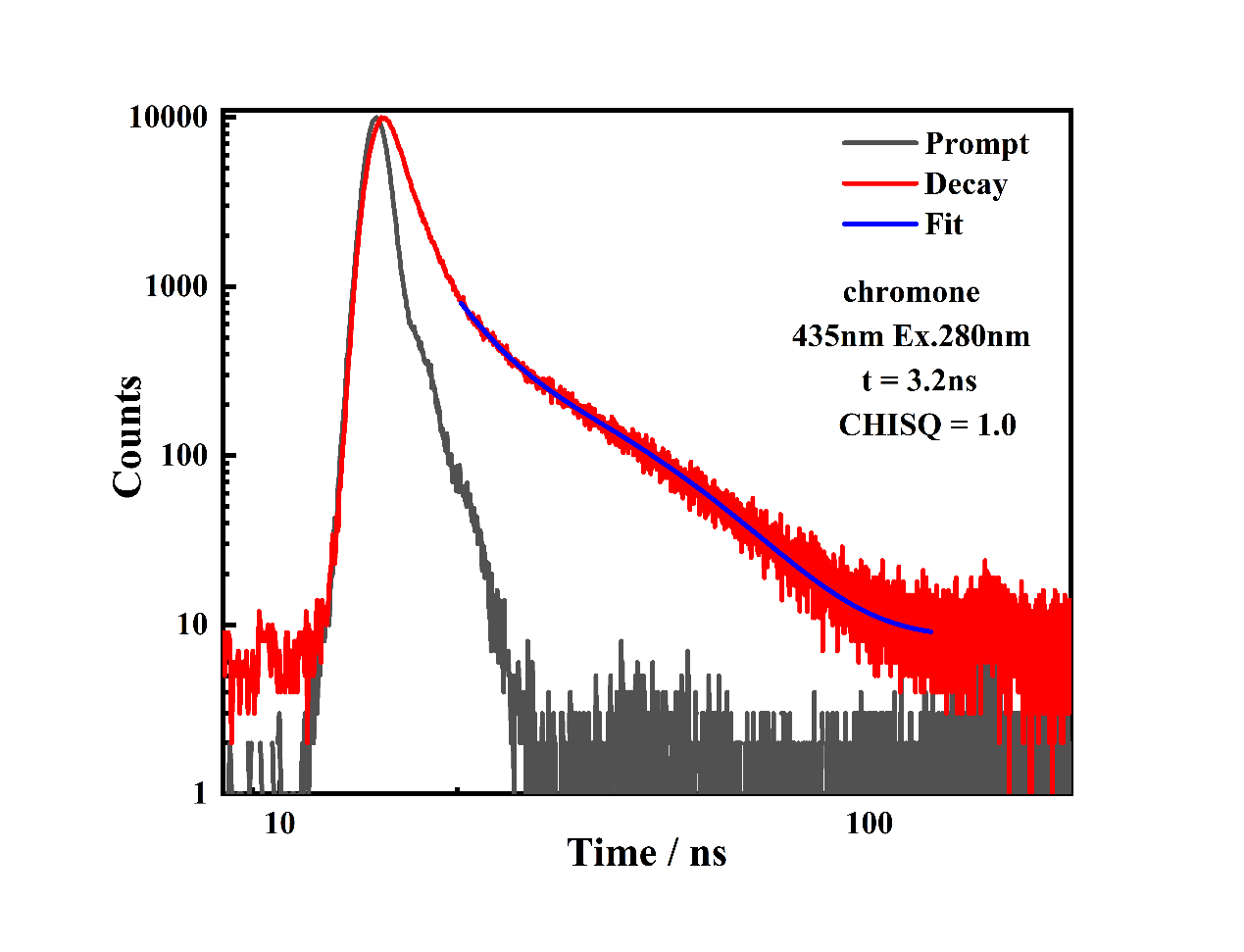


**Figure S3.** TCSPC Analysis of the Emission Decay of Chromone


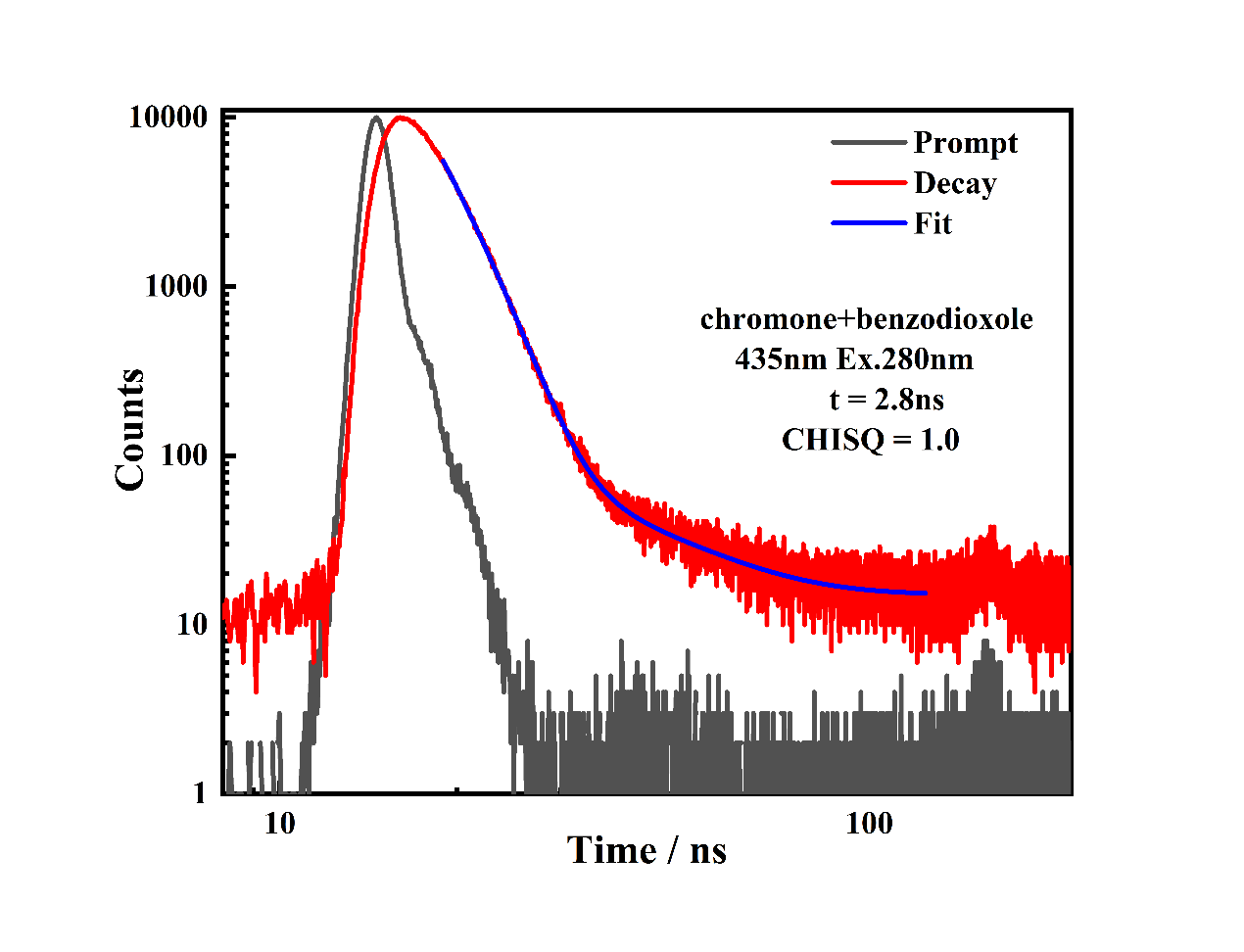


**Figure S4.** TCSPC Analysis of the Emission Decay of Chromone–Benzodioxole

**Table S9.** Data of TCSPC analysis.

|  | T_1_ (ns) | T_2_ (ns) | B_1_ (%) | B_2_ (%) | τₐᵥg(ns) | Chi² |
| --- | --- | --- | --- | --- | --- | --- |
| Chromone | 2.97 | 17.13 | 91.12 | 8.88 | 3.20 | 1.042 |
| Chromone–Benzodioxole | 2.78 | 18.04 | 0.02 | 99.98 | 2.78 | 1.048 |

The T₁/T₂ values are ordered according to their lifetimes, with the shorter lifetime listed first. B₁/B₂ represent the relative amplitude percentages of the corresponding components. For the chromone–Benzodioxole system, the minor component B₁ is very small (<1%) and can be considered negligible. The Chi² values provide a measure of the fitting quality and fall within an acceptable range.

# 5.2 Triplet quenching experiment

**Scheme S3.** Triplet quenching experiment. Reaction conditions: **1a** (0.2 mmol, 1.0 equiv.) **2a** (3.0 mmol, 15 equiv.) 1,1,4,4-tetramethylbutadiene (5 equiv.) in 6 mL DCE, the reaction was irradiated with the 9 W 365 nm LED lamp at room temperature and in the atmosphere of argon for 12 h.

# 5.3 HRMS analysis of reaction mixture

**Scheme S4.** The radical inhibition experiment. Reaction conditions: **1a** (0.2 mmol, 1.0 equiv.), **2a** (3.0 mmol, 15 equiv.), and TEMPO (1.0 mmol, 5.0 equiv.) were added to a 10 mL glass vial equipped with a magnetic stir bar. After purging with argon, anhydrous DCE (6.0 mL) was added as the solvent. The mixture was stirred under irradiation with a 9 W 365 nm LED lamp at room temperature under an argon atmosphere for 12 hours.

Upon completion, the reaction mixture was analyzed by high-resolution mass spectrometry (HRMS, ESI). The desired product **3a** was not detected; instead, two TEMPO-trapped adducts, **K1** and **K2**, were observed.

**Figure S5.** HRMS spectrums of **K1**.

**Figure S6.** HRMS spectrums of **K2**.

# 5.4 Kinetic isotope effect experiment

**Scheme S5.** The competitive kinetic isotope effect experiment. Reaction conditions: **1a** (0.2 mmol, 1.0 equiv.), cyclohexane (1.5 mmol, 7.5 equiv.), and cyclohexane-d_12_ (1.5 mmol, 7.5 equiv.) were added to a 10 mL glass vial equipped with a magnetic stir bar. After purging with argon, Anhydrous DCE (6.0 mL) was added as the solvent. The reaction mixture was stirred under irradiation with a 9 W 365 nm LED lamp at room temperature under an argon atmosphere for 5 h. The reaction yield was determined by proton nuclear magnetic resonance (¹H NMR) analysis using 1,4-dimethoxybenzene as an internal standard.

**Scheme S6.** The competitive kinetic isotope effect experiment. Reaction conditions: Cyclohexane (3.0 mmol, 15 equiv.) or cyclohexane-d_12_ (3.0 mmol, 15 equiv.) with substrate **1a** (0.2 mmol, 1.0 equiv.) in separate oven-dried 10 mL glass vials equipped with magnetic stir bars. After purging with argon, anhydrous DCE (6.0 mL) was added as the solvent. The reactions were irradiated with a 9 W 365 nm LED lamp under an argon atmosphere at room temperature. At designated time points (every 1 h), reaction aliquots were withdrawn. After concentration under reduced pressure, the crude mixtures were analyzed by ^1^H NMR spectroscopy using 1,4-dimethoxybenzene as the internal standard to determine the product yields. The kinetic isotope effect (K_H_/K_D_) was calculated by comparing the initial rates of the two reactions.

**Table S10.** Yield at the end of each 1 h.

| **Time(h)** | **Yield of 4j (%)** | **Yield of4j-d_12_ (%)** |
| --- | --- | --- |
| 1 | 0.9 | 0.4 |
| 2 | 4.2 | 2.4 |
| 3 | 6.2 | 5.5 |
| 4 | 9.5 | 7.8 |
| 5 | 12.8 | 9.6 |

**Figure S7.** The parallel kinetic isotope effect experiments.

# 5.5 Light on/off experiments

**Scheme S7.** Light on/off experiments. Reaction conditions: Six parallel reactions were carried out using **1a** (0.2 mmol, 1.0 equiv.) and **2a** (3.0 mmol, 15 equiv.) in 6 mL of DCE. The mixtures were irradiated with a 9 W 365 nm LED lamp at room temperature under an argon atmosphere according to the procedure. At specified time intervals, the yield of product **3a** was determined by ^1^H NMR spectroscopy using 1,2-dimethoxybenzene as an internal standard. The light irradiation was represented by the white area, while the red areas represent the intervals kept in darkness.


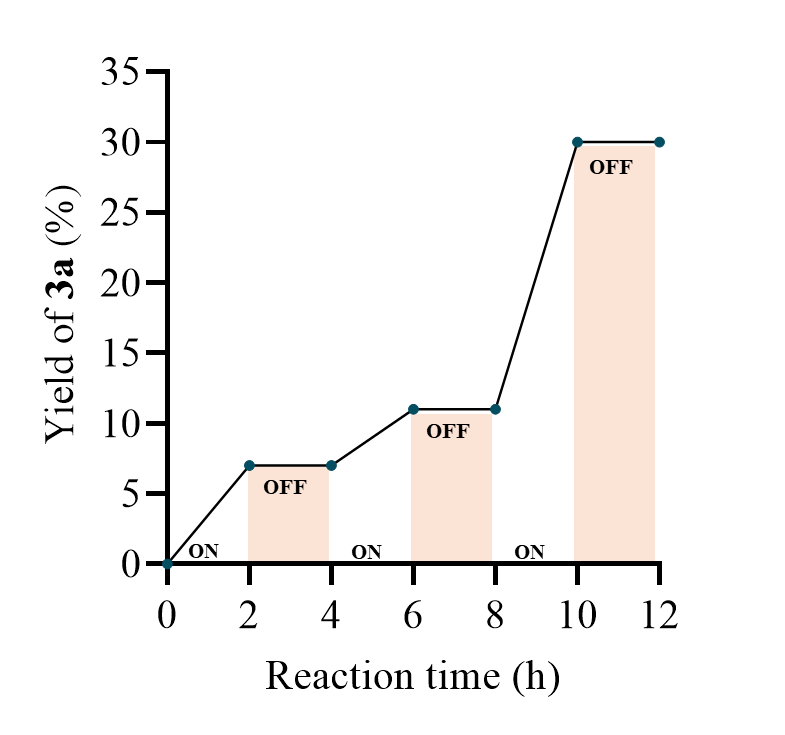


**Figure S8.** Light on/off experiments of **3a**.

# 5.6 Stern-Volmer fluorescence quenching

The Stern-Volmer fluorescence quenching experiments were performed at room temperature using a FluoroMax®-4P spectrometer (HORIBA Scientific, Paris, France). The excitation and emission wavelengths were set at 365 nm and 400 nm, respectively, with a slit width of 5 nm. Each sample was measured in triplicate. A solution of **1a** (0.1 mol/L) in anhydrous DCE was used as the blank, and its initial fluorescence intensity was recorded as I₀. The quenching effect of varying volumes of cyclohexane on the fluorescence intensity of 1a was then investigated.

**Figure S9.** Emission spectra of **1a** (0.1 mol/L) at different concentrations of cyclohexane.

**Figure S10.** Steady-state Stern-Volmer plot of **1a** (0.1 mol/L) at different concentrations of cyclohexane.

# 5.7 Deuterium labelling experiment

**Scheme S8.** Deuterium labelling experiment. Reaction conditions: **1a** (0.2 mmol, 1.0 equiv.), cyclohexane-d_12_ (3.0 mmol, 15 equiv.) in separate oven-dried 10 mL glass vials equipped with magnetic stir bars. After purging with argon, Anhydrous DCE (6.0 mL) was added as the solvent. The reactions were irradiated with a 9 W 365 nm LED lamp under an argon atmosphere at room temperature for 24 h.

**2-(cyclohexyl-d_11_)chroman-4-one-3-d (4j-d_12_).** Yellow liquid, yield 21%; ^1^H NMR (500 MHz, CDCl_3_) δ 7.78 (dd, *J* = 7.8, 1.8 Hz, 1H), 7.38 (ddd, *J* = 8.7, 7.2, 1.8 Hz, 1H), 6.93 – 6.86 (m, 2H), 4.17 – 4.05 (m, 1H), 2.67 – 2.54 (m, 1H). ^13^C NMR (126 MHz, CDCl_3_) δ 193.41, 162.01, 136.06, 127.00, 121.14, 121.09, 118.01, 82.07, 41.84, 40.32, 28.34, 28.26, 26.40, 26.04, 25.98.

HRMS (ESI) [M+H]^+^ calcd for C_15_H_7_D_12_O_2_: 243.2130, found: 243.2133.

**Figure S11.** ^1^H NMR Spectrum (CDCl_3_, 500 MHz) of **4j-d_12_**.

**Figure S12.** ^13^C NMR Spectrum (CDCl_3_, 126 MHz) of **4j-d_12_**.

# 5.8 DFT calculations

Computational details

All DFT computations were carried out with the Gaussian 16 (Revision A.03) program ^[1]^. Geometry optimizations and frequency calculations were performed using the M06-2X density functional ^[2,3]^. The 6-31G(d,p) ^[4,5]^ basis set was used for all atoms. Frequency analysis was conducted at the same level of theory at 298.15 K to confirm the stationary points as minima with zero imaginary frequencies and every transition state with one single imaginary frequency. Solution-phase single-point calculations were conducted using the SMD ^[6]^ solvation model in dichloroethane, M06-2X functional with def2-TZVP ^[7,8]^ basis set was used for all atoms.

Result and discussion


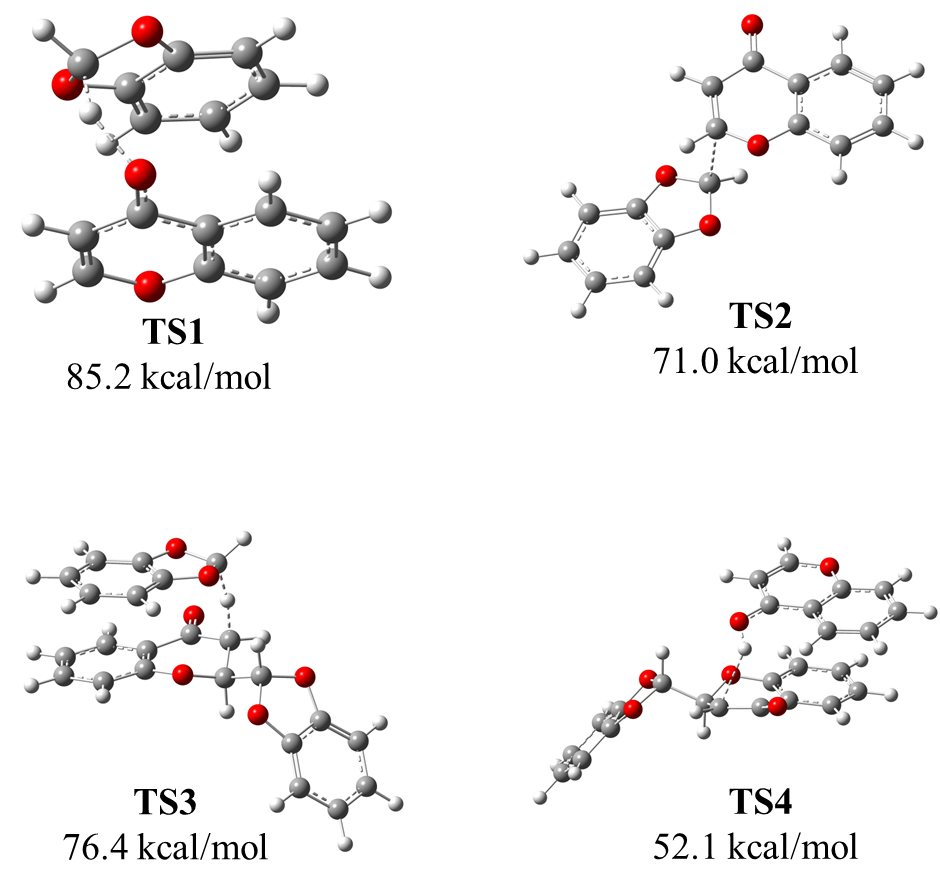


**Figure S13.** Energy of the transition state.

**Figure S14.** Energy diagram of the reaction from DFT calculations.

Cartesian coordinates in this work

**A**

C 1.91501400 -1.51178600 -0.26809000

C -0.33750800 -0.85621700 0.04986600

C -0.03566400 0.50687700 -0.02273700

C 1.38695300 0.94414000 0.02714800

C 2.34448200 -0.09126300 -0.04703100

H -1.84309000 -2.36978500 0.13524100

H 2.59883000 -2.32106700 -0.04222100

C -1.65096600 -1.30492000 0.07084800

C -1.07899600 1.42595100 -0.08176500

H 3.39338300 0.14813200 0.08002300

C -2.40096300 0.99219600 -0.07499600

C -2.68142100 -0.37083100 0.00493900

H -0.82188200 2.47943400 -0.12271600

H -3.21036900 1.71205500 -0.12398600

H -3.71059400 -0.71410200 0.01601900

O 0.64930800 -1.81494300 0.13766000

O 1.70420800 2.14749900 0.12590900

Thermal correction to Gibbs Free Energy = 0.090554

**B**

C 0.00291500 -0.23799000 0.69447700

C 0.00291500 -0.23799000 -0.69447700

C 0.00157500 0.92835300 -1.43032200

C 0.00291500 2.12151300 -0.69694200

C 0.00291500 2.12151300 0.69694200

C 0.00157500 0.92835300 1.43032200

C -0.12332900 -2.32215000 0.00000000

H 0.00071900 0.91222000 -2.51317500

H 0.00135500 3.06660000 -1.22855200

H 0.00135500 3.06660000 1.22855200

H 0.00071900 0.91222000 2.51317500

H 0.47799900 -3.22836100 0.00000000

O 0.01056000 -1.53368000 1.13140600

O 0.01056000 -1.53368000 -1.13140600

Thermal correction to Gibbs Free Energy = 0.073842

**C**

C 1.85014100 -1.56712100 0.00000600

C -0.40764000 -0.88052900 -0.00000400

C -0.02427700 0.47838300 -0.00000300

C 1.37403500 0.78328500 -0.00000300

C 2.29027500 -0.27609500 0.00000900

H -1.98230600 -2.31159300 0.00000000

H 2.48113800 -2.44324000 0.00001700

C -1.74034100 -1.25475700 0.00000000

C -1.03712500 1.45039800 0.00000100

H 3.36077200 -0.09556200 0.00002500

C -2.37546600 1.07642600 0.00000400

C -2.73016600 -0.27084100 0.00000300

H -0.74735400 2.49493700 0.00000200

H -3.14609400 1.84001600 0.00000500

H -3.77471700 -0.56240200 0.00000500

O 0.52279000 -1.89265700 -0.00001100

O 1.71886300 2.10040300 -0.00000100

H 2.67872300 2.18096800 -0.00003800

Thermal correction to Gibbs Free Energy = 0.104611

**D**

C 3.00404700 0.60013800 0.19675600

C 2.85342700 -0.77857600 0.29037700

C 3.76317200 -1.65095700 -0.26249100

C 4.85717300 -1.07578400 -0.92800000

C 5.00782800 0.30333200 -1.01878200

C 4.07161500 1.18040900 -0.44832000

C 1.03951300 0.17147600 1.14917200

H 3.63437800 -2.72320900 -0.18016900

H 5.59764700 -1.72537700 -1.38126100

H 5.86367600 0.71505300 -1.54185200

H 4.17669700 2.25682200 -0.50785600

H 0.67211500 0.27117600 2.17327500

O 1.96436800 1.21163400 0.85715400

O 1.72393900 -1.06321700 1.01486600

C -0.11878800 0.24299700 0.14408500

C -0.76593400 1.58267700 0.13881900

C -2.29124900 -0.75955600 0.10593700

C -2.99794600 -1.96118600 0.06951300

C -2.93082000 0.44836800 -0.19249600

C -4.34252100 -1.94723900 -0.27333800

H -2.47572000 -2.87991400 0.31095300

C -4.28549700 0.44196300 -0.53426800

C -4.99330700 -0.74811600 -0.58105100

H -4.89075900 -2.88337800 -0.30496800

H -4.75211200 1.39753300 -0.75121000

H -6.04387000 -0.75146500 -0.84892000

O -0.98279300 -0.82738200 0.48597800

C -2.18023000 1.72458400 -0.09744300

O -2.71802500 2.82424300 -0.21574500

H 0.31968000 0.04507300 -0.85355200

H -0.14454400 2.46827400 0.19672800

Thermal correction to Gibbs Free Energy = 0.192125

**1a**

C 1.92980300 -1.42349500 -0.00000300

C -0.33497500 -0.85959800 0.00000400

C -0.03036800 0.50226500 0.00001000

C 1.38159300 0.95512300 0.00004200

C 2.34529100 -0.14729700 -0.00000500

H -1.84734400 -2.37825500 -0.00000700

H 2.59460100 -2.27937800 -0.00000700

C -1.65568200 -1.31131900 -0.00000300

C -1.08128000 1.42786500 0.00000300

H 3.40351800 0.08000000 -0.00003200

C -2.39469200 0.99623600 -0.00000200

C -2.67733200 -0.37751100 -0.00000700

H -0.81783900 2.48058700 0.00000600

H -3.20692500 1.71467300 -0.00000400

H -3.70797100 -0.71676600 -0.00001000

O 0.64097800 -1.81747800 0.00000000

O 1.69499900 2.13316800 -0.00002300

Thermal correction to Gibbs Free Energy = 0.096878

**2a**

C -0.01876100 -0.19858900 0.69590900

C -0.01876100 -0.19858900 -0.69590900

C -0.05027300 0.96741300 -1.42595000

C -0.07785600 2.16628100 -0.69477200

C -0.07785600 2.16628100 0.69477200

C -0.05027300 0.96741300 1.42595000

C 0.24612800 -2.28796400 0.00000000

H -0.05485900 0.95155700 -2.50905200

H -0.09727200 3.10906200 -1.23023800

H -0.09727200 3.10906200 1.23023800

H -0.05485900 0.95155700 2.50905200

H -0.41292200 -3.15735400 0.00000000

H 1.30327000 -2.58815400 0.00000000

O -0.01876100 -1.49182500 1.14857600

O -0.01876100 -1.49182500 -1.14857600

Thermal correction to Gibbs Free Energy = 0.087038

**3a**

C 2.98093900 0.55145200 0.20149200

C 2.79620700 -0.82635400 0.20861300

C 3.63783200 -1.67829400 -0.46955900

C 4.69810400 -1.08427100 -1.17267100

C 4.88272000 0.29330400 -1.17757300

C 4.01611300 1.14946200 -0.47906800

C 1.08571400 0.10428500 1.28614400

H 3.48310600 -2.75020000 -0.45367900

H 5.38412400 -1.71788400 -1.72375100

H 5.71051200 0.72070800 -1.73232900

H 4.14806300 2.22456000 -0.47114000

H 0.84086800 0.13367700 2.35247700

O 2.01117300 1.14121600 0.97561500

O 1.71230700 -1.13620100 0.98865500

C -0.16668000 0.28528900 0.43850400

C -0.80896800 1.64966900 0.63965100

C -2.28455100 -0.72782500 0.26841200

H -0.13935600 2.45483700 0.33485100

C -3.00852700 -1.92081400 0.24593600

C -2.83617700 0.44586100 -0.26257300

C -4.27417000 -1.93514100 -0.32046100

H -2.55543500 -2.81216700 0.66449500

C -4.11163800 0.40627000 -0.83789300

C -4.83122700 -0.77526900 -0.87234900

H -4.83433400 -2.86462700 -0.34103300

H -4.50980200 1.33349100 -1.23782800

H -5.81990700 -0.80379100 -1.31622800

O -1.04591200 -0.76066200 0.83113600

C -2.10551700 1.73203100 -0.14582700

O -2.52592100 2.77260900 -0.60351600

H -1.05084100 1.78309300 1.70322300

H 0.10878100 0.14466800 -0.61884700

Thermal correction to Gibbs Free Energy = 0.206754

**TS1**

C -0.52643400 2.61100400 -0.93401700

C -1.90636100 0.82815400 -0.22440600

C -1.05281100 0.56735300 0.87017500

C 0.12686100 1.38234100 1.03455700

C 0.33366400 2.42090700 0.11539500

H -3.66249700 0.30830400 -1.31179500

H -0.45541700 3.39423800 -1.67385900

C -3.04070400 0.06611000 -0.45685300

C -1.38557300 -0.49309800 1.72572500

H 1.20058900 3.06490300 0.20996200

C -2.52251500 -1.25970900 1.49545400

C -3.35088800 -0.98384900 0.40915000

H -0.72286800 -0.69994300 2.55925700

H -2.76271200 -2.07944200 2.16491200

H -4.23940700 -1.58006200 0.23146100

O -1.63412200 1.82778200 -1.12338300

O 0.98659400 1.09369000 2.02047800

H 2.39918300 0.96877300 1.32091300

C 3.04550600 0.34286600 0.59475000

H 4.11268100 0.53794000 0.70094400

O 2.74388900 -0.99977200 0.87408500

O 2.60870900 0.61741100 -0.71446500

C 1.73230400 -1.31271300 0.01665000

C 1.65740600 -0.32890300 -0.96879200

C 0.87205700 -2.39222500 0.04270300

C 0.72182600 -0.36531900 -1.98130800

C -0.09355100 -2.43281100 -0.96650000

H 0.92912900 -3.13970400 0.82406200

C -0.16645500 -1.44477300 -1.95321100

H 0.65979400 0.42276000 -2.72153300

H -0.81949800 -3.23799500 -0.96982400

H -0.94551400 -1.50467100 -2.70506300

Thermal correction to Gibbs Free Energy = 0.197637

**TS2**

C 2.95154400 0.55146700 0.48827200

C 2.80510100 -0.81217100 0.26682900

C 3.83982200 -1.60096800 -0.18954300

C 5.05636400 -0.94607700 -0.41798800

C 5.20368200 0.42256900 -0.19490900

C 4.14254300 1.21042100 0.26835100

C 0.85297400 0.01526600 0.82498300

H 3.71054700 -2.66299400 -0.35696100

H 5.90248200 -1.51984000 -0.77904700

H 6.16213200 0.89195900 -0.38606900

H 4.24232900 2.27382700 0.44688400

H 0.08127500 -0.02800800 1.58973100

O 1.75868800 1.04774600 0.94533600

O 1.52082600 -1.16791500 0.58816400

C -0.31461300 0.41342300 -1.03653900

C -0.88253300 1.64303900 -0.83608500

C -2.26747700 -0.73474300 -0.44956100

C -2.89750000 -1.96940200 -0.28349600

C -2.89857400 0.45688200 -0.08602600

C -4.17237000 -2.00222100 0.25710800

H -2.37264200 -2.87046200 -0.58010200

C -4.18697000 0.39823900 0.45648000

C -4.82247100 -0.81865000 0.63129400

H -4.66863900 -2.95789300 0.39137400

H -4.65500700 1.34017800 0.72391300

H -5.82086800 -0.85945300 1.05278600

O -1.01237600 -0.75532200 -0.98893400

C -2.22212700 1.76528500 -0.29791200

O -2.77127300 2.82809500 -0.03705500

H 0.61959700 0.28120600 -1.57472700

H -0.29249500 2.53650400 -0.99541500

Thermal correction to Gibbs Free Energy = 0.188707

**TS3**

C 4.02356900 -0.15261600 0.49370700

C 3.78872400 -0.56770100 -0.81270800

C 4.75369100 -0.47878400 -1.79002100

C 5.99332600 0.05437200 -1.40179800

C 6.22718400 0.46750200 -0.09557000

C 5.23300900 0.36863200 0.89136200

C 1.87899300 -0.72837600 0.31566000

H 4.55765900 -0.80763900 -2.80324900

H 6.78139500 0.14598900 -2.14101000

H 7.19481400 0.87756000 0.17141600

H 5.40062200 0.68435600 1.91388900

H 1.31575800 -1.59262000 0.67842300

O 2.89941600 -0.38222100 1.24838800

O 2.51748000 -1.06985100 -0.90839900

C 0.95958500 0.47382600 0.11524100

C 0.37466100 0.97519800 1.41026200

C -1.10079000 0.87845200 -0.97069500

H 1.09046500 1.19758500 2.19897700

C -1.84120600 0.72252000 -2.14149400

C -1.46147800 1.84545100 -0.02269400

C -2.94150900 1.53879600 -2.36205100

H -1.54006100 -0.04135900 -2.84941300

C -2.57335300 2.65624800 -0.26440500

C -3.31332200 2.51074000 -1.42782900

H -3.51749700 1.41670200 -3.27425200

H -2.83208200 3.38706800 0.49547800

H -4.17399700 3.14401200 -1.61347400

O -0.04938000 0.02891600 -0.78211500

C -0.72062400 1.94750600 1.26445400

O -1.04354000 2.73091300 2.14458900

H 1.54897500 1.26823500 -0.37748400

H -0.30666600 -0.14494900 1.92195400

C -1.14376200 -1.11237300 2.26684400

H -0.91675700 -1.43280600 3.28422800

O -1.02575500 -2.14700500 1.33690000

O -2.40808700 -0.54105500 2.15068200

C -2.06725100 -1.97518000 0.45821600

C -2.91398700 -0.99564600 0.95933200

C -2.32107500 -2.63176900 -0.72608200

C -4.06558900 -0.61108300 0.30791800

C -3.49316100 -2.25904000 -1.39717100

H -1.64698000 -3.38785000 -1.10901200

C -4.34260600 -1.27537400 -0.89306300

H -4.70352200 0.16937600 0.70345700

H -3.73862000 -2.74524500 -2.33494700

H -5.23276900 -1.00378600 -1.44981300

Thermal correction to Gibbs Free Energy = 0.298257

**TS4**

C -4.70769100 -0.48671600 0.47195400

C -4.50608100 0.82148300 0.04126600

C -5.49367400 1.53896600 -0.59418300

C -6.72188000 0.88539200 -0.78927400

C -6.92242500 -0.41984100 -0.35788200

C -5.90525700 -1.13941400 0.29130300

C -2.57171700 0.04308400 0.83130100

H -5.32282700 2.55656400 -0.92401300

H -7.52584000 1.41352600 -1.29021300

H -7.88088100 -0.89859000 -0.52607600

H -6.04568900 -2.15743300 0.63372800

H -2.03421900 0.27109100 1.75698500

O -3.57336900 -0.93836500 1.08992300

O -3.24279400 1.21999500 0.37992900

C -1.60526000 -0.47688800 -0.22447900

C -0.90877700 -1.71357600 0.24780000

C 0.41702600 0.30617400 -1.15072300

H -1.42454500 -2.37618500 0.93346600

C 1.09589500 1.34725800 -1.79075200

C 0.92479800 -0.99909700 -1.16736600

C 2.26976200 1.07303500 -2.48231300

H 0.68234900 2.34933900 -1.73612500

C 2.09568100 -1.25367100 -1.88252000

C 2.76629100 -0.23399700 -2.54549200

H 2.79445700 1.87951700 -2.98629100

H 2.48250500 -2.26772600 -1.84485000

H 3.67910400 -0.44245800 -3.09456200

O -0.69227900 0.62120000 -0.43105200

C 0.34614100 -2.02682200 -0.23052500

O 1.11908800 -2.95610600 0.17582100

H -2.17625100 -0.59957900 -1.16387500

H 0.79366400 -1.24901500 1.57319300

O 0.89792300 -0.36838100 2.02600100

C 1.81177100 0.41709200 1.55265500

C 3.06033200 -0.01206500 0.98876500

C 1.56800100 1.81036600 1.66502000

C 3.92826200 0.98356100 0.50624900

C 3.47105400 -1.36239700 0.91478600

C 2.46598900 2.67796800 1.14087100

H 0.63347400 2.15687800 2.08419000

C 5.17656700 0.67510700 -0.03614100

O 3.60671500 2.30373900 0.57403500

C 4.71027100 -1.66520200 0.39753000

H 2.77353800 -2.15432700 1.17823700

H 2.33936200 3.75340500 1.12582800

H 5.80535700 1.47874000 -0.40027300

C 5.56070000 -0.64969700 -0.07730000

H 5.02066100 -2.70165900 0.33262800

H 6.52882300 -0.90936100 -0.49252800

Thermal correction to Gibbs Free Energy = 0.324647

# 6 Scaled-up reaction


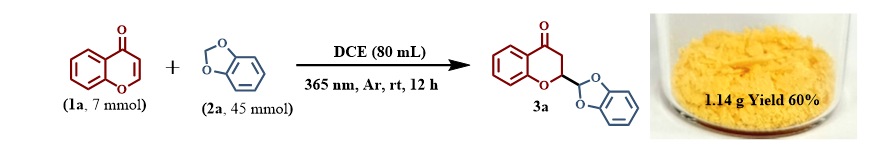


**Scheme S9.** Scaled-up reaction. Reaction conditions: **1a** (7 mmol, 1.0 equiv.) was added to a 150 mL oven-dried round-bottom flask. After sealing, the flask was purged with argon. Then, 80 mL of anhydrous 1,2-dichloroethane and **2a** (45 mmol, 6.3 equiv.) were added. The reaction mixture was irradiated with a 9 W 365 nm LED lamp at room temperature for 12 h. Upon completion, the reaction mixture was purified by column chromatography using petroleum ether/ethyl acetate as the eluent.

# 7 Further transformations


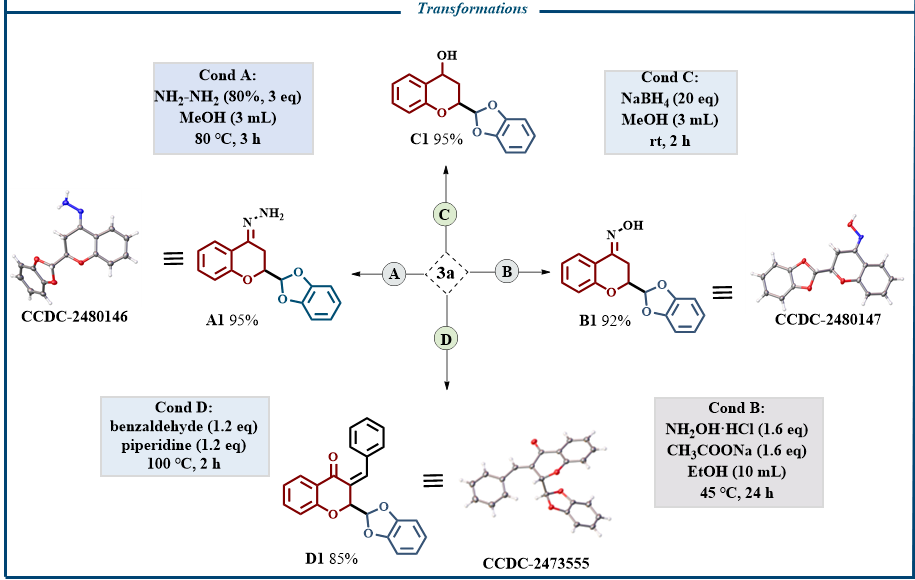


**Scheme S10.** Further transformations.

**General procedure for the synthesis of Compound A1^[9]^**.

Compound **3a** (1 mmol, 1 equiv.) and hydrazine hydrate (80%) (3 equiv.) were stirred in 3 mL of methanol at 80 °C for 3 h. After completion, the reaction mixture was cooled to room temperature and extracted with water and dichloromethane. The organic layers were combined, dried over anhydrous sodium sulfate, and concentrated under reduced pressure. The crude product was recrystallized from hexane/ethyl acetate (20:1) to afford a yellow solid in 92% yield.

**General procedure for the synthesis of Compound B1^[10]^**.

Compound **3a** (1 mmol, 1 equiv.), hydroxylamine hydrochloride (1.6 equiv.), and sodium acetate (1.6 equiv.) were stirred in 10 mL of ethanol at 40-50 °C for 24 h. After completion, the reaction mixture was cooled to room temperature, and water was added to precipitate the product. The resulting solid was purified by column chromatography using petroleum ether/ethyl acetate as the eluent to afford a white solid in 93% yield.

**General procedure for the synthesis of Compound C1^[11]^**.

Compound **3a** (0.5 mmol, 1 equiv.) and sodium borohydride (20 equiv.) were added to a 15 mL round-bottom flask. After sealing, the flask was purged with argon. Then, 3 mL of methanol was added, and the mixture was stirred at room temperature under an argon atmosphere (balloon) for 2 h. Upon completion, the product was purified by column chromatography using petroleum ether/ethyl acetate as the eluent to afford a yellow solid in 89% yield.

**General procedure for the synthesis of Compound D1^[12]^**.

Compound **3a** (0.5 mmol, 1 equiv.) and benzaldehyde (1.2 equiv.) were added to a 15 mL round-bottom flask, followed by the addition of 2 mL of piperidine. The reaction mixture was stirred at 100  °C for 2 h. Upon completion, the product was purified by column chromatography using petroleum ether/ethyl acetate as the eluent to afford a yellow solid in 86% yield.

# 8 Measurement of quantum yield

According to the procedure of Yoon^[13]^, the photon flux of the LED was determined by standard ferrioxalate actinometry. A 0.15 M solution of ferrioxalate was prepared by dissolving potassium ferrioxalate hydrate (2.21 g) in 30 mL of H_2_SO_4_ (0.05 M) solution. A buffered solution of 1,10-phenanthroline was prepared by dissolving 1,10-phenanthroline (50 mg) and sodium acetate (11.25 g) in 50 mL of H_2_SO_4_ (0.5 M) solution. Both solutions were stored in the dark. To determine the photon flux of the LEDs, the ferrioxalate solution (2.0 mL) was placed in a cuvette and irradiated for 40 s at λ = 365 nm. After irradiation, the phenanthroline solution (0.35 mL) was added to the cuvette, and the mixture was allowed to stir in the dark for 1 h to allow the ferrous ions to coordinate with phenanthroline completely. The absorbance of solution was measured at 510 nm. A non-irradiated sample was also prepared, and the absorbance at 510 nm was measured. Conversion was calculated using eq. 1.

$$n ({Fe}^{2+})=\frac{V\times\Delta A}{l\times\varepsilon} (1)$$

$$n \left( {Fe}^{2+} \right)=\frac{V\times\Delta A}{l\times\varepsilon}=\frac{0.00235 L\times1.7948}{1cm\times11100L {mol}^{-1} {cm}^{-1}}=3.7998\times{10}^{-7}\mathrm{mol}$$

Where *V* is the total volume (0.00235 L) of the solution after addition of phenanthroline, Δ*A* is the difference in absorbance at 510 nm between the irradiated and non-irradiated solutions, *l* is the path length (1 cm), and *ε* is the molar absorptivity at 510 nm (11100 L mol^–1^ cm^–1^)^[14]^. The photon flux can be calculated using eq. 2.

$$photon flux=\frac{n ({Fe}^{2+})}{\Phi\times t\times f} (2)$$

Where *Φ* is the quantum yield for the ferrioxalate actinometer (1.27 at λ = 365 nm)^[15]^, *t* is the time (40 s), the fraction of light absorbed *f* was calculated using eq. 3, where A(365 nm) is the absorbance of the ferrioxalate solution at 365 nm. The original solution was too concentrated for accurate measurement, so it was diluted 100-fold, and the measured absorbance (0.9093) was corrected for the dilution factor to obtain the absorbance of the original solution. The calculated fraction of light absorbed is *f* ≈ 1, indicating that nearly all light at 365 nm was absorbed^[16]^.

$$f=1-{10}^{-A \left( 365 \mathrm{nm} \right)}=1-{10}^{-90.93}\approx1 (3)$$

$$photon flux=\frac{n ({Fe}^{2+})}{\Phi\times t\times f}=\frac{3.7998\times{10}^{-7}\mathrm{mol}}{1.27\times40 s\times1}=7.4799{\times10}^{-9}\mathrm{einstein}s^{-1}$$

**1a** (0.2 mmol, 1.0 equiv.) and **2a** (3.0 mmol, 15 equiv.) were added to a 10 mL vial. The vial was purged with argon, and the solids were dissolved in 6 mL of DCE. Under an argon atmosphere, the reaction mixture was irradiated with a 9 W 365 nm LED at room temperature for 6 h. After completion, the yield of product **3a** was determined by ^1^H NMR spectroscopy using 1,2-dimethoxybenzene as an internal standard and was found to be 31% (6.2 x 10^-5^ mol). The reaction quantum yield (Φ) was determined using eq. 4.

$$Ф=\frac{Mol prodct}{photon flux\times t\times f}=\frac{6.2\times{10}^{-5}}{7.4799{\times10}^{-9}\times21600\times1}=0.384 (4)$$

# 9 X-Ray crystal structure data

Crystals of compounds **3a**, **A1**, **B1** and **D1** suitable for X-ray analysis were grown from the solvent of CH_2_Cl_2_ by the slow evaporation method. X-Ray Data Collection and Structure.

**Table S11** Crystal data of **3a**.

| Compound and CCDC number | **3a** (CCDC-2466397) |
| --- | --- |
| Empirical formula | C_16_H_12_O_4_ |
| Formula weight | 268.26 |
| Temperature/K | 100.00(10) |
| Crystal system | triclinic |
| Space group | P-1 |
| a/Å | 5.35050(10) |
| b/Å | 11.1255(2) |
| c/Å | 11.5283(2) |
| α/° | 64.407(2) |
| β/° | 88.495(2) |
| γ/° | 83.640(2) |
| Volume/Å^3^ | 614.93(2) |
| Z | 2 |
| ρ_calc_g/cm^3^ | 1.449 |
| μ/mm^‑1^ | 0.866 |
| F(000) | 280.0 |
| Crystal size/mm^3^ | 0.15 × 0.13 × 0.1 |
| Radiation | Cu Kα (λ = 1.54184) |
| 2Θ range for data collection/° | 16.054 to 149.456 |
| Index ranges | -6 ≤ h ≤ 6, -13 ≤ k ≤ 13, -10 ≤ l ≤ 14 |
| Reflections collected | 6148 |
| Independent reflections | 2329 [R_int_ = 0.0119, R_sigma_ = 0.0085] |
| Data/restraints/parameters | 2329/0/181 |
| Goodness-of-fit on F^2^ | 1.102 |
| Final R indexes [I>=2σ (I)] | R_1_ = 0.0387, wR_2_ = 0.0941 |
| Final R indexes [all data] | R_1_ = 0.0392, wR_2_ = 0.0944 |
| Largest diff. peak/hole / e Å^-3^ | 0.26/-0.21 |

**Table S12** Crystal data of **A1**.

| Compound and CCDC number | **A1** (CCDC-2480246) |
| --- | --- |
| Empirical formula | C_16_H_14_N_2_O_3_ |
| Formula weight | 282.29 |
| Temperature/K | 99.93(14) |
| Crystal system | monoclinic |
| Space group | P2_1_/c |
| a/Å | 20.2416(5) |
| b/Å | 4.82519(13) |
| c/Å | 13.5674(3) |
| α/° | 90 |
| β/° | 90.677(2) |
| γ/° | 90 |
| Volume/Å^3^ | 1325.03(6) |
| Z | 4 |
| ρ_calc_g/cm^3^ | 1.415 |
| μ/mm^‑1^ | 0.817 |
| F(000) | 592.0 |
| Crystal size/mm^3^ | 0.15 × 0.12 × 0.09 |
| Radiation | Cu Kα (λ = 1.54184) |
| 2Θ range for data collection/° | 4.366 to 149.03 |
| Index ranges | -24 ≤ h ≤ 25, -5 ≤ k ≤ 5, -13 ≤ l ≤ 16 |
| Reflections collected | 6450 |
| Independent reflections | 2591 [R_int_ = 0.0317, R_sigma_ = 0.0339] |
| Data/restraints/parameters | 2591/0/191 |
| Goodness-of-fit on F^2^ | 1.069 |
| Final R indexes [I>=2σ (I)] | R_1_ = 0.0451, wR_2_ = 0.1270 |
| Final R indexes [all data] | R_1_ = 0.0554, wR_2_ = 0.1313 |
| Largest diff. peak/hole / e Å^-3^ | 0.27/-0.23 |

**Table S13** Crystal data of **B1**.

| Compound and CCDC number | **B1** (CCDC-2480147) |
| --- | --- |
| Empirical formula | C16H13NO4 |
| Formula weight | 283.27 |
| Temperature/K | 99.99(10) |
| Crystal system | monoclinic |
| Space group | C2/c |
| a/Å | 26.6897(16) |
| b/Å | 4.2360(2) |
| c/Å | 24.6210(18) |
| α/° | 90 |
| β/° | 113.233(8) |
| γ/° | 90 |
| Volume/Å3 | 2557.9(3) |
| Z | 8 |
| ρcalcg/cm3 | 1.471 |
| μ/mm 1 | 0.887 |
| F(000) | 1184.0 |
| Crystal size/mm3 | 0.14 × 0.13 × 0.12 |
| Radiation | Cu Kα (λ = 1.54184) |
| 2Θ range for data collection/° | 7.208 to 145.634 |
| Index ranges | -28 ≤ h ≤ 32, -4 ≤ k ≤ 5, -30 ≤ l ≤ 27 |
| Reflections collected | 7093 |
| Independent reflections | 2421 [Rint = 0.0518, Rsigma = 0.0624] |
| Data/restraints/parameters | 2421/0/191 |
| Goodness-of-fit on F2 | 1.180 |
| Final R indexes [I>=2σ (I)] | R1 = 0.0914, wR2 = 0.2135 |
| Final R indexes [all data] | R1 = 0.1092, wR2 = 0.2248 |
| Largest diff. peak/hole / e Å-3 | 0.51/-0.40 |

**Table S14** Crystal data of **D1**

| Compound and CCDC number | **D1** (CCDC-2473555) |
| --- | --- |
| Empirical formula | C_23_H_16_O_4_ |
| Formula weight | 356.36 |
| Temperature/K | 100.00(10) |
| Crystal system | triclinic |
| Space group | P-1 |
| a/Å | 9.0609(5) |
| b/Å | 9.5793(5) |
| c/Å | 11.1694(3) |
| α/° | 102.505(4) |
| β/° | 103.125(4) |
| γ/° | 109.479(5) |
| Volume/Å^3^ | 843.81(7) |
| Z | 2 |
| ρ_calc_g/cm^3^ | 1.403 |
| μ/mm^‑1^ | 0.781 |
| F(000) | 372.0 |
| Crystal size/mm^3^ | 0.13 × 0.12 × 0.11 |
| Radiation | Cu Kα (λ = 1.54184) |
| 2Θ range for data collection/° | 8.574 to 149.478 |
| Index ranges | -10 ≤ h ≤ 11, -11 ≤ k ≤ 9, -13 ≤ l ≤ 13 |
| Reflections collected | 10907 |
| Independent reflections | 3229 [R_int_ = 0.0547, R_sigma_ = 0.0303] |
| Data/restraints/parameters | 3229/0/244 |
| Goodness-of-fit on F^2^ | 1.073 |
| Final R indexes [I>=2σ (I)] | R_1_ = 0.0759, wR_2_ = 0.2032 |
| Final R indexes [all data] | R_1_ = 0.0811, wR_2_ = 0.2102 |
| Largest diff. peak/hole / e Å^-3^ | 0.56/-0.37 |

# 10 Characterization data of compounds

**2-(benzo[*d*][1,3]dioxol-2-yl)chroman-4-one (3a).** A yellow solid, yield 66%, m. p. 97.2-98.7 ℃; ^1^H NMR (500 MHz, CDCl_3_) δ 7.88 (dd, *J* = 7.8, 1.8 Hz, 1H), 7.49 (m, 1H), 7.08 – 7.01 (m, 2H), 6.87 – 6.80 (m, 4H), 6.36 (d, *J* = 3.5 Hz, 1H), 4.74 (dt, *J* = 12.8, 3.4 Hz, 1H), 2.97 (dd, *J* = 16.8, 12.8 Hz, 1H), 2.82 (dd, *J* = 16.8, 3.4 Hz, 1H). ^13^C NMR (126 MHz, CDCl_3_) δ 190.56, 160.54, 147.10, 146.84, 136.42, 127.05, 122.18, 122.12, 121.09, 118.09, 108.89, 108.81, 108.63, 36.48. HRMS (ESI) [M+H]^+^ calcd for C_16_H_13_O_4_: 269.0801, found: 269.0808.

**2-(5-methylbenzo[*d*][1,3]dioxol-2-yl)chroman-4-one (3b).** A yellow solid, yield 24%; m. p. 74.7-75.3 ℃; ^1^H NMR (500 MHz, CDCl_3_) δ 7.91 (dd, *J* = 7.9, 1.7 Hz, 1H), 7.54 – 7.49 (m, 1H), 7.09 – 7.04 (m, 2H), 6.74 (dd, *J* = 7.9, 5.0 Hz, 1H), 6.71 – 6.64 (m, 2H), 6.37 (dd, *J* = 3.5, 0.7 Hz, 1H), 4.78 – 4.72 (m,, 1H), 2.98 (dd, *J* = 16.8, 12.8 Hz, 1H), 2.84 (dd, *J* = 16.8, 3.3 Hz, 1H), 2.30 (d, *J* = 2.1 Hz, 3H). ^13^C NMR (126 MHz, CDCl_3_) δ 190.65, 160.57, 147.14, 146.87, 144.98, 144.70, 136.39, 132.08, 127.04, 122.09, 122.04, 121.09, 118.10, 109.74, 109.67, 108.70, 108.68, 108.24, 108.16, 36.47, 21.30. HRMS (ESI) [M+H]^+^ calcd for C_17_H_15_O_4_: 283.0963, found: 283.0965.

**2-(5-(hydroxymethyl)benzo[*d*][1,3]dioxol-2-yl)chroman-4-one (3c).** A yellow liquid, yield 35%; ^1^H NMR (500 MHz, CDCl_3_) δ 7.88 (dd, *J* = 7.8, 1.3 Hz, 1H), 7.52 – 7.48 (m, 1H), 7.08 – 7.01 (m, 2H), 6.91 – 6.87 (m, 1H), 6.86 – 6.78 (m, 2H), 6.39 (d, *J* = 3.4 Hz, 1H), 4.74 (dt, *J* = 12.7, 3.4 Hz, 1H), 4.59 (dt, *J* = 3.8, 0.6 Hz, 2H), 3.00 – 2.93 (m, 1H), 2.82 (ddd, *J* = 16.8, 3.4, 1.2 Hz, 1H). ^13^C NMR (126 MHz, CDCl_3_) δ 190.57, 160.52, 147.54, 147.27, 146.79, 146.51, 136.46, 135.41, 127.06, 122.16, 121.06, 120.99, 118.09, 109.05, 108.44, 108.36, 108.06, 107.98, 65.26, 36.45. HRMS (ESI) [M+Na]^+^ calcd for C_17_H_14_O_5_Na: 321.0731, found: 321.0733.

**2-(5-bromobenzo[*d*][1,3]dioxol-2-yl)chroman-4-one** **(3d).** A yellow solid, yield 14%; m. p. 124.8-126.1 ℃; ^1^H NMR (500 MHz, CDCl_3_) δ 7.89 (dd, *J* = 7.9, 1.7 Hz, 1H), 7.52 – 7.48 (m, 1H), 7.08 – 7.04 (m, 1H), 7.02 (dt, *J* = 8.4, 1.3 Hz, 1H), 7.00 – 6.96 (m, 2H), 6.71 (dd, *J* = 11.9, 8.2 Hz, 1H), 6.39 (dd, *J* = 3.3, 0.6 Hz, 1H), 4.778 – 4.72 (m, 1H), 2.99 – 2.92 (m, 1H), 2.85 – 2.78 (m, 1H). ^13^C NMR (126 MHz, CDCl_3_) δ 190.23, 160.40, 148.24, 147.97, 146.72, 146.46, 136.49, 127.07, 124.88, 124.86, 122.23, 121.03, 118.05, 113.60, 112.49, 112.40, 109.79, 109.73, 36.47, 36.45. HRMS (ESI) [M–H]^-^ calcd for C_16_H_10_O_4_Br: 344.9770, found: 344.9757.

**2-(4-oxochroman-2-yl)benzo[*d*][1,3]dioxole-5-carbonitrile (3e).** A yellow solid, yield 71%; m. p. 114.7-115.9 ℃; ^1^H NMR (400 MHz, CDCl_3_) δ 7.88 (dd, *J* = 7.8, 1.8 Hz, 1H), 7.52 – 7.48 (m, 1H), 7.28 – 7.24 (m, 1H), 7.11 – 7.05 (m, 2H), 6.98 (ddd, *J* = 8.4, 2.5, 1.0 Hz, 1H), 6.98 (dt, J = 8.4, 1.4 Hz, 1H), 6.91 (dd, J = 16.6, 8.1 Hz, 1H), 6.47 (d, J = 3.1 Hz, 1H), 4.79 (dt, J = 12.4, 3.4 Hz, 1H), 3.01 – 2.94 (m, 1H), 2.86 – 2.80 (m, 1H). ^13^C NMR (101 MHz, CDCl_3_) δ 189.67, 160.14, 151.12, 150.86, 147.75, 147.48, 136.44, 128.57, 128.53, 126.99, 122.26, 120.90, 118.63, 117.90, 111.53, 111.46, 110.37, 110.35, 109.25, 109.19, 105.51, 105.48, 76.54, 36.39. HRMS (ESI) [M+H]^+^ calcd for C_17_H_12_O_4_N: 294.0749, found: 294.0761.

**methyl 2-(4-oxochroman-2-yl)benzo[*d*][1,3]dioxole-5-carboxylate (3f).** A white solid, yield 70%; m. p. 103.4-104.6 ℃; ^1^H NMR (500 MHz, CDCl_3_) δ 7.85 (dd, *J* = 7.9, 1.8 Hz, 1H), 7.65 (ddd, *J* = 8.2, 5.7, 1.7 Hz, 1H), 7.49 – 7.43 (m, 2H), 7.05 – 7.01 (m, 1H), 7.00 – 6.96 (m, 1H), 6.84 (dd, *J* = 14.7, 8.2 Hz, 1H), 6.43 (dd, *J* = 3.3, 1.8 Hz, 1H), 4.77 – 4.72 (m, 1H), 3.86 (d, *J* = 2.3 Hz, 3H), 2.98 – 2.91 (m, 1H), 2.82 – 2.76 (m, 1H). ^13^C NMR (126 MHz, CDCl_3_) δ 190.17, 166.35, 166.34, 160.36, 151.18, 150.92, 147.47, 147.21, 136.47, 127.05, 127.03, 125.73, 125.66, 124.65, 122.29, 122.13, 121.00, 118.11, 117.95, 109.90, 109.84, 109.70, 109.59, 108.14, 52.28, 36.45, 36.39. HRMS (ESI) [M+H]^+^ calcd for C_18_H_15_O_6_: 327.0862, found: 327.0863.

**ethyl 2-(4-oxochroman-2-yl)benzo[*d*][1,3]dioxole-5-carboxylate (3g).** A colorless liquid, yield 60%; ^1^H NMR (500 MHz, CDCl_3_) δ 7.89 – 7.85 (m, 1H), 7.67 (ddd, *J* = 8.2, 5.5, 1.7 Hz, 1H), 7.51 – 7.46 (m, 2H), 7.06 – 7.02 (m, 1H), 6.99 (dt, *J* = 8.4, 1.2 Hz, 1H), 6.85 (dd, *J* = 13.9, 8.2 Hz, 1H), 6.44 (dd, *J* = 3.3, 2.3 Hz, 1H), 4.78 – 4.73 (m, 1H), 4.36 – 4.30 (m, 2H), 2.99 – 2.92 (m, 1H), 2.81 (ddd, *J* = 16.7, 3.4, 1.0 Hz, 1H), 1.36 (td, *J* = 7.1, 2.4 Hz, 3H). ^13^C NMR (126 MHz, CDCl_3_) δ 190.20, 165.89, 165.87, 160.39, 151.10, 150.83, 147.44, 147.18, 136.48, 127.05, 125.64, 125.61, 125.04, 122.22, 121.01, 118.05, 109.84, 109.81, 109.70, 109.61, 108.13, 108.06, 61.13, 61.11, 36.49, 36.42, 14.44. HRMS (ESI) [M+H]^+^ calcd for C_19_H_17_O_6_: 341.1019, found: 341.1020.

**2-(5-butyrylbenzo[*d*][1,3]dioxol-2-yl)chroman-4-one (3h).** A yellow solid, yield 60%, m. p. 94.1-94.9 ℃; ^1^H NMR (500 MHz, CDCl_3_) δ 7.81 (dd, *J* = 7.9, 1.8 Hz, 1H), 7.54 – 7.51 (m, 1H), 7.45 – 7.38 (m, 2H), 7.00 – 6.96 (m, 1H), 6.93 (dt, *J* = 8.5, 1.4 Hz, 1H), 6.82 (dd, *J* = 14.4, 8.2 Hz, 1H), 6.40 (dd, *J* = 3.3, 2.5 Hz, 1H), 4.74 – 4.69 (m, 1H), 2.94 – 2.87 (m, 1H), 2.81 (td, *J* = 7.3, 4.6 Hz, 2H), 2.76 (ddd, *J* = 16.7, 3.4, 1.0 Hz, 1H), 1.71 – 1.65 (m, 2H), 0.94 (td, *J* = 7.4, 2.0 Hz, 3H). ^13^C NMR (126 MHz, CDCl_3_) δ 198.45, 198.42, 190.12, 160.35, 151.13, 150.87, 147.91, 147.63, 136.43, 132.38, 132.36, 127.00, 124.54, 124.51, 122.18, 120.98, 118.00, 109.86, 108.05, 108.01, 107.96, 107.94, 40.35, 36.43, 36.36, 18.01, 13.99. HRMS (ESI) [M+H]^+^ calcd for C_20_H_19_O_5_: 339.1223, found: 339.1227.

***N*-benzyl-*N*-methyl-2-(4-oxochroman-2-yl)benzo[*d*][1,3]dioxole-5-carboxamide (3i).** A yellow solid, yield 58%, m. p. 103.6-104.5 ℃; ^1^H NMR (500 MHz, CDCl_3_) δ 7.85 (dd, *J* = 7.8, 1.8 Hz, 1H), 7.49 – 7.45 (m, 1H), 7.35 – 7.30 (m, 2H), 7.20 (dd, *J* = 8.2, 2.4 Hz, 2H), 7.14 – 7.11 (m, 2H), 7.05 – 7.01 (m, 1H), 6.98 (ddd, *J* = 8.5, 3.2, 1.0 Hz, 1H), 6.82 – 6.77 (m, 1H), 6.60 – 6.53 (m, 1H), 6.39 (dd, *J* = 3.3, 1.8 Hz, 1H), 4.73 – 4.68 (m, 1H), 4.53 (d, *J* = 5.7 Hz, 2H), 2.95 – 2.87 (m, 1H), 2.79 – 2.73 (m, 1H), 2.32 (s, 3H)^.^ ^13^C NMR (126 MHz, CDCl_3_) δ 190.30, 166.54, 160.40, 149.94, 149.67, 147.70, 147.43, 137.39, 136.50, 135.25, 129.52, 129.21, 128.02, 128.00, 127.04, 122.21, 122.03, 122.00, 120.99, 118.05, 109.74, 108.15, 108.09, 107.96, 107.89, 44.03, 36.39, 21.23. HRMS (ESI) [M+H]^+^ calcd for C_25_H_22_O_5_N: 416.1490, found: 416.1490.

**2-(5-((1*S*,3*R*,4*S*,6*R*)-4-(benzo[*d*][1,3]dioxol-5-yl)tetrahydro-1*H*,3*H*-furo[3,4-*c*]furan-1-yl)benzo[*d*][1,3]dioxol-2-yl)chroman-4-one (3j).** A white solid, yield 35%, m. p. 117.6-118.7 ℃; ^1^H NMR (500 MHz, CDCl_3_) δ 7.89 – 7.86 (m, 1H), 7.52 – 7.47 (m, 1H), 7.07 – 7.01 (m, 2H), 6.88 – 6.85 (m, 1H), 6.85 – 6.81 (m, 2H), 6.81 – 6.76 (m, 3H), 6.38 (dt, *J* = 3.5, 2.2 Hz, 1H), 5.95 (s, 2H), 4.77 – 4.72 (m, 1H), 4.72 – 4.69 (m, 2H), 4.26 – 4.20 (m, 2H), 3.89 – 3.85 (m, 2H), 3.08 – 3.03 (m, 2H), 3.00 – 2.92 (m, 1H), 2.81 (ddd, *J* = 16.8, 3.4, 2.0 Hz, 1H). ^13^C NMR (126 MHz, CDCl_3_) δ 190.53, 160.51, 148.06, 147.68, 147.42, 147.38, 147.21, 146.79, 146.52, 136.45, 135.63, 135.61, 135.02, 127.06, 122.16, 121.06, 119.90, 119.50, 118.08, 109.11, 108.43, 108.31, 101.20, 85.85, 85.77, 71.86, 71.74, 54.44, 54.35, 36.46, 29.81. HRMS (ESI) [M+Na]^+^ calcd for C_29_H_24_O_8_Na: 523.1352, found: 523.1363.

**2-(phenoxymethyl)chroman-4-one (3k).** A yellow liquid, yield 10%; ^1^H NMR (500 MHz, CDCl_3_) δ 7.90 (ddd, *J* = 7.8, 1.8, 0.7 Hz, 1H), 7.49 (ddd, *J* = 8.1, 7.3, 1.8 Hz, 1H), 7.33 – 7.28 (m, 2H), 7.06 – 7.01 (m, 2H), 6.99 (tt, *J* = 7.4, 1.1 Hz, 1H), 6.96 – 6.93 (m, 2H), 4.83 (dtd, *J* = 12.9, 4.5, 3.0 Hz, 1H), 4.28 (d, *J* = 0.9 Hz, 1H), 4.27 (d, *J* = 1.4 Hz, 1H), 3.04 (dd, *J* = 16.9, 13.0 Hz, 1H), 2.81 (dd, *J* = 16.9, 3.0 Hz, 1H). ^13^C NMR (126 MHz, CDCl_3_) δ 191.80, 161.24, 158.35, 136.31, 129.71, 127.09, 121.76, 121.58, 120.99, 118.16, 114.72, 69.16, 39.70. HRMS (ESI) [M+H]^+^ calcd for C_16_H_15_O_3_: 255.1013, found: 255.1016.

**2-(1-propoxypropyl)chroman-4-one (3l).** A yellow liquid, yield 26%; ^1^H NMR (600 MHz, CDCl_3_) δ 7.87 (dt, *J* = 8.0, 2.2 Hz, 2H), 7.49 – 7.44 (m, 2H), 7.02 – 6.96 (m, 4H), 4.52 (dt, *J* = 13.5, 3.3 Hz, 1H), 4.42 (dt, *J* = 12.8, 3.7 Hz, 1H), 3.64 – 3.47 (m, 5H), 3.35 (dt, *J* = 8.6, 4.0 Hz, 1H), 2.94 (ddd, *J* = 27.9, 16.9, 13.1 Hz, 2H), 2.73 (dd, *J* = 16.9, 3.1 Hz, 1H), 2.63 (dd, *J* = 16.6, 2.8 Hz, 1H), 1.82 – 1.75 (m, 1H), 1.69 (dt, *J* = 14.1, 7.3 Hz, 1H), 1.64 – 1.56 (m, 6H), 1.01 (dt, *J* = 11.1, 7.4 Hz, 6H), 0.93 (q, *J* = 7.2 Hz, 6H). ^13^C NMR (151 MHz, CDCl_3_) δ 193.06, 193.03, 161.70, 161.50, 136.04, 136.00, 127.00, 126.94, 121.37, 121.33, 121.15, 118.19, 117.99, 81.82, 81.70, 79.72, 73.40, 73.36, 39.31, 38.41, 23.91, 23.47, 23.40, 23.04, 10.80, 10.78, 10.25, 9.94. HRMS (ESI) [M+H]^+^ calcd for C_15_H_21_O_3_: 249.1482, found: 249.1485.

**2-(1-butoxybutyl)chroman-4-one (3m).** A yellow liquid, yield 46%; ^1^H NMR (600 MHz, CDCl_3_) δ 7.87 (dt, *J* = 8.0, 1.9 Hz, 2H), 7.46 (ddt, *J* = 8.9, 7.5, 1.9 Hz, 2H), 6.99 (q, *J* = 9.6, 8.4 Hz, 4H), 4.50 (dt, *J* = 13.6, 3.2 Hz, 1H), 4.41 (dt, *J* = 13.0, 3.3 Hz, 1H), 3.67 (dt, *J* = 9.1, 6.5 Hz, 1H), 3.62 (dt, *J* = 8.1, 3.9 Hz, 1H), 3.60 – 3.52 (m, 3H), 3.46 – 3.42 (m, 1H), 2.98 – 2.94 (m, 1H), 2.93 – 2.89 (m, 1H), 2.72 – 2.67 (m, 1H), 2.63 (dd, *J* = 16.7, 2.7 Hz, 1H), 1.60 – 1.51 (m, 8H), 1.37 (p, *J* = 7.5 Hz, 8H), 0.99 – 0.94 (m, 6H), 0.90 (d, *J* = 7.5 Hz, 6H). ^13^C NMR (151 MHz, CDCl_3_) δ 193.06, 161.70, 161.53, 136.03, 136.00, 127.00, 126.95, 121.37, 121.31, 121.16, 121.13, 118.18, 117.98, 80.32, 80.24, 71.53, 71.44, 39.23, 38.25, 33.36, 32.38, 32.30, 19.45, 19.08, 18.90, 14.34, 14.25, 14.08, 14.01. HRMS (ESI) [M+H]^+^ calcd for C_17_H_25_O_3_: 277.1796, found: 277.1798.

**2-(*tert*-butoxymethyl)chroman-4-one (3n).** A yellow liquid, yield 40%; ^1^H NMR (500 MHz, CDCl_3_) δ 7.86 (dd, *J* = 8.0, 1.8 Hz, 1H), 7.45 (ddd, *J* = 8.4, 7.2, 1.8 Hz, 1H), 6.99 (dtd, *J* = 8.1, 3.4, 1.1 Hz, 2H), 4.56 (dtd, *J* = 12.5, 4.7, 3.2 Hz, 1H), 3.66 (d, *J* = 4.7 Hz, 2H), 2.87 (dd, *J* = 16.9, 12.4 Hz, 1H), 2.72 (dd, *J* = 16.9, 3.2 Hz, 1H), 1.21 (s, 9H). ^13^C NMR (126 MHz, CDCl_3_) δ 192.60, 161.54, 136.02, 126.91, 121.32, 121.10, 118.16, 73.72, 63.62, 40.02, 27.48. HRMS (ESI) [M+Na]^+^ calcd for C_14_H_18_O_3_Na: 257.1146, found: 257.1148.

**2-(dimethoxymethyl)chroman-4-one (3oA).** A yellow liquid, yield 38%; ^1^H NMR (500 MHz, CDCl_3_) δ 7.88 (dd, *J* = 8.0, 1.8 Hz, 1H), 7.48 (ddd, *J* = 8.4, 7.2, 1.8 Hz, 1H), 7.05 – 7.00 (m, 2H), 4.56 (d, *J* = 4.9 Hz, 1H), 4.52 (ddd, *J* = 11.9, 4.9, 3.6 Hz, 1H), 3.51 (d, *J* = 0.9 Hz, 6H), 2.87 (dd, *J* = 17.0, 11.9 Hz, 1H), 2.79 (dd, *J* = 17.0, 3.6 Hz, 1H). ^13^C NMR (126 MHz, CDCl_3_) δ 191.98, 136.16, 126.98, 121.68, 121.21, 118.06, 104.39, 56.27, 55.10, 37.86. HRMS (ESI) [M+Na]^+^ calcd for C_12_H_14_O_4_Na: 245.0784, found: 245.0784.

**2-((methoxymethoxy)methyl)chroman-4-one (3oB).** A yellow liquid, yield 19%; ^1^H NMR (500 MHz, CDCl_3_) δ 7.89 (dt, *J* = 7.9, 1.5 Hz, 1H), 7.48 (ddt, *J* = 8.2, 6.9, 1.5 Hz, 1H), 7.05 – 6.93 (m, 2H), 4.75 – 4.70 (m, 2H), 4.69 – 4.63 (m, 1H), 3.87 (ddd, *J* = 11.1, 3.6, 1.1 Hz, 1H), 3.83 (ddd, *J* = 11.1, 5.2, 1.2 Hz, 1H), 3.40 (d, *J* = 1.2 Hz, 3H), 2.93 (ddd, *J* = 16.9, 13.0, 1.1 Hz, 1H), 2.69 (ddd, *J* = 16.8, 3.1, 1.1 Hz, 1H). ^13^C NMR (126 MHz, CDCl_3_) δ 192.07, 161.36, 136.23, 127.04, 121.63, 120.94, 118.12, 96.74, 68.75, 55.58, 39.64. HRMS (ESI) [M+Na]^+^ calcd for C_12_H_14_O_4_Na: 245.0784, found: 245.0784.

**2-(1,2-dimethoxyethyl)chroman-4-one (3pA).** A yellow liquid, yield 38%; ^1^H NMR (500 MHz, CDCl_3_) δ 7.88 (dd, *J* = 7.9, 1.7 Hz, 1H), 7.47 (ddd, *J* = 8.3, 7.2, 1.8 Hz, 1H), 7.03 – 6.96 (m, 2H), 4.61 (ddd, *J* = 12.9, 4.4, 3.1 Hz, 1H), 3.71 – 3.67 (m, 1H), 3.59 – 3.57 (m, 2H), 3.54 (s, 3H), 3.39 (s, 3H), 2.96 – 2.90 (m, 1H), 2.77 (dd, *J* = 17.0, 3.1 Hz, 1H). ^13^C NMR (126 MHz, CDCl_3_) δ 192.62, 136.07, 127.05, 121.53, 121.15, 118.03, 80.82, 70.79, 59.55, 59.25, 38.39. HRMS (ESI) [M+Na]^+^ calcd for C_13_H_16_O_4_Na: 259.0938, found: 259.0941.

**2-((2-methoxyethoxy)methyl)chroman-4-one (3pB).** A yellow liquid, yield 19%; ^1^H NMR (500 MHz, CDCl_3_) δ 7.86 (dd, *J* = 8.1, 1.7 Hz, 1H), 7.46 (ddd, *J* = 8.4, 7.2, 1.8 Hz, 1H), 7.03 – 6.97 (m, 2H), 4.64 (dtd, *J* = 13.1, 4.5, 3.0 Hz, 1H), 3.81 (d, *J* = 4.5 Hz, 2H), 3.75 – 3.71 (m, 2H), 3.57 (t, *J* = 4.6 Hz, 2H), 3.38 (s, 3H), 2.89 (dd, *J* = 16.9, 13.2 Hz, 1H), 2.68 (dd, *J* = 16.9, 3.0 Hz, 1H). ^13^C NMR (126 MHz, CDCl_3_) δ 192.23, 161.38, 136.16, 127.01, 121.54, 120.98, 118.17, 72.80, 72.04, 71.29, 59.26, 39.67. HRMS (ESI) [M+Na]^+^ calcd for C_13_H_16_O_4_Na: 259.0939, found: 259.0941.

**1-(4-oxochroman-2-yl)ethyl acetate (3q).** A yellow liquid, yield 26%; ^1^H NMR (500 MHz, CDCl_3_) δ 7.88 (dt, *J* = 1.8, 0.6 Hz, 1H), 7.87 (dt, *J* = 1.8, 0.6 Hz, 1H), 7.49 (ddt, *J* = 8.3, 7.2, 1.8 Hz, 2H), 7.05 – 7.00 (m, 4H), 5.26 – 5.22 (m, 1H), 5.22 – 5.17 (m, 1H), 4.51 – 4.47 (m, 1H), 4.47 – 4.43 (m, 1H), 2.83 (ddd, *J* = 16.7, 13.1, 11.8 Hz, 2H), 2.73 (dd, *J* = 16.8, 3.1 Hz, 1H), 2.65 (dd, *J* = 16.7, 3.0 Hz, 1H), 2.10 (s, 3H), 2.08 (s, 3H), 1.41 (d, *J* = 6.6 Hz, 3H), 1.37 (d, *J* = 6.5 Hz, 3H). ^13^C NMR (126 MHz, CDCl_3_) δ 178.60, 169.99, 156.61, 133.55, 125.74, 125.00, 123.78, 117.96, 109.89, 34.14, 28.94, 22.22, 13.86. HRMS (ESI) [M+Na]^+^ calcd for C_13_H_14_O_4_Na: 257.0784, found: 257.0784.

**2-(tetrahydrofuran-2-yl)chroman-4-one (3r).** A yellow liquid, yield 40%; ^1^H NMR (**Isomer 1**, 500 MHz, CDCl_3_) δ 7.87 (dd, *J* = 7.8, 1.8 Hz, 1H), 7.47 (ddd, *J* = 8.2, 7.2, 1.8 Hz, 1H), 7.07 – 6.96 (m, 2H), 4.39 (ddd, *J* = 12.2, 5.0, 3.7 Hz, 1H), 4.20 – 4.15 (m, 1H), 3.97 – 3.89 (m, 1H), 3.84 (dt, *J* = 8.3, 6.6 Hz, 1H), 2.82 (dd, *J* = 16.8, 12.3 Hz, 1H), 2.76 (dd, *J* = 16.8, 3.7 Hz, 1H), 2.18 – 2.05 (m, 1H), 2.01 – 1.83 (m, 3H). ^13^C NMR (**Isomer 1**, 126 MHz, CDCl_3_) δ 192.37, 161.47, 136.10, 126.99, 121.47, 121.13, 118.08, 69.12, 39.07, 27.62, 25.77. HRMS (**Isomer 1**, ESI) [M+Na]^+^ calcd for C_13_H_14_O_3_Na: 241.0834, found: 241.0835.

^1^H NMR (**Isomer 2**, 500 MHz, CDCl_3_) δ 7.86 (dd, *J* = 7.9, 1.7 Hz, 1H), 7.47 (ddd, *J* = 8.4, 7.2, 1.8 Hz, 1H), 7.07 – 6.98 (m, 2H), 4.40 (ddd, *J* = 13.2, 5.3, 2.9 Hz, 1H), 4.10 (ddd, *J* = 7.8, 6.8, 5.3 Hz, 1H), 3.95 – 3.84 (m, 2H), 2.89 (dd, *J* = 16.8, 13.2 Hz, 1H), 2.64 (dd, *J* = 16.7, 2.9 Hz, 1H), 2.09 – 1.99 (m, 2H), 1.99 – 1.90 (m, 1H), 1.89 – 1.81 (m, 1H). ^13^C NMR (**Isomer 2**, 126 MHz, CDCl_3_) δ 192.28, 161.43, 136.20, 126.93, 121.48, 120.99, 118.20, 79.88, 69.02, 39.90, 27.61, 26.00. HRMS (**Isomer 2**, ESI) [M+H]^+^ calcd for C_13_H_15_O_3_: 219.1009, found: 219.1016.

**2-chloro-10-methylindolo[2,1-*b*]quinazoline-6,12-dione (3s).** A yellow liquid, yield 48%; ^1^H NMR (**Isomer 1**, 400 MHz, CDCl_3_) δ 7.87 (dd, *J* = 8.0, 1.7 Hz, 1H), 7.46 (td, *J* = 7.7, 7.1, 1.8 Hz, 1H), 7.00 (dt, *J* = 7.4, 3.1 Hz, 2H), 4.33 (ddd, *J* = 12.7, 4.5, 3.1 Hz, 1H), 4.10 – 4.04 (m, 1H), 3.64 (ddd, *J* = 11.1, 4.6, 2.1 Hz, 1H), 3.51 (td, *J* = 11.4, 2.7 Hz, 1H), 2.91 (dd, *J* = 17.0, 12.8 Hz, 1H), 2.75 (dd, *J* = 17.0, 3.1 Hz, 1H), 1.93 (dq, *J* = 9.2, 2.8 Hz, 1H), 1.82 – 1.74 (m, 1H), 1.67 – 1.52 (m, 3H), 1.51 – 1.35 (m, 1H). ^13^C NMR (**Isomer 1**, 101 MHz, CDCl_3_) δ 192.62, 161.40, 135.96, 126.89, 121.36, 121.08, 118.07, 80.10, 68.80, 38.56, 27.30, 25.91, 23.02. HRMS (**Isomer 1**, ESI) [M+H]^+^ calcd for C_14_H_17_O_3_: 233.1171, found: 233.1172.

^1^H NMR (**Isomer 2**, 400 MHz, CDCl_3_) δ 7.87 (dd, *J* = 7.8, 1.7 Hz, 1H), 7.47 (ddd, *J* = 8.7, 7.2, 1.8 Hz, 1H), 7.09 – 6.95 (m, 2H), 4.38 (ddd, *J* = 13.5, 4.0, 2.8 Hz, 1H), 4.12 (ddd, *J* = 11.5, 4.4, 2.1 Hz, 1H), 3.60 – 3.45 (m, 2H), 3.01 (dd, *J* = 16.9, 13.5 Hz, 1H), 2.61 (dd, *J* = 17.0, 2.8 Hz, 1H), 2.02 – 1.92 (m, 1H), 1.75 (td, *J* = 12.9, 12.4, 3.9 Hz, 1H), 1.67 – 1.53 (m, 4H). ^13^C NMR (**Isomer 2**, 101 MHz, CDCl_3_) δ 192.81, 161.49, 135.95, 126.82, 121.37, 120.97, 118.25, 69.13, 39.38, 27.01, 25.83, 23.19. HRMS (**Isomer 2,** ESI) [M+H]^+^ calcd for C_14_H_17_O_3_: 233.1163, found: 233.1172.

**(*S*)-2-((*S*)-1,4-dioxan-2-yl)chroman-4-one (3t).** A yellow liquid, yield 23%; ^1^H NMR (**Isomer 1**, 500 MHz, CDCl_3_) δ 7.87 (dd, *J* = 7.9, 1.8 Hz, 1H), 7.47 (ddd, *J* = 8.8, 7.2, 1.8 Hz, 1H), 7.04 – 6.95 (m, 2H), 4.39 (ddd, *J* = 10.2, 6.5, 5.3 Hz, 1H), 4.04 (dd, *J* = 11.5, 2.7 Hz, 1H), 3.89 – 3.71 (m, 4H), 3.67 – 3.60 (m, 1H), 3.52 (dd, *J* = 11.5, 9.9 Hz, 1H), 2.85 – 2.81 (m, 2H). ^13^C NMR (**Isomer 1**, 126 MHz, CDCl_3_) δ 191.69, 160.85, 136.23, 127.07, 121.82, 121.16, 118.01, 75.61, 68.24, 66.82, 66.61, 39.14. HRMS (**Isomer 1**, ESI) [M+H]^+^ calcd for C_13_H_15_O_4_: 235.0963, found: 235.0965.

**(*R*)-2-((*R*)-1,4-dioxan-2-yl)chroman-4-one (3t’).** A yellow liquid, yield 25%; ^1^H NMR (**Isomer 2**, 500 MHz, CDCl_3_) δ 7.87 (dd, *J* = 8.2, 1.8 Hz, 1H), 7.47 (ddd, *J* = 8.1, 7.2, 1.8 Hz, 1H), 7.05 – 6.98 (m, 2H), 4.45 (dt, *J* = 13.6, 3.0 Hz, 1H), 3.94 – 3.90 (m, 1H), 3.87 – 3.66 (m, 6H), 3.06 (dd, *J* = 16.9, 13.5 Hz, 1H), 2.57 (dd, *J* = 16.9, 2.8 Hz, 1H). ^13^C NMR (**Isomer 2**, 126 MHz, CDCl_3_) δ 192.11, 161.11, 136.18, 127.00, 121.82, 120.97, 118.17, 75.81, 67.53, 67.48, 66.53, 39.03. HRMS (**Isomer 2**, ESI) [M+H]^+^ calcd for C_13_H_15_O_4_: 235.0956, found: 235.0965.

(Known compound: *Chem. Commun.*, 2024, **60**, 1265-1268.)

**2-(1,3-dioxolan-2-yl)chroman-4-one (3u).** A yellow solid, yield 56%; m. p. 60.8-61.4 ℃; ^1^H NMR (500 MHz, CDCl_3_) δ 7.88 – 7.84 (m, 1H), 7.49 – 7.45 (m, 1H), 7.04 – 6.99 (m, 2H), 5.19 (d, *J* = 3.5 Hz, 1H), 4.52 (dt, *J* = 12.2, 3.6 Hz, 1H), 4.08 – 3.93 (m, 4H), 2.88 (dd, *J* = 16.9, 12.1 Hz, 1H), 2.76 (dd, *J* = 16.9, 3.6 Hz, 1H). ^13^C NMR (126 MHz, CDCl_3_) δ 191.52, 160.81, 136.20, 126.89, 121.70, 121.16, 118.04, 103.06, 77.68, 65.89, 65.58, 37.19. HRMS (ESI) [M+H]^+^ calcd for C_12_H_13_O_4_: 221.0806, found: 221.0808.

**2-(hydroxymethyl)chroman-4-one (3v).** A yellow solid, yield 51%; m. p. 74.2-75.6 ℃; ^1^H NMR (500 MHz, CDCl_3_) δ 7.86 (dd, *J* = 7.9, 1.8 Hz, 1H), 7.46 (ddd, *J* = 8.4, 7.2, 1.8 Hz, 1H), 7.04 – 6.95 (m, 2H), 4.58 – 4.52 (m, 1H), 3.97 (dd, *J* = 12.3, 3.1 Hz, 1H), 3.84 (dd, *J* = 12.2, 5.3 Hz, 1H), 2.92 (dd, *J* = 16.9, 13.5 Hz, 1H), 2.62 (dd, *J* = 16.9, 2.9 Hz, 1H). ^13^C NMR (126 MHz, CDCl_3_) δ 192.41, 161.25, 136.35, 127.11, 121.74, 120.88, 117.96, 78.27, 64.50, 39.09. HRMS (ESI) [M+H]^+^ calcd for C_10_H_10_O_3_: 179.0700, found: 179.0703.

**2-(1-hydroxyethyl)chroman-4-one (3w).** A yellow liquid, yield 57%; ^1^H NMR (600 MHz, CDCl_3_) δ 7.87 (ddd, *J* = 7.9, 3.2, 1.8 Hz, 2H), 7.48 (tdd, *J* = 8.3, 7.1, 1.8 Hz, 2H), 7.06 – 6.96 (m, 4H), 4.37 (dt, *J* = 13.8, 3.0 Hz, 1H), 4.27 (ddd, *J* = 13.6, 5.5, 2.8 Hz, 1H), 4.23 (dd, *J* = 6.6, 3.4 Hz, 1H), 3.98 (p, *J* = 6.3 Hz, 1H), 2.90 (ddd, *J* = 48.2, 16.8, 13.7 Hz, 2H), 2.66 (ddd, *J* = 16.8, 8.6, 2.8 Hz, 2H), 1.33 (d, *J* = 6.4 Hz, 3H), 1.27 (d, *J* = 6.6 Hz, 3H). ^13^C NMR (151 MHz, CDCl_3_) δ 192.70, 192.17, 161.33, 161.01, 136.30, 136.24, 127.12, 127.09, 121.82, 121.69, 121.01, 120.99, 117.91, 81.65, 81.33, 69.38, 68.51, 39.71, 36.98, 18.57, 17.72. HRMS (ESI) [M+H]^+^ calcd for C_11_H_13_O_3_: 193.0859, found: 193.0859.

**2-(2-hydroxypropan-2-yl)chroman-4-one (3x).** A yellow liquid, yield 45%; ^1^H NMR (500 MHz, CDCl_3_) δ 7.89 (dd, *J* = 7.7, 2.0 Hz, 1H), 7.52 – 7.47 (m, 1H), 7.03 (dd, *J* = 15.3, 7.9 Hz, 2H), 4.28 – 4.23 (m, 1H), 2.87 (dd, *J* = 16.6, 14.3 Hz, 1H), 2.72 (dd, *J* = 16.6, 2.4 Hz, 1H), 1.40 (s, 3H), 1.31 (s, 3H). ^13^C NMR (126 MHz, CDCl_3_) δ 192.69, 161.28, 136.20, 127.08, 121.74, 120.89, 117.89, 83.90, 71.64, 38.29, 26.01, 24.61. HRMS (ESI) [M+Na]^+^ calcd for C_12_H_14_O_3_Na: 229.0827, found: 229.0835.

***tert*-butyl 2-(4-oxochroman-2-yl)pyrrolidine-1-carboxylate (4a).** A yellow liquid, yield 46%; ^1^H NMR (600 MHz, CDCl_3_) δ 7.87 – 7.76 (m, 1H), 7.51 – 7.38 (m, 1H), 7.02 – 6.90 (m, 2H), 4.83 – 4.48 (m, 1H), 4.31 – 3.90 (m, 1H), 3.64 – 3.29 (m, 2H), 2.79 – 2.53 (m, 2H), 2.11 – 1.83 (m, 4H), 1.43 (s, 9H). ^13^C NMR (151 MHz, CDCl_3_) δ 192.92, 192.33, 161.61, 155.35, 154.63, 135.97, 127.11, 127.02, 121.38, 121.05, 118.09, 117.98, 80.28, 78.91, 78.50, 78.02, 59.93, 58.85, 47.46, 47.24, 40.65, 39.18, 28.57, 28.53, 27.86, 26.71, 25.82, 24.59, 24.27, 23.70, 23.41. HRMS (ESI) [M+Na]^+^ calcd for C_18_H_23_O_4_NNa: 340.1505, found: 340.1519.

**2-(diphenylphosphoryl)chroman-4-one (4b).** A yellow solid, yield 60%, m. p. 90.2-91.3 ℃; ^1^H NMR (500 MHz, CDCl_3_) δ 7.97 – 7.92 (m, 2H), 7.91 – 7.85 (m, 2H), 7.83 (dd, *J* = 7.8, 1.7 Hz, 1H), 7.61 – 7.55 (m, 2H), 7.54 – 7.48 (m, 4H), 7.44 (ddd, *J* = 8.7, 7.2, 1.8 Hz, 1H), 7.01 (ddd, *J* = 8.0, 7.2, 1.1 Hz, 1H), 6.93 (dd, *J* = 8.5, 1.0 Hz, 1H), 5.30 (dt, *J* = 13.8, 3.7 Hz, 1H), 3.00 – 2.89 (m, 2H). ^13^C NMR (126 MHz, CDCl_3_) ^13^C δ 189.87, 189.76, 160.90, 160.81, 136.27, 132.88, 132.86, 132.84, 132.42, 132.35, 131.61, 131.54, 130.80, 130.72, 130.20, 129.40, 129.08, 128.99, 128.90, 128.81, 128.59, 127.79, 127.32, 122.44, 121.24, 117.91, 75.73, 75.03, 36.13. ^31^P NMR (202 MHz, CDCl_3_) δ 27.75. HRMS (ESI) [M+Na]^+^ calcd for C_21_H_17_O_3_NaP: 371.0800, found: 371.0808.

**2-(di-*p*-tolylphosphoryl)chroman-4-one (4c).** A white solid, yield 32%, m. p. 139.1-140.8 ℃; ^1^H NMR (400 MHz, CDCl_3_) δ 7.83 (d, J = 1.7 Hz, 1H), 7.81 (d, J = 1.6 Hz, 1H), 7.81 – 7.78 (m, 1H), 7.78 – 7.71 (m, 2H), 7.47 – 7.43 (m, 1H), 7.31 (m, 4H), 7.00 (m, 1H), 6.93 (dd, J = 8.4, 1.0 Hz, 1H), 5.25 (dt, J = 13.5, 4.1 Hz, 1H), 3.02 – 2.84 (m, 2H), 2.39 (d, J = 4.4 Hz, 6H). ^13^C NMR (101 MHz, CDCl_3_) δ 189.97, 189.84, 160.95, 160.84, 143.32, 143.29, 143.26, 136.08, 132.36, 132.27, 131.56, 131.46, 129.69, 129.57, 129.54, 129.41, 127.19, 127.16, 126.13, 125.53, 124.50, 122.20, 121.18, 117.83, 74.99, 36.12, 21.68. ^31^P NMR (202 MHz, CDCl_3_) δ 28.37. HRMS (ESI) [M+H]^+^ calcd for C_23_H_22_O_3_P: 377.1301, found: 377.1301.

**2-(bis(4-bromophenyl)phosphoryl)chroman-4-one (4d).** A yellow solid, yield 43%; m. p. 121.1-122.8 ℃; ^1^H NMR (500 MHz, CDCl_3_) δ 7.84 (dd, *J* = 7.9, 1.7 Hz, 1H), 7.81 – 7.72 (m, 4H), 7.70 – 7.65 (m, 4H), 7.47 (ddd, *J* = 8.4, 7.2, 1.8 Hz, 1H), 7.05 (ddd, *J* = 8.0, 7.2, 1.0 Hz, 1H), 6.94 (dd, *J* = 8.3, 1.0 Hz, 1H), 5.29 (dt, *J* = 13.2, 4.1 Hz, 1H), 2.99 – 2.84 (m, 2H). ^13^C NMR (126 MHz, CDCl_3_) δ 189.22, 189.11, 160.53, 160.43, 136.42, 133.87, 133.79, 133.02, 132.94, 132.54, 132.44, 132.40, 132.30, 132.17, 128.93, 128.61, 128.59, 128.50, 128.47, 128.11, 127.48, 127.07, 126.26, 122.77, 121.25, 117.75, 74.77, 36.01. ^31^P NMR (202 MHz, CDCl_3_) δ 28.38. HRMS (ESI) [M+H]^+^ calcd for C_21_H_16_O_3_Br_2_P: 504.9184, found: 504.9198.

**2-(di(naphthalen-2-yl)phosphoryl)chroman-4-one (4e).** A yellow solid, yield 44%; m. p. 94.8-95.7 ℃; ^1^H NMR (500 MHz, CDCl_3_) δ 8.67 (dd, *J* = 13.4, 1.2 Hz, 1H), 8.58 – 8.53 (m, 1H), 7.98 – 7.90 (m, 5H), 7.90 – 7.82 (m, 4H), 7.62 – 7.54 (m, 4H), 7.43 (ddd, *J* = 8.7, 7.2, 1.8 Hz, 1H), 7.02 – 6.95 (m, 2H), 5.60 – 5.47 (m, 1H), 3.14 – 2.98 (m, 2H). ^13^C NMR (126 MHz, CDCl_3_) δ 189.90, 189.79, 160.97, 160.88, 136.33, 135.18, 135.15, 135.13, 135.10, 135.03, 134.11, 134.05, 132.68, 132.58, 129.18, 129.14, 129.06, 128.97, 128.86, 128.74, 128.65, 128.05, 128.02, 127.41, 127.36, 127.33, 126.72, 126.63, 126.50, 125.94, 125.85, 125.82, 125.01, 122.49, 121.31, 117.94, 75.16, 36.30. ^31^P NMR (202 MHz, CDCl_3_) δ 28.38. HRMS (ESI) [M+Na]^+^ calcd for C_29_H_21_O_3_NaP: 471.1118, found: 471.1121.

**2-((methylthio)methyl)chroman-4-one (4f).** A yellow liquid, yield 40%; ^1^H NMR (500 MHz, CDCl_3_ ) δ 7.90 – 7.85 (m, 1H), 7.50 – 7.46 (m, 1H), 7.06 – 6.97 (m, 2H), 4.65 (ddt, *J* = 12.4, 5.8, 3.0 Hz, 1H), 2.98 – 2.92 (m, 1H), 2.91 – 2.86 (m, 2H), 2.81 (dt, *J* = 16.8, 3.2 Hz, 1H), 2.24 (s, 3H). ^13^C NMR (126 MHz, CDCl_3_) δ 191.35, 160.56, 135.53, 126.37, 120.96, 120.27, 117.35, 76.87, 41.31, 37.83, 16.32. HRMS (ESI) [M+H]^+^ calcd for C_11_H_13_O_2_S: 209.0631, found: 209.0631.

**2-(1-(ethylthio)ethyl)chroman-4-one (4g).** A yellow liquid, yield 42%; ^1^H NMR (500 MHz, CDCl_3_) δ 7.86 (dt, *J* = 7.9, 1.6 Hz, 2H), 7.46 (ddd, *J* = 8.6, 7.2, 1.8 Hz, 2H), 7.04 – 6.95 (m, 4H), 4.52 (ddd, *J* = 13.8, 4.1, 2.6 Hz, 1H), 4.46 (ddd, *J* = 13.0, 5.4, 2.8 Hz, 1H), 3.18 – 3.13 (m, 1H), 3.11 (dd, *J* = 7.1, 4.1 Hz, 1H), 3.04 – 2.90 (m, 2H), 2.78 (ddd, *J* = 18.0, 16.7, 2.7 Hz, 2H), 2.72 – 2.60 (m, 4H), 1.43 (d, *J* = 7.1 Hz, 3H), 1.39 (d, *J* = 7.1 Hz, 3H), 1.26 (td, *J* = 7.4, 1.6 Hz, 6H). ^13^C NMR (126 MHz, CDCl_3_) δ 192.83, 192.69, 161.60, 161.32, 136.18, 136.12, 127.06, 127.03, 121.56, 121.03, 121.01, 118.01, 81.58, 43.33, 42.78, 39.88, 39.37, 25.81, 25.79, 17.59, 17.02, 15.01, 14.99. HRMS (ESI) [M+H]^+^ calcd for C_13_H_17_O_2_S: 237.0943, found: 237.0944.

**2-(tetrahydrothiophen-2-yl)chroman-4-one (4h).** A yellow liquid, yield 36%; ^1^H NMR (**Isomer 1,** 600 MHz, CDCl_3_) δ 7.87 (dd, *J* = 7.8, 1.8 Hz, 1H), 7.47 (ddd, *J* = 8.3, 7.2, 1.8 Hz, 1H), 7.03 – 6.96 (m, 2H), 4.29 (ddd, *J* = 12.4, 8.6, 3.0 Hz, 1H), 3.68 (ddd, *J* = 8.6, 6.8, 5.5 Hz, 1H), 2.91 – 2.88 (m, 1H), 2.88 – 2.86 (m, 2H), 2.76 (dd, *J* = 16.7, 12.4 Hz, 1H), 2.23 – 2.16 (m, 1H), 2.11 – 1.99 (m, 3H). ^13^C NMR (**Isomer 1**, 151 MHz, CDCl_3_) δ 192.22, 161.20, 136.17, 127.05, 121.54, 121.15, 117.93, 80.84, 51.41, 41.49, 33.28, 32.75, 30.21. HRMS (**Isomer 1**, ESI) [M+H]^+^ calcd for C_13_H_15_O_2_S: 235.0785, found: 235.0787.

^1^H NMR (**Isomer 2**, 600 MHz, CDCl_3_) δ 7.85 – 7.83 m, 1H), 7.46 (ddd, *J* = 8.3, 7.3, 1.8 Hz, 1H), 7.01 – 6.98 (m, 2H), 4.38 (q, *J* = 7.6 Hz, 1H), 3.70 (dt, *J* = 8.1, 7.0 Hz, 1H), 2.90 (ddd, *J* = 8.4, 5.2, 1.7 Hz, 2H), 2.78 (dd, *J* = 7.9, 0.9 Hz, 2H), 2.24 – 2.13 (m, 2H), 1.99 – 1.91 (m, 1H), 1.76 – 1.69 (m, 1H). ^13^C NMR (**Isomer 2**, 151 MHz, CDCl_3_) δ 192.00, 161.47, 136.26, 126.96, 121.59, 120.96, 118.22, 81.65, 52.05, 41.96, 32.81, 32.48, 31.14. HRMS (**Isomer 2**, ESI) [M+H]^+^ calcd for C_13_H_15_O_2_S: 235.0779, found: 235.0787.

**2-cyclopentylchroman-4-one (4i).** A yellow liquid, yield 32%; ^1^H NMR (500 MHz, CDCl_3_) δ 7.86 (ddd, *J* = 7.8, 1.8, 0.5 Hz, 1H), 7.46 (ddd, *J* = 8.3, 7.2, 1.8 Hz, 1H), 7.02 – 6.95 (m, 2H), 4.28 (ddd, J = 12.5, 8.6, 3.0 Hz, 1H) 2.74 – 2.66 (m, 2H, O=C-CH_2_-)2.74 – 2.66 (m, 2H), 2.28 – 2.18 (m, 1H), 1.97 – 1.90 (m, 1H), 1.82 – 1.74 (m, 1H), 1.70 – 1.65 (m, 2H), 1.63 – 1.52 (m, 3H), 1.36 – 1.27 (m, 1H). ^13^C NMR (126 MHz, CDCl_3_) δ 193.11, 161.94, 136.08, 126.99, 121.16, 121.12, 118.07, 81.83, 44.15, 42.27, 28.92, 28.49, 25.62, 25.50. HRMS (ESI) [M+H]^+^ calcd for C_14_H_17_O_2_: 217.1217, found: 217.1223.

**2-cyclohexylchroman-4-one (4j).** A yellow liquid, yield 42%; ^1^H NMR (500 MHz, CDCl_3_) δ 7.85 (dd, *J* = 7.8, 1.8 Hz, 1H), 7.45 (ddd, *J* = 8.6, 7.1, 1.8 Hz, 1H), 7.00 – 6.94 (m, 2H), 4.19 (ddd, *J* = 12.7, 6.0, 3.2 Hz, 1H), 2.76 – 2.62 (m, 2H), 2.01 – 1.94 (m, 1H), 1.83 – 1.68 (m, 5H), 1.35 – 1.05 (m, 5H). ^13^C NMR (126 MHz, CDCl_3_) δ 193.31, 162.00, 136.02, 126.98, 121.12, 121.10, 117.99, 82.05, 41.84, 40.31, 28.33, 28.26, 26.03, 25.97. HRMS (ESI) [M+H]^+^ calcd for C_15_H_19_O_2_: 231.1378, found: 231.1380.

**2-cycloheptylchroman-4-one (4k).** A yellow liquid, yield 45%; ^1^H NMR (500 MHz, CDCl_3_) δ 7.87 – 7.84 (m, 1H), 7.46 (ddd, *J* = 8.3, 7.2, 1.8 Hz, 1H), 7.01 – 6.95 (m, 2H), 4.28 (ddd, *J* = 13.5, 5.3, 2.7 Hz, 1H), 2.73 (dd, *J* = 16.6, 13.5 Hz, 1H), 2.62 (dd, *J* = 16.6, 2.7 Hz, 1H), 1.97 – 1.90 (m, 1H), 1.90 – 1.79 (m, 2H), 1.78 – 1.70 (m, 2H), 1.65 – 1.54 (m, 3H), 1.53 – 1.46 (m, 3H), 1.45 – 1.33 (m, 2H). ^13^C NMR (126 MHz, CDCl_3_) δ 193.53, 162.12, 136.05, 127.01, 121.13, 121.02, 118.02, 82.35, 43.05, 39.90, 29.61, 29.26, 28.45, 28.43, 26.81, 26.63. HRMS (ESI) [M+H]^+^ calcd for C_16_H_21_O_2_: 245.1528, found: 245.1536.

**2-(adamantan-1-yl)chroman-4-one (4l).** A yellow solid, yield 46%; m. p. 83.3-84.5 ℃; ^1^H NMR (500 MHz, CDCl_3_) δ 7.85 (dd, *J* = 8.1, 1.8 Hz, 1H), 7.45 (ddd, *J* = 8.7, 7.2, 1.8 Hz, 1H), 7.00 – 6.95 (m, 2H), 3.90 (dd, *J* = 14.1, 2.6 Hz, 1H), 2.71 (dd, *J* = 16.5, 14.1 Hz, 1H), 2.61 (dd, *J* = 16.5, 2.6 Hz, 1H), 2.07 – 2.03 (m, 3H), 1.77 – 1.74 (m, 6H), 1.69 (dq, *J* = 12.2, 2.1 Hz, 3H), 1.62 (dq, *J* = 12.2, 2.6 Hz, 3H). ^13^C NMR (126 MHz, CDCl_3_ δ 194.05, 162.38, 135.98, 126.95, 121.04, 120.96, 118.02, 85.59, 37.80, 37.20, 37.12, 35.97, 28.21. HRMS (ESI) [M+H]^+^ calcd for C_19_H_23_O_2_: 283.1690, found: 283.1693.

**2-benzylchroman-4-one (4m).** A yellow solid, yield 27%, m. p. 55.8-56.3 ℃; ^1^H NMR (500 MHz, CDCl_3_ ) δ 7.74 (dd, *J* = 7.8, 1.7 Hz, 1H), 7.35 (ddd, *J* = 8.6, 7.2, 1.8 Hz, 1H), 7.23 – 7.18 (m, 2H), 7.17 – 7.11 (m, 3H), 6.90 – 6.84 (m, 2H), 4.55 (dq, *J* = 9.6, 6.3 Hz, 1H), 3.09 (dd, *J* = 13.9, 6.3 Hz, 1H), 2.91 (dd, *J* = 13.9, 6.5 Hz, 1H), 2.57 – 2.54 (m, 2H). ^13^C NMR (126 MHz, CDCl_3_) δ 192.37, 161.43, 136.11, 136.05, 129.59, 128.58, 126.93, 121.32, 120.96, 117.99, 78.27, 42.17, 41.15. HRMS (ESI) [M+H]^+^ calcd for C_16_H_15_O_2_: 239.1057, found: 239.1067.

**2-(benzo[*d*][1,3]dioxol-2-yl)-6-methylchroman-4-one (5a).** A yellow solid, yield 42%, m. p. 120.6-121.3 ℃; ^1^H NMR (500 MHz, CDCl_3_) δ 7.67 (d, *J* = 2.3 Hz, 1H), 7.30 (dd, *J* = 8.5, 2.3 Hz, 1H), 6.93 (d, *J* = 8.4 Hz, 1H), 6.84 (s, 4H), 6.35 (d, *J* = 3.6 Hz, 1H), 4.74 – 4.67 (m, 1H), 2.94 (dd, *J* = 16.8, 12.8 Hz, 1H), 2.79 (dd, *J* = 16.8, 3.3 Hz, 1H), 2.30 (s, 3H). ^13^C NMR (126 MHz, CDCl_3_) δ 190.80, 158.62, 147.12, 146.86, 137.44, 131.60, 126.62, 122.14, 117.85, 108.87, 108.79, 108.69, 76.98, 36.52, 20.53. HRMS (ESI) [M+H]^+^ calcd for C_17_H_15_O_4_: 283.0962, found: 283.0965.

**2-(benzo[*d*][1,3]dioxol-2-yl)-7-methoxychroman-4-one (5b).** A yellow solid, yield 63%, m. p. 81.3-82.9 ℃; ^1^H NMR (500 MHz, CDCl_3_) δ 7.79 (d, *J* = 8.8 Hz, 1H), 6.87 – 6.79 (m, 4H), 6.57 (dd, *J* = 8.8, 2.4 Hz, 1H), 6.45 (d, *J* = 2.4 Hz, 1H), 6.31 (d, *J* = 3.7 Hz, 1H), 4.70 (dt, *J* = 12.8, 3.5 Hz, 1H), 3.79 (s, 3H), 2.89 (dd, *J* = 16.8, 12.9 Hz, 1H), 2.73 (dd, *J* = 16.8, 3.4 Hz, 1H). ^13^C NMR (126 MHz, CDCl_3_) δ 189.06, 166.26, 162.50, 147.11, 146.86, 128.72, 122.14, 114.89, 110.72, 108.88, 108.79, 108.64, 100.91, 77.27, 55.81, 36.19. HRMS (ESI) [M+H]^+^ calcd for C_17_H_15_O_5_: 299.0911, found: 299.0914.

**ethyl 2-(7-methoxy-4-oxochroman-2-yl)benzo[*d*][1,3]dioxole-5-carboxylate (5c).** A yellow liquid, yield 52%; ^1^H NMR (600 MHz, CDCl_3_) δ 7.78 (d, *J* = 8.8 Hz, 1H), 7.65 (ddd, *J* = 8.3, 5.5, 1.7 Hz, 1H), 7.47 (dd, *J* = 12.0, 1.7 Hz, 1H), 6.84 (dd, *J* = 12.9, 8.2 Hz, 1H), 6.57 (dd, *J* = 8.8, 2.4 Hz, 1H), 6.41 (t, *J* = 2.4 Hz, 1H), 6.39 (dd, *J* = 3.5, 2.3 Hz, 1H), 4.72 (dtd, *J* = 12.9, 3.4, 1.2 Hz, 1H), 4.34 – 4.29 (m, 2H), 3.79 (s, 3H), 2.89 (ddd, *J* = 17.7, 12.9, 4.9 Hz, 1H), 2.72 (dd, *J* = 16.7, 3.3 Hz, 1H), 1.35 (td, *J* = 7.2, 2.7 Hz, 3H). ^13^C NMR (151 MHz, CDCl_3_) δ 188.74, 166.32, 165.88, 162.37, 150.87, 147.20, 128.74, 125.58, 125.00, 114.80, 110.77, 109.81, 109.58, 108.03, 100.90, 61.08, 55.81, 36.12, 14.41. HRMS (ESI) [M+H]^+^ calcd for C_20_H_19_O_7_: 371.1119, found: 371.1125.

**2-(5-butyrylbenzo[*d*][1,3]dioxol-2-yl)-7-methoxychroman-4-one (5d).** A yellow liquid, yield 64%; ^1^H NMR (500 MHz, CDCl_3_) δ 7.75 (d, *J* = 8.8 Hz, 1H), 7.54 (ddd, *J* = 8.3, 5.2, 1.7 Hz, 1H), 7.41 (dd, *J* = 13.3, 1.7 Hz, 1H), 6.83 (dd, *J* = 11.2, 8.2 Hz, 1H), 6.54 (dd, *J* = 8.8, 2.4 Hz, 1H), 6.38 (dd, *J* = 3.4, 2.1 Hz, 2H), 4.71 (dtd, *J* = 12.9, 3.4, 1.0 Hz, 1H), 3.77 (s, 3H), 2.91 – 2.85 (m, 1H), 2.84 – 2.80 (m, 2H), 2.70 (ddd, *J* = 16.8, 3.4, 2.4 Hz, 1H), 1.74 – 1.65 (m, 2H), 0.94 (td, *J* = 7.4, 1.7 Hz, 3H). ^13^C NMR (126 MHz, CDCl_3_) δ 198.49, 188.70, 166.30, 162.33, 151.14, 150.91, 147.92, 147.65, 132.34, 128.70, 124.52, 114.77, 110.73, 109.84, 107.95, 100.90, 100.88, 55.81, 40.35, 36.06, 18.01, 13.99. HRMS (ESI) [M+H]^+^ calcd for C_21_H_21_O_6_: 369.1330, found: 369.1333.

**2-(7-methoxy-4-oxochroman-2-yl)benzo[*d*][1,3]dioxole-5-carbonitrile (5e).** A yellow solid, yield 62%, m. p. 92.4-93.7 ℃; ^1^H NMR (500 MHz, CDCl_3_) δ 7.78 (d, *J* = 8.8 Hz, 1H), 7.25-7.20 (m, 1H), 7.04 (dd, *J* = 16.7, 1.6 Hz, 1H), 6.88 (dd, *J* = 15.9, 8.1 Hz, 1H), 6.58 (ddd, *J* = 8.8, 2.4, 1.2 Hz, 1H), 6.42 (dd, *J* = 3.2, 0.7 Hz, 1H), 6.38 (dd, *J* = 2.4, 1.2 Hz, 1H), 4.73 (dtd, *J* = 12.7, 3.3, 1.1 Hz, 1H), 3.79 (s, 3H), 2.88 (ddd, *J* = 16.8, 12.7, 3.1 Hz, 1H), 2.72 (ddd, *J* = 16.7, 3.4, 2.6 Hz, 1H). ^13^C NMR (126 MHz, CDCl_3_) δ 188.44, 166.40, 162.23, 151.21, 151.00, 147.83, 147.58, 128.80, 128.66, 118.80, 114.74, 111.54, 110.86, 110.40, 105.47, 100.90, 76.91, 55.88, 36.15. HRMS (ESI) [M+H]^+^ calcd for C_18_H_14_O_5_N: 324.0857, found: 324.0866.

**2-(benzo[*d*][1,3]dioxol-2-yl)-6-chlorochroman-4-one (5f).** A yellow solid, yield 42%, m. p. 117.1-118.0 ℃; ^1^H NMR (500 MHz, CDCl_3_) δ 7.83 (d, *J* = 2.7 Hz, 1H), 7.42 (dd, *J* = 8.9, 2.7 Hz, 1H), 6.99 (d, *J* = 8.9 Hz, 1H), 6.86 – 6.82 (m, 4H), 6.36 (d, *J* = 3.4 Hz, 1H), 4.74 (dt, *J* = 12.4, 3.5 Hz, 1H), 2.95 (dd, *J* = 16.9, 12.5 Hz, 1H), 2.84 (dd, *J* = 16.9, 3.5 Hz, 1H). ^13^C NMR (126 MHz, CDCl_3_) δ 189.38, 158.94, 147.01, 146.73, 136.18, 126.38, 122.25, 121.84, 119.80, 108.90, 108.83, 108.46, 77.15, 36.17. HRMS (ESI) [M+H]^+^ calcd for C_16_H_12_O_4_Cl: 303.0411, found: 303.0419.

**2-(benzo[*d*][1,3]dioxol-2-yl)-7-fluorochroman-4-one (5g).** A yellow solid, yield 67%, m. p. 110.8-111.9 ℃; ^1^H NMR (500 MHz, CDCl_3_) δ 7.51 (dd, *J* = 8.1, 3.2 Hz, 1H), 7.20 (ddd, *J* = 9.1, 7.7, 3.2 Hz, 1H), 7.00 (dd, *J* = 9.1, 4.2 Hz, 1H), 6.86 – 6.78 (m, 4H), 6.34 (d, *J* = 3.4 Hz, 1H), 4.71 (dt, *J* = 12.6, 3.4 Hz, 1H), 2.94 (dd, *J* = 17.0, 12.6 Hz, 1H), 2.82 (dd, *J* = 16.9, 3.5 Hz, 1H). ^13^C NMR (126 MHz, CDCl_3_) δ 189.05, 167.59 (d, *J*_C-F_ = 256.8 Hz), 162.14 (d, *J*_C-F_ = 13.6 Hz), 147.03, 146.75, 129.60 (d, *J*_C-F_ = 11.4 Hz), 122.24 (d, *J*_C-F_ = 1.8 Hz), 118.01 (d, *J*_C-F_ = 2.5 Hz), 110.53 (d, *J*_C-F_ = 22.7 Hz), 108.89, 108.82, 108.41, 105.11, 104.91, 77.49, 36.14. ^19^F NMR (471 MHz, CDCl_3_) δ -120.26. HRMS (ESI) [M+H]^+^ calcd for C_16_H_12_O_4_F: 287.0713, found: 287.0714.

**2-(benzo[*d*][1,3]dioxol-2-yl)-6-fluorochroman-4-one (5h).** A yellow solid, yield 41%, m. p. 107.7-108.5 ℃; ^1^H NMR (500 MHz, CDCl_3_) δ 7.90 (dd, *J* = 8.8, 6.5 Hz, 1H), 6.87 – 6.80 (m, 4H), 6.78 – 6.69 (m, 2H), 6.35 (d, *J* = 3.5 Hz, 1H), 4.76 (dt, *J* = 12.6, 3.4 Hz, 1H), 2.95 (dd, *J* = 16.9, 12.6 Hz, 1H), 2.81 (dd, *J* = 16.9, 3.5 Hz, 1H). ^13^C NMR (126 MHz, CDCl_3_) δ 189.05, 167.59 (d, *J* = 256.8 Hz), 162.14 (d, *J* = 13.6 Hz), 147.03, 146.75, 129.60 (d, *J* = 11.4 Hz), 122.24 (d, *J* = 1.8 Hz), 118.01 (d, *J* = 2.5 Hz), 110.53 (d, *J* = 22.7 Hz), 108.41, 104.91, 77.49, 36.14. ^19^F NMR (471 MHz, CDCl_3_) δ -99.45 – -99.55 (m). HRMS (ESI) [M+H]^+^ calcd for C_16_H_12_O_4_F: 287.0712, found: 287.0714.

**(E)-(2-(benzo[*d*][1,3]dioxol-2-yl)chroman-4-ylidene)hydrazine (A1).** A yellow solid, yield 95%, m. p. 114.6-115.3 ℃; ^1^H NMR (500 MHz, CDCl_3_) δ 7.89 (dd, *J* = 8.1, 1.7 Hz, 1H), 7.19 (ddd, *J* = 8.3, 7.2, 1.7 Hz, 1H), 7.00 – 6.94 (m, 2H), 6.85 (d, *J* = 1.4 Hz, 4H), 6.29 (d, *J* = 4.1 Hz, 1H), 5.34 (s, 2H), 4.36 (ddd, *J* = 11.9, 4.2, 3.5 Hz, 1H), 2.93 (dd, *J* = 16.2, 3.5 Hz, 1H), 2.47 (dd, *J* = 16.3, 11.9 Hz, 1H). ^13^C NMR (126 MHz, CDCl_3_) δ 154.75, 147.26, 147.05, 140.03, 130.00, 123.87, 122.33, 122.14, 122.09, 121.49, 117.69, 109.16, 109.00, 108.83, 74.64, 22.33. HRMS (ESI) [M+H]^+^ calcd for C_16_H_15_N_2_O_3_: 283.1066, found: 283.1077.

**(E)-2-(benzo[*d*][1,3]dioxol-2-yl)chroman-4-one oxime (B1).** A white solid, yield 92%, m. p. 138.2-139.7 ℃; ^1^H NMR (500 MHz, CDCl_3_) δ 8.54 (s, 1H), 7.80 (dd, *J* = 7.9, 1.6 Hz, 1H), 7.29 (ddd, *J* = 8.6, 7.2, 1.7 Hz, 1H), 7.03 – 6.96 (m, 2H), 6.90 – 6.81 (m, 4H), 6.33 (d, *J* = 4.0 Hz, 1H), 4.40 (dt, *J* = 12.1, 3.7 Hz, 1H), 3.48 (dd, *J* = 17.1, 3.4 Hz, 1H), 2.73 (dd, *J* = 17.1, 12.1 Hz, 1H). ^13^C NMR (126 MHz, CDCl_3_) δ 155.42, 148.68, 147.26, 147.03, 131.57, 123.92, 122.16, 122.03, 118.05, 109.09, 108.92, 108.79, 74.74, 22.33.

HRMS (ESI) [M+H]^+^ calcd for C_16_H_14_NO_4_: 284.0917, found: 284.0917.

**2-(benzo[d][1,3]dioxol-2-yl)chroman-4-ol (C1).** A yellow liquid, yield 95%; ^1^H NMR (600 MHz, CDCl_3_) δ 7.42 (dt, *J* = 7.6, 1.4 Hz, 1H), 7.18 (ddt, *J* = 8.0, 7.2, 1.1 Hz, 1H), 6.96 (td, *J* = 7.5, 1.2 Hz, 1H), 6.88 (dd, *J* = 8.3, 1.2 Hz, 1H), 6.85 (s, 4H), 6.27 (d, *J* = 4.5 Hz, 1H), 4.89 (dd, *J* = 9.6, 5.9 Hz, 1H), 4.39 (ddd, *J* = 10.5, 4.5, 2.6 Hz, 1H), 2.76 (s, 1H), 2.39 (ddd, *J* = 13.1, 5.9, 2.6 Hz, 1H), 2.05 – 1.96 (m, 1H). ^13^C NMR (151 MHz, CDCl_3_) δ 153.23, 147.35, 147.12, 129.50, 127.34, 125.58, 121.97, 121.95, 121.38, 116.89, 116.84, 109.49, 108.68, 74.53, 64.29, 30.95. HRMS (ESI) [M+Na]^+^ calcd for C_16_H_14_O_4_Na: 293.0783, found: 293.0784.

**(E)-2-(benzo[*d*][1,3]dioxol-2-yl)-3-benzylidenechroman-4-one (C1).** A yellow solid, yield 85%, m. p. 130.2-131.6 ℃; ^1^H NMR (500 MHz, CDCl_3_) δ 8.04 – 8.00 (m, 2H), 7.49 – 7.46 (m, 2H), 7.45 – 7.41 (m, 3H), 7.41 – 7.38 (m, 1H), 7.05 (ddd, *J* = 8.1, 7.2, 1.1 Hz, 1H), 6.81 – 6.75 (m, 2H), 6.74 – 6.70 (m, 2H), 6.52 (dd, *J* = 8.0, 1.3 Hz, 1H), 6.44 (d, *J* = 3.1 Hz, 1H), 5.87 (dd, *J* = 3.1, 1.1 Hz, 1H). ^13^C NMR (126 MHz, CDCl_3_) δ 181.60, 159.40, 147.02, 146.67, 142.15, 136.09, 134.00, 130.01, 129.91, 129.08, 127.96, 127.33, 122.04, 121.93, 121.88, 121.66, 117.53, 110.02, 108.53, 76.55. HRMS (ESI) [M+H]^+^ calcd for C_23_H_17_O_4_: 357.1113, found: 357.1121.

***N*-benzyl-*N*-methylbenzo[*d*][1,3]dioxole-5-carboxamide (M1).** A yellow solid, yield 90%, m. p. 108.6-109.4 ℃; ^1^H NMR (500 MHz, CDCl_3_) δ 7.31 – 7.27 (m, 2H), 7.23 – 7.19 (m, 2H), 7.16 – 7.11 (m, 2H), 6.77 (dd, *J* = 7.9, 0.6 Hz, 1H), 6.49 (t, *J* = 5.6 Hz, 1H), 5.99 (s, 2H), 4.53 (d, *J* = 5.6 Hz, 2H), 2.33 (s, 3H). ^13^C NMR (126 MHz, CDCl_3_) δ 166.74, 150.39, 148.02, 137.36, 135.33, 129.51, 128.73, 128.01, 121.68, 108.05, 107.80, 101.77, 44.00, 21.23. HRMS (ESI) [M+H]^+^ calcd for C_16_H_16_O_3_N: 270.1121, found: 270.1125.

**ethyl benzo[*d*][1,3]dioxole-5-carboxylate (M2).** A yellow liquid, yield 88%; ^1^H NMR (500 MHz, CDCl_3_) δ 7.62 (dd, *J* = 8.2, 1.7 Hz, 1H), 7.43 (d, *J* = 1.7 Hz, 1H), 6.79 (d, *J* = 8.2 Hz, 1H), 5.99 (s, 2H), 4.30 (q, *J* = 7.1 Hz, 2H), 1.34 (t, *J* = 7.1 Hz, 3H). ^13^C NMR (126 MHz, CDCl_3_) δ 166.02, 151.54, 147.73, 125.27, 124.54, 109.51, 107.95, 101.85, 60.93, 14.40. HRMS (ESI) [M+H]^+^ calcd for C_10_H_11_O_4_: 195.0651, found: 195.0652.

# 11 ^1^H NMR, ^13^C NMR, ^19^F NMR, ^31^P NMR, HRMS spectra

**Figure S15.** ^1^H NMR Spectrum (CDCl_3_, 500 MHz) of **3a**.

**Figure S16.** ^13^C NMR Spectrum (CDCl_3_, 126 MHz) of **3a**.

**Figure S17.** HRMS Spectrum of **3a.**

**Figure S18.** ^1^H NMR Spectrum (CDCl_3_, 500 MHz) of **3b**.

**Figure S19.** ^13^C NMR Spectrum (CDCl_3_, 126 MHz) of **3b**.

**Figure S20.** HRMS Spectrum of **3b**.

**Figure S21.** ^1^H NMR Spectrum (CDCl_3_, 500 MHz) of **3c**.

**Figure S22.** ^13^C NMR Spectrum (CDCl_3_, 126 MHz) of **3c**.

**Figure S23.** HRMS Spectrum of **3c**.

**Figure 24.** ^1^H NMR Spectrum (CDCl_3_, 500 MHz) of **3d**.

**Figure S25.** ^13^C NMR Spectrum (CDCl_3_, 126 MHz) of **3d**.

**Figure S26.** HRMS Spectrum of **3d**.

**Figure S27.** ^1^H NMR Spectrum (CDCl_3_, 400 MHz) of **3e**.

**Figure S28.** ^13^C NMR Spectrum (CDCl_3_, 101 MHz) of **3e**.

**Figure S29.** HRMS Spectrum of **3e**.

**Figure S30.** ^1^H NMR Spectrum (CDCl_3_, 500 MHz) of **3f**.

**Figure S31.** ^13^C NMR Spectrum (CDCl_3_, 126 MHz) of **3f**.

**Figure S32.** HRMS Spectrum of **3f**.

**Figure S33.** ^1^H NMR Spectrum (CDCl_3_, 500 MHz) of **3g**.

**Figure S34.** ^13^C NMR Spectrum (CDCl_3_, 126 MHz) of **3g**.

**Figure S35.** HRMS Spectrum of **3g**.

**Figure S36.** ^1^H NMR Spectrum (CDCl_3_, 500 MHz) of **3h**.

**Figure S37.** ^13^C NMR Spectrum (CDCl_3_, 126 MHz) of **3h**.

**Figure S38.** HRMS Spectrum of **3h**.

**Figure S39.** ^1^H NMR Spectrum (CDCl_3_, 500 MHz) of **3i**.

**Figure S40.** ^13^C NMR Spectrum (CDCl_3_, 126 MHz) of **3i**.

**Figure S41.** HRMS Spectrum of **3i**.

**Figure S42.** ^1^H NMR Spectrum (CF_3_CO_2_D, 500 MHz) of **3j**.

**Figure S43.** ^13^C NMR Spectrum (CDCl_3_, 126 MHz) of **3j**.

**Figure S44.** HRMS Spectrum of **3j**.

**Figure S45.** ^1^H NMR Spectrum (CDCl_3_, 500 MHz) of **3k**.

**Figure S46.** ^13^C NMR Spectrum (CDCl_3_, 126 MHz) of **3k**.

**Figure S47.** HRMS Spectrum of **3k**.

**Figure S48.** ^1^H NMR Spectrum (CDCl_3_, 600 MHz) of **3l**.

**Figure S49.** ^13^C NMR Spectrum (CF_3_CO_2_D, 151 MHz) of **3l**.

**Figure S50.** HRMS Spectrum of **3l**.

**Figure S51.** ^1^H NMR Spectrum (CDCl_3_, 600 MHz) of **3m**.

**Figure S52.** ^13^C NMR Spectrum (CDCl_3_, 151 MHz) of **3m**.

**Figure S53.** HRMS Spectrum of **3m**.

**Figure S54.** ^1^H NMR Spectrum (CDCl_3_, 500 MHz) of **3n**.

**Figure S55.** ^13^C NMR Spectrum (CDCl_3_, 126 MHz) of **3n**.

**Figure S56.** HRMS Spectrum of **3n**.

**Figure S57.** ^1^H NMR Spectrum (CDCl_3_, 500 MHz) of **3oA**.

**Figure S58.** ^13^C NMR Spectrum (CDCl_3_, 126 MHz) of **3oA**.

**Figure S59.** HRMS Spectrum of **3oA**.

**Figure S60.** ^1^H NMR Spectrum (CDCl_3_, 500 MHz) of **3oB**.

**Figure S61.** ^13^C NMR Spectrum (CDCl_3_, 126 MHz) of **3oB**.

**Figure S62.** HRMS Spectrum of **3oB**.

**Figure S63.** ^1^H NMR Spectrum (CDCl_3_, 500 MHz) of **3pA**.

**Figure S64.** ^13^C NMR Spectrum (CDCl_3_, 126 MHz) of **3pA**.

**Figure S65.** HRMS Spectrum of **3pA**.

**Figure S66.** ^1^H NMR Spectrum (CDCl_3_, 500 MHz) of **3pB**.

**Figure S67.** ^13^C NMR Spectrum (CDCl_3_, 126 MHz) of **3pB**.

**Figure S68.** HRMS Spectrum of **3pB**.

**Figure S69.** ^1^H NMR Spectrum (CDCl_3_, 500 MHz) of **3q**.

**Figure S70.** ^13^C NMR Spectrum (CDCl_3_, 126 MHz) of **3q**.

**Figure S71.** HRMS Spectrum of **3q**.

**Figure S72.** ^1^H NMR Spectrum (CDCl_3_, 500 MHz) of **3r**, **Isomer 1**.

**Figure S73.** ^13^C NMR Spectrum (CDCl_3_, 126 MHz) of **3r**, **Isomer 1**.

**Figure S74.** HRMS Spectrum of **3r**, **Isomer 1**.

**Figure S75.** ^1^H NMR Spectrum (CDCl_3_, 500 MHz) of **3r**, **Isomer 2**.

**Figure S76.** ^13^C NMR Spectrum (CDCl_3_, 126 MHz) of **3r**, **Isomer 2**.

**Figure S77.** HRMS Spectrum of **3r**, **Isomer 2**.

**Figure S78.** ^1^H NMR Spectrum (CDCl_3_, 400 MHz) of **3s**, **Isomer 1**.

**Figure S79.** ^13^C NMR Spectrum (CDCl_3_, 101 MHz) of **3s**, **Isomer 1**.

**Figure S80.** HRMS Spectrum of **3s**, **Isomer 1**.

**Figure S81.** ^1^H NMR Spectrum (CDCl_3_, 400 MHz) of **3s**, **Isomer 2**.

**Figure S82.** ^13^C NMR Spectrum (CDCl_3_, 101 MHz) of **3s**, **Isomer 2**.

**Figure S83.** HRMS Spectrum of **3s**, **Isomer 2**.


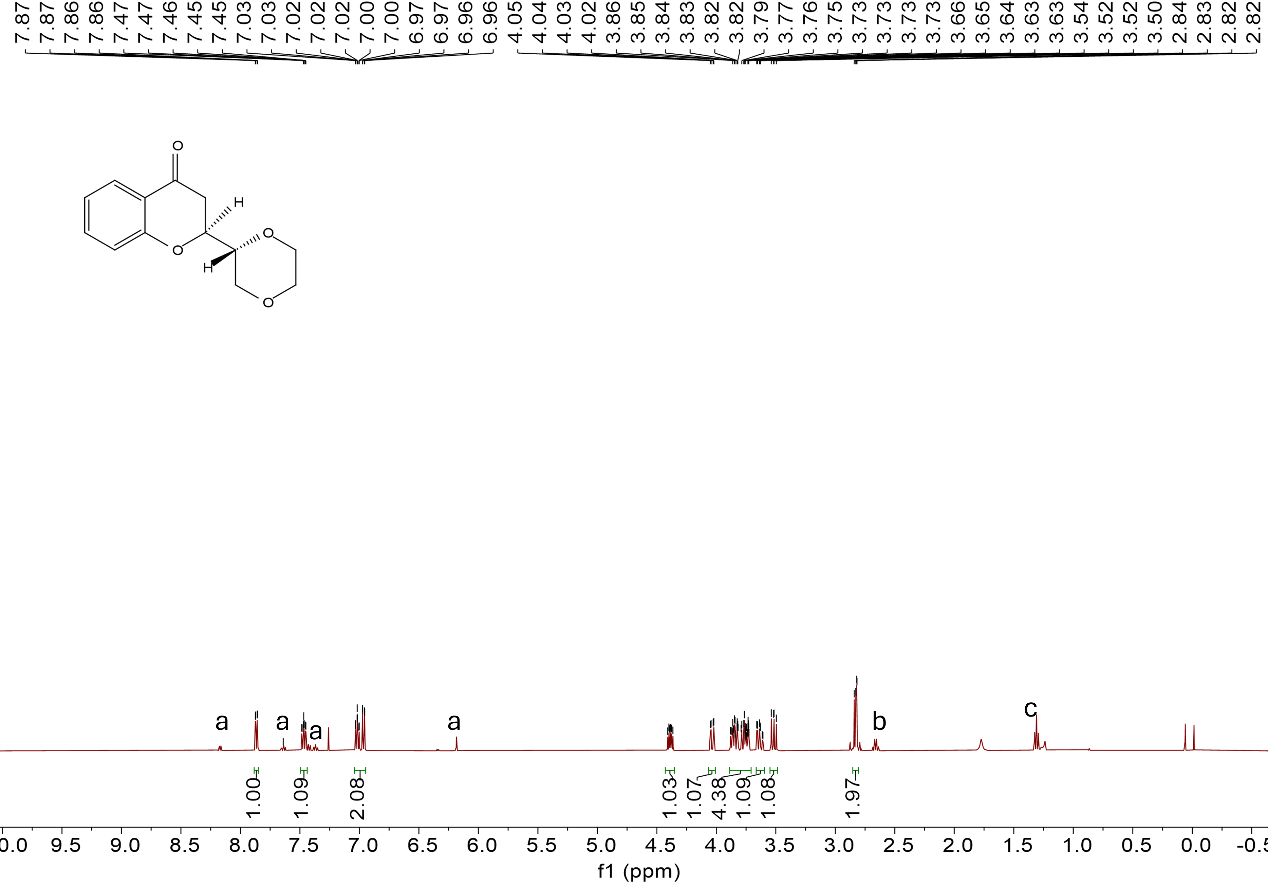


**Figure S84.** ^1^H NMR Spectrum (CDCl_3_, 500 MHz) of **3t**, **Isomer 1**. The peaks labeled a originate from the starting chromone, b from ethyl acetate, and c from petroleum ether.

**Figure S85.** ^13^C NMR Spectrum (CDCl_3_, 126 MHz) of **3t**, **Isomer 1**.

**Figure S86.** HRMS Spectrum of **3t**, **Isomer 1**.

**Figure S87.** ^1^H NMR Spectrum (CDCl_3_, 500 MHz) of **3t’**, **Isomer 2**.

**Figure S88.** ^13^C NMR Spectrum (CDCl_3_, 126 MHz) of **3t’**, **Isomer 2**.

**Figure S89.** HRMS Spectrum of **3t’**, **Isomer 2**.

**Figure S90.** ^1^H NMR Spectrum (CDCl_3_, 500 MHz) of **3u**.

**Figure S91.** ^13^C NMR Spectrum (CDCl_3_, 126 MHz) of **3u**.

**Figure S92.** HRMS Spectrum of **3u**.

**Figure S93.** ^1^H NMR Spectrum (CDCl_3_, 500 MHz) of **3v**.

**Figure S94.** ^13^C NMR Spectrum (CDCl_3_, 126 MHz) of **3v**.

**Figure S95.** HRMS Spectrum of **3v**.

**Figure S96.** ^1^H NMR Spectrum (CDCl_3_, 600 MHz) of **3w**.

**Figure S97.** ^13^C NMR Spectrum (CDCl_3_, 151 MHz) of **3w**.

**Figuer S98.** HRMS Spectrum of **3w**.

**Figure S99.** ^1^H NMR Spectrum (CDCl_3_, 500 MHz) of **3x**.

**Figure S100.** ^13^C NMR Spectrum (CDCl_3_, 126 MHz) of **3x**.

**Figure S101.** HRMS Spectrum of **3x**.

**Figure S102.** ^1^H NMR Spectrum (CDCl_3_, 600 MHz) of **4a**

**Figure S103.** ^13^C NMR Spectrum (CDCl_3_, 151 MHz) of **4a**.

**Figure S104.** HRMS Spectrum of **4a**.

**Figure S105.** ^1^H NMR Spectrum (CDCl_3_, 500 MHz) of **4b**.

**Figure S106.** ^13^C NMR Spectrum (CDCl_3_, 126 MHz) of **4b**.

**Figure S107.** ^31^P NMR Spectrum (CDCl_3_, 202 MHz) of **4b**.

**Figure S108.** HRMS Spectrum of **4b**.

**Figure S109.** ^1^H NMR Spectrum (CDCl_3_, 400 MHz) of **4c**.

**Figure S110.** ^13^C NMR Spectrum (CDCl_3_, 101 MHz) of **4c**.

**Figure S111.** ^31^P NMR Spectrum (CDCl_3_, 202 MHz) of **4c**.

**Figure S112.** HRMS Spectrum of **4c**.

**Figure S113.** ^1^H NMR Spectrum (CDCl_3_, 500 MHz) of **4d**.

**Figure S114.** ^13^C NMR Spectrum (CDCl_3_, 126 MHz) of **4d**.

**Figure S115.** ^31^P NMR Spectrum (CDCl_3_, 202 MHz) of **4d**.

**Figure S116.** HRMS Spectrum of **4d**.

**Figure S117.** ^1^H NMR Spectrum (CDCl_3_, 500 MHz) of **4e**.

**Figure S118.** ^13^C NMR Spectrum (CDCl_3_, 126 MHz) of **4e**.

**Figure S119.** ^31^P NMR Spectrum (CDCl_3_, 126 MHz) of **4e**.

**Figure S120.** HRMS Spectrum of **4e**.

**Figure S121.** ^1^H NMR Spectrum (CDCl_3_, 500 MHz) of **4f**.

**Figure S122.** ^13^C NMR Spectrum (CDCl_3_, 126 MHz) of **4f**.

**Figure S123.** HRMS Spectrum of **4f**.

**Figure S124.** ^1^H NMR Spectrum (CDCl_3_, 500 MHz) of **4g**.

**Figure S125.** ^13^C NMR Spectrum (CDCl_3_, 126 MHz) of **4g**.

**Figure S126.** HRMS Spectrum of **4g**.

**Figure S127.** ^1^H NMR Spectrum (CDCl_3_, 600 MHz) of **4h**, **Isomer1**.

**Figure S128.** ^13^C NMR Spectrum (CDCl_3_, 151 MHz) of **4h**, **Isomer1**.

**Figure S129.** HRMS Spectrum of **4h**, **Isomer1**.

**Figure S130.** ^1^H NMR Spectrum (CDCl_3_, 600 MHz) of **4h**, **Isomer2**.

**Figure S131.** ^13^C NMR Spectrum (CDCl_3_, 151 MHz) of **4h**, **Isomer2**.

**Figure S132.** HRMS Spectrum of **4h**, **Isomer2**.

**Figure S133.** ^1^H NMR Spectrum (CDCl_3_, 500 MHz) of **4i**.

**Figure S134.** ^13^C NMR Spectrum (CDCl_3_, 126 MHz) of **4i**.

**Figure S135.** HRMS Spectrum of **4i**.

**Figure S136.** ^1^H NMR Spectrum (CDCl_3_, 500 MHz) of **4j**.

**Figure S137.** ^13^C NMR Spectrum (CDCl_3_, 126 MHz) of **4j**.

**Figure S138.** HRMS Spectrum of **4j**.

**Figure S139.** ^1^H NMR Spectrum (CDCl_3_, 500 MHz) of **4k**.

**Figure S140.** ^13^C NMR Spectrum (CDCl_3_, 126 MHz) of **4k**.

**Figure S141.** HRMS Spectrum of **4k**.

**Figure S142.** ^1^H NMR Spectrum (CDCl_3_, 500 MHz) of **4l**.

**Figure S143.** ^13^C NMR Spectrum CDCl_3_, 126 MHz) of **4l**.

**Figure S144.** HRMS Spectrum of **4l**.

**Figure S145.** ^1^H NMR Spectrum (CDCl_3_, 500 MHz) of **4m**.

**Figure S146.** ^13^C NMR Spectrum (CDCl_3_, 126 MHz) of **4m**.

**Figure S147.** HRMS Spectrum of **4m**.

**Figure S148.** ^1^H NMR Spectrum (CDCl_3_, 500 MHz) of **5a**.

**Figure S149.** ^13^C NMR Spectrum (CDCl_3_, 126 MHz) of **5a**.

**Figure S150.** HRMS Spectrum of **5a**.

**Figure S151.** ^1^H NMR Spectrum (CDCl_3_, 500 MHz) of **5b**.

**Figure S152.** ^13^C NMR Spectrum (CDCl_3_, 126 MHz) of **5b**.

**Figure S153.** HRMS Spectrum of **5b**.

**Figure S154.** ^1^H NMR Spectrum (CDCl_3_, 600 MHz) of **5c**.

**Figure S155.** ^13^C NMR Spectrum (CDCl_3_, 151 MHz) of **5c**.

**Figure S156.** HRMS Spectrum of **5c**.

**Figure S157.** ^1^H NMR Spectrum (CDCl_3_, 500 MHz) of **5d**.

**Figure S158.** ^13^C NMR Spectrum (CDCl_3_, 126 MHz) of **5d.**

**Figure S159.** HRMS Spectrum of **5d**.

**Figure S160.** ^1^H NMR Spectrum (CDCl_3_, 500 MHz) of **5e**.

**Figure S161.** ^13^C NMR Spectrum (CDCl_3_, 126 MHz) of **5e**.

**Figure S162.** HRMS Spectrum of **5e**.

**Figure S163.** ^1^H NMR Spectrum (CDCl_3_, 500 MHz) of **5f**.

**Figure S164.** ^13^C NMR Spectrum (CDCl_3_, 126 MHz) of **5f**.

**Figure S165.** HRMS Spectrum of **5f**.

**Figure S166.** ^1^H NMR Spectrum (CDCl_3_, 500 MHz) of **5g**.

**Figure S167.** ^13^C NMR Spectrum (CDCl_3_, 126 MHz) of **5g**.

**Figure S168.** ^19^F NMR Spectrum (CDCl_3_, 471 MHz) of **5g**.

**Figure S169.** HRMS Spectrum of **5g**.

**Figure S170.** ^1^H NMR Spectrum (CDCl_3_, 500 MHz) of **5h**.

**Figure S171.** ^13^C NMR Spectrum (CDCl_3_, 126 MHz) of **5h**.

**Figure S172.** ^19^F NMR Spectrum (CDCl_3_, 471 MHz) of **5h**.

**Figure S173.** HRMS Spectrum of **5h**.

**Figure S174.** ^1^H NMR Spectrum (CDCl_3_, 500 MHz) of **A1**.

**Figure S175.** ^13^C NMR Spectrum (CDCl_3_, 126 MHz) of **A1**.

**Figure S176.** HRMS Spectrum of **A1**.

**Figure S177.** ^1^H NMR Spectrum (CDCl_3_, 500 MHz) of **B1**.

**Figure S178.** ^13^C NMR Spectrum (CDCl_3_, 126 MHz) of **B1**.

**Figure S179.** HRMS Spectrum of **B1**.

**Figure S180.** ^1^H NMR Spectrum (CDCl_3_, 600 MHz) of **C1**.

**Figure S181.** ^13^C NMR Spectrum (CDCl_3_, 151 MHz) of **C1**.

**Figure S182.** HRMS Spectrum of **C1**.

**Figure S183.** ^1^H NMR Spectrum (CDCl_3_, 500 MHz) of **D1**.

**Figure S184.** ^13^C NMR Spectrum (CDCl_3_, 126 MHz) of **D1**.

**Figure S185.** HRMS Spectrum of **D1**.

**Figure S186.** ^1^H NMR Spectrum (CDCl_3_, 500 MHz) of **M1**.

**Figure S187.** ^13^C NMR Spectrum (DMSO-*d*_6_, 126 MHz) of **M1**.

**Figure S188.** HRMS Spectrum of **M1**.

**Figure S189** ^1^H NMR Spectrum (CDCl_3_, 500 MHz) of **M2**.

**Figure S190.** ^13^C NMR Spectrum (CDCl_3_, 126 MHz) of **M2**.

**Figure S191.** HRMS Spectrum of **M2**.

# Reference

1. Frisch, M. J., Trucks, G. W., Schlegel, H. B., Scuseria, G. E., Robb, M. A., Cheeseman, J. R., Scalmani, G., Barone, V., Petersson, G. A., Nakatsuji, H., Li, X., Caricato, M., Marenich, A. V., Bloino, J., Janesko, B. G., Gomperts, R., Mennucci, B., Hratchian, H. P., Ortiz, J. V., Izmaylov, A. F., Sonnenberg, J. L.; Williams, Ding, F., Lipparini, F., Egidi, F., Goings, J., Peng, B., Petrone, A., Henderson, T., Ranasinghe, D., Zakrzewski, V. G., Gao, J., Rega, N., Zheng, G., Liang, W., Hada, M., Ehara, M., Toyota, K., Fukuda, R., Hasegawa, J., Ishida, M., Nakajima, T. Honda, Y., Kitao, O., Nakai, H., Vreven, T., Throssell, K., Montgomery Jr., J. A., Peralta, J. E., Ogliaro, F., Bearpark, M. J., Heyd, J. J., Brothers, E. N., Kudin, K. N., Staroverov, V. N., Keith, T. A., Kobayashi, R., Normand, J., Raghavachari, K., Rendell, A. P., Burant, J. C., Iyengar, S. S., Tomasi, J., Cossi, M., Millam, J. M., Klene, M., Adamo, C., Cammi, R., Ochterski, J. W., Martin, R. L., Morokuma, K.; Farkas, O., Foresman, J. B., Fox, D. J. Gaussian 16 Rev. A.03, Wallingford, CT, (2016).
2. Zhao, Y.; Truhlar, D. G. *Theor. Chem. Acc.* 2008, **120**, 215-241.
3. Zhao, Y.; Truhlar, D. G. *Acc. Chem. Res.* 2008, **41**, 157-167.
4. McLean, A. D.; Chandler, G. S. *J. Chem. Phys*. 1980, **72**, 5639-5648.
5. Binning, R. C.; Curtiss, L. A. *J. Comput. Chem.* 1990, **11**, 1206-1216.
6. Marenich, A. V.; Cramer, C. J.; Truhlar, D. G. *J. Phys. Chem.* 2009**,** **113**, 6378-6396.
7. Krishnan, R.; Binkley, J. S.; Seeger, R.; Pople, J. A. *J. Chem. Phys*. 1980, **72**, 650-654.
8. Clark, T.; Chandrasekhar, J.; Spitznagel, G. W.; Schleyer, P. V. R. *J. Comput. Chem*. 1983, **4**, 294-301.

9. J. S. Poh, D. N. Tran, C. Battilocchio, J. M. Hawkins and S. V. Ley, *Angew. Chem., Int. Edit.*, 2015, **54**, 7920-7923.

10. J. Kozłowska, B. Potaniec, B. Żarowska and M. Anioł, *Molecules*, 2017, **22**, 1485.

11. N. Boechat, J. C. S. da Costa, J. de Souza Mendonça, P. S. M. de Oliveira and M. Vinı́cius Nora De Souza, *Tetrahedron Lett.*, 2004, **45**, 6021-6022.

12. K. Takao, M. Yamashita, A. Yashiro and Y. Sugita, *Chem. Pharm. Bull.*, 2016, **14**, 1203-1207.

13. Cismesiaa, M. A, Yoon, T. P. *Chem. Sci*., 2015, **6**, 5426–5434.

14. Yasuda, M. Yamasaki, S. Onishi, Y. & Baba, A, *J. Am.Chem. Soc.*, 2004 **126**, 7186–7187.

15. Goldstein S, Rabani J. *J. Photochem. Photobiol. A: Chem.*, 2008, **193**, 50-55.

16. Tripathi CB, Ohtani T, Corbett MT, Ooi T. *Chem. Sci.*, 2017, **8**, 5622-5627.
