# Supplementary figures and images for: Chromones as Photocatalyzed HAT Reagent and Michael Acceptor for Direct C–H Alkylation of 2-Substituted 4-Chromanones
Source: Research (Wash D C). 2026 May 11;9:1250. doi: 10.34133/research.1250 (PMC13158460; doi:10.34133/research.1250)

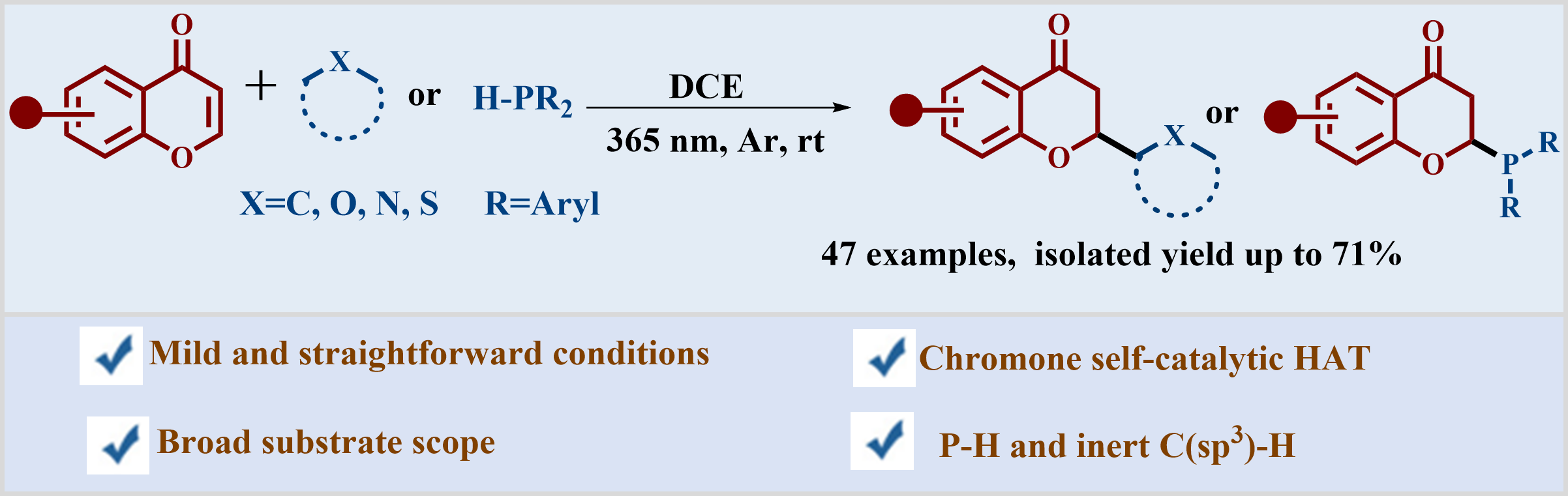

Supplement: Supplementary 1 — Graphical Abstract Supplementary Text Tables S1 to S14 Schemes S1 to S10 Figs. S1 to S191 [file research.1250.f1.zip › Graphical Abstract.tif]
